# Eschenmoser coupling reactions starting from primary thioamides. When do they work and when not?

**DOI:** 10.3762/bjoc.19.61

**Published:** 2023-06-09

**Authors:** Lukáš Marek, Jiří Váňa, Jan Svoboda, Jiří Hanusek

**Affiliations:** 1 Institute of Organic Chemistry and Technology, Faculty of Chemical Technology, University of Pardubice, Studentská 573, CZ532 10 Pardubice, Czech Republic

**Keywords:** Eschenmoser coupling reaction, Hantzsch thiazole synthesis, isoquinolin-4-ones, primary thioamides, structure–reactivity relationships

## Abstract

Reactions of thiobenzamide or thioacetamide with 4-bromo-1,1-dimethyl-1,4-dihydroisoquinoline-3(2*H*)-one, 4-bromoisoquinoline-1,3(2*H*,4*H*)-dione and two α-bromo(phenyl)acetamides were examined under various conditions (base, solvent, thiophile, temperature) and structure/medium features that influence product distribution (Eschenmoser coupling reaction, Hantzsch thiazole synthesis and elimination to nitriles) were identified. The key factor that enables the successful Eschenmoser coupling reaction involves the optimum balance in acidity of nitrogen and carbon atoms of the intermediary α-thioiminium salts.

## Introduction

**Scheme 1:**
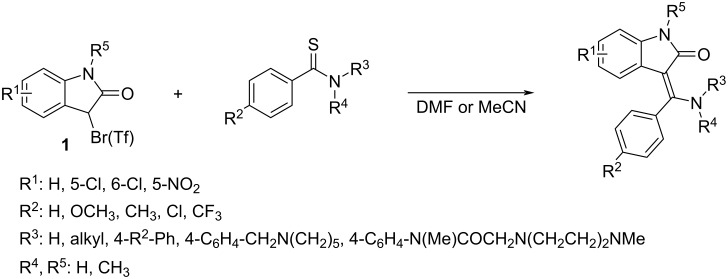
Eschenmoser coupling reaction between 3-substituted oxindoles and thioamides.

During several past years, we have developed [1–2] a novel synthetic approach toward (*Z*)-3-[amino(phenyl/methyl)methylidene]-1,3-dihydro-2*H*-indol-2-ones and have demonstrated [3–4] its application potential in the synthesis of various tyrosine kinase inhibitors, including hesperadin and the approved drug nintedanib. The key step of the synthesis involves the Eschenmoser coupling reaction (ECR) between a substituted 3-bromooxindole **1** and appropriate primary, secondary or tertiary thioamides which proceedes smoothly in a polar aprotic solvent (DMF or MeCN) and mostly without any base or thiophilic agent (Scheme 1).

Such an arrangement is not typical, since at least the presence of a base is traditionally necessary for the successful performance of the ECR. Moreover, α-halolactams (as, e.g., 3-bromoxindole) have not been used in ECR before, only α-haloketones and α-haloesters. Finally, the use of primary thioamides (R^3^, R^4^: H) in ECR was quite unique – there are only two other examples [5–6] described in the literature starting from thioacetamide or 3-phenylpropanethioamide. In existing literature sources [7–9], primary thioamides were ineffective as reaction components in ECR as they can easily transform into nitriles under the reaction conditions typical for ECR.

**Scheme 2:**
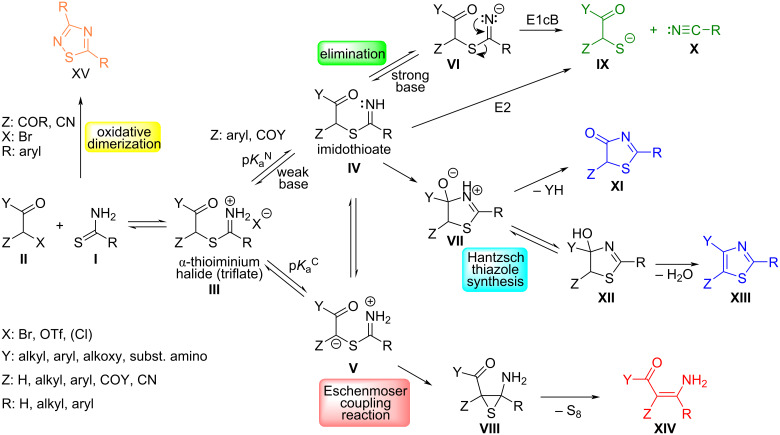
Possible reactions of α-haloketones, esters and amides with primary thioamides.

In fact, there are even three different reaction pathways (Scheme 2) that can compete with the ECR when primary thioamides **I** react with a α-haloketone or α-haloester **II**. The initially formed α-thioiminium salt **III** can undergo either a base-catalyzed elimination to give nitrile **X** and thiol **IX** [10–12] or cyclization to give a thiazole **XIII** or thiazolone **XI** depending on the substituent at the carbonyl group Y.

Both side reactions involve the imidothioate **IV** formed via deprotonation from nitrogen (p*K*_a_^N^ in Scheme 2). The imidothioate **IV** can undergo cyclization to give an energetically favorable five-membered thiazoline ring **VII** which then either eliminates a leaving group Y^−^ (when Y: alkoxy, amino) or a water molecule (when Y: alkyl, aryl). This reaction pathway represents the well-known Hantzsch thiazole synthesis [13–21]. If the imidothioate **IV** is further deprotonated [10–12] at nitrogen using a strong base (e.g., NaOH/H_2_O or EtONa/DMF are strong enough), then another intramolecular reaction pathway opens that involves either the *E*2 or *E*1cB-like mechanism to give the corresponding nitrile **X** and thiolate **IX**, which can be further alkylated with an excess of α-halogen component to give a symmetrical sulfide.

Only in very few cases when the starting α-thioiminium salt **III** contains an appropriate acidifying group Z (Z: CN, COR, COOR) then a proton cleavage from the α-carbon (p*K*_a_^C^ in Scheme 2) can occur (**V**) with a subsequent carbanion attack to the neighboring iminium group to form a three-membered thiirane ring (**VIII**). The thiirane then spontaneously decomposes into an ECR product (**XIV**) without any external thiophile [1–2,5–6]. However, the acidifying effect of the Z-group appears to be not the only prerequisite for successful ECR because in most cases 2-halomalonates [16–19] and 2-chloroacetoacetates [20] give the corresponding thiazoles and thiazolones, respectively (yields 70–95%). The presence of a base and the type of solvent seems to be an important factor for the reaction course. In toluene, ionic liquid or in refluxing ethanol without a base [16,19–20] or in the presence of weakly basic pyridine [17–18,21] (p*K*_a_ = 5.23 in water, 3.4 in DMSO, 3.3 in DMF, and 5.44 in MeOH) [22] the formation of thiazole/thiazol-4-one (**XI**/**XIII**) is clearly preferred, whereas reactions in common chlorinated solvents (CH_2_Cl_2_, CHCl_3_) containing either an equivalent of strong base (methoxide) or excess of medium base in heterogeneous system (e.g., carbonate with p*K*_a_ = 9.93 in water) tend to the formation [5–6] of the ECR product (**XIV**). A similar tendency to produce either ECR or thiazole products depending on the solvent was observed already by Eschenmoser and his co-workers for the reaction of α-bromoketones with thiolactams [23]. On the other hand, Bergman et al. recommended [24] a polar aprotic solvent such as DMSO that facilitates the formation of both α-thioiminium salt (**III**) as well as Eschenmoser contraction involving the attack of the exposed carbanion towards the imine/iminium group (**V**). Based on our previous experience [3–4] we favor dimethylformamide (DMF) as a polar aprotic solvent that can be easily distilled-off after the ECR under reduced pressure.

The last complication with aromatic primary thioamides concerns their easy oxidation with electron-poor α-haloesters (acetoacetates and α-cyanoacetates). As early as in 1976 Potts and Marshall noticed [25] that ethyl 2-bromo-2'-nitrobenzoylacetate reacts with thiobenzamide to give not only the expected 4-(2-nitrophenyl)-2-phenylthiazole-5-carboxylate, but also the dimerization product of thiobenzamide – i.e*.,* 3,5-diphenyl-1,2,4-thiadiazole (**XV**). Similar observations have been made with other thiobenzamides [26–27]. The latter authors concluded that the relative occurrence of 1,2,4-thiadiazoles (**XV**) during the Hantzsch reaction probably depends on the steric demands of the starting α-haloketones/esters **II**, but the real explanation is probably more complicated.

**Figure 1:**
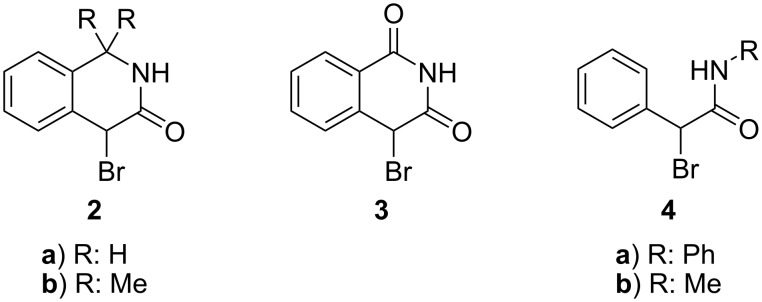
Studied α-bromoamides and α-bromolactams.

Therefore, we have decided to examine the possible extension of our approach involving primary thioamides to other structurally similar α-bromolactams **2** and **3** and α-bromoamides **4** (Figure 1) and to find structure–reactivity and medium–reactivity relationships that govern ECR in those cases.

## Results

First, we tried to prepare 4-bromo-1,4-dihydroisoquinolin-3(2*H*)-one (**2a**) that represents the homoanalogue of the parent 3-bromoxindole (**1**; R^1^, R^5^: H). The starting 1,4-dihydroisoquinolin-3(2*H*)-one was prepared from commercially available 2-indanone by Schmidt rearrangement with azoimide [28]. Unfortunately, its bromination using various agents (NBS, dioxane-Br_2_ complex, CuBr_2_) under various conditions always failed or led to oxidative aromatization of the lactam ring – i.e., to 3-hydroxyisoquinoline. Therefore, we decided to prepare [29] an analogous 1,1-dimethyl derivative **2b** whose methyl groups (R: Me) prevent such aromatization.

**Scheme 3:**
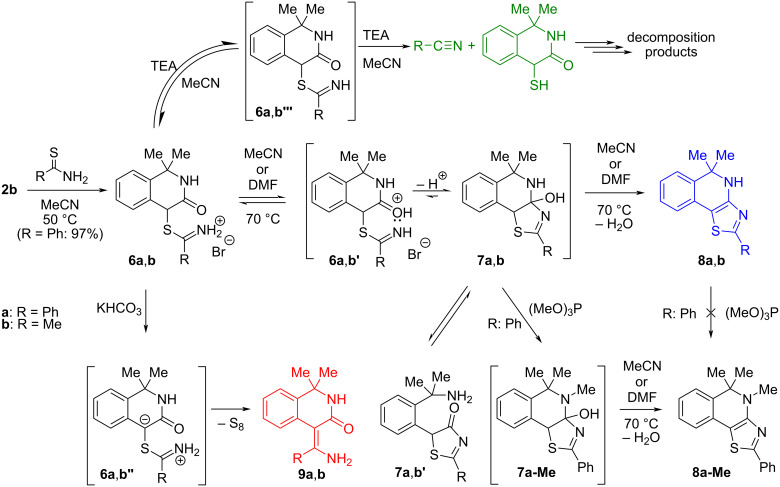
Reaction of 4-bromo-1,1-dimethyl-1,4-dihydroisoquinolin-3(2*H*)-one (**2b**) with thiobenzamide and thioacetamide.

The reaction of **2b** with thiobenzamide was studied under different reaction conditions. In acetonitrile, the corresponding white α-thioiminium salt **6a** crystallized from the hot solution after 30 min in a yield of 97%. As the salt is insoluble in common deuterated solvents (CDCl_3_, CD_3_CN) or quickly decomposes in their solutions (DMSO-*d*_6_, MeOD-*d*_4_, D_2_O), it was impossible to measure its NMR spectra and the only characterization involves MALDI–MS, IR, and melting point. The salt **6a** was then treated in various solvents with or without additive (thiophile, base/acid) to give diverse products of cyclization (**8a** or **8a-Me**), ECR (**9a**) or decomposition (Scheme 3 and Table 1).

**Table 1:** Reaction of salt **6a** under different reaction conditions.

Entry	Solvent	Thiophile (equivalents)	Additive (equivalents)	Temp. (°C)	Yield of **8a**/**8a**-Me (%)	Yield of **9a** (%)	Other products (%)

**1**	DMF	–	–	25	74/–	–	–
**2**	DMF	–	TEA (1)	25	–/–	–	–^a^
**3**	DMF	–	KHCO_3_ (3)	25	–/–	62	18^b^
**4**	MeCN	–	–	70	72/–	–	–
**5**	MeCN	Ph_3_P (1.5)	–	70	34/–	–	–
**6**	MeCN	–	TEA (1)	25	–/–	–	32^c^
**7**	MeCN	–	KHCO_3_ (3)	25	–/–	55	26^b^+18^c^
**8**	(MeO)_3_P (neat)		–	70	36/16	–	–
**9**	(MeO)_3_P (neat)		TFA (0.8)	70	61/23	–	–
**10**	(MeO)_3_P (neat)		TEA (0.7)	70	27/18	–	–
**11**	CHCl_3_	–	KHCO_3_ (3)	55	60–80/–	20–40	–
**12**	CHCl_3_	–	TEA (1)	55	–	–	36^c^
**13**	DCM	–	KHCO_3_ (3)	25	–	15–60	15–25^a^
**14**	DCM		TEA (1)	25	26/0	27	–

^a^Unidentified decomposition products; ^b^thiobenzamide; ^c^benzonitrile.

Table 1 shows that in both polar aprotic solvents without base and thiophile (entries 1 and 4) only tricyclic 5,5-dimethyl-2-phenyl-4,5-dihydrothiazolo[4,5-*c*]isoquinoline (**8a**) was formed probably through unstable intermediate (**7a**). Compound **7a** can be detected by ESI–MS analysis of the reaction mixture as it has a different fragmentation pattern (easily loses water – see Supporting Information File 1, Figure S1) than the starting isobaric salt **6a** (loses benzonitrile and thiobenzamide). However, all attempts to isolate intermediate **7a** have always failed. The addition of a strong thiophile (Ph_3_P; Table 1, entry 5) does not turn the reaction toward the ECR, and only several other decomposition products can be detected together with **8a**. Triethylamine causes the decomposition of salt **6a** in both polar aprotic solvents (Table 1, entries 2 and 6). In MeCN where TEA behaves as a stronger base, the presence of benzonitrile was proved in the ^1^H NMR spectrum of the crude reaction mixture.

On the other hand, the addition of 3 equivalents of a mild base (solid KHCO_3_; p*K*_a_ = 6.35 in water [30]) into the DMF solution or MeCN suspension of salt **6a** leads to the desirable ECR product **9a** in 62% or 55% yield (entries 3 and 7 in Table 1) together with other decomposition products from which only thiobenzamide and benzonitrile were identified.

In pure trimethyl phosphite (Table 1, entry 8) that acts simultaneously as polar solvent and mild thiophile, the parallel formation of products **8a** and **8a-Me** was observed, but their combined yield was lower than in DMF or MeCN. The introduction of the *N*-methyl group coming from trimethyl phosphite must occur prior to the dehydration step of the intermediary thiazole **7a** because heating of independently prepared **8a** with trimethyl phosphite does not give **8a-Me** at all. The combined yield of **8a** and **8a-Me** can be increased when trifluoroacetic acid is added (Table 1, entry 9). Its role probably involves an acid-catalyzed elimination of a water molecule from **7a** or **7a-Me**. On the other hand, the addition of a stronger base (triethylamine; entry 10 in Table 1) only decreased the combined yield of **8a** and **8a-Me**, but no ECR product **9a** was detected.

The change of polar aprotic to chlorinated solvents (DCM, CHCl_3_) as recommended by Eschenmoser [23] leads to ambiguous results. In amylene-stabilized CHCl_3_ at 55 °C and with 3 equivalents of solid KHCO_3_ (Table 1, entry 11) the cyclization product **8a** is dominant, but the ECR product **9a** is also isolable from the reaction mixture in moderate yield. Unfortunately, the reaction carried out in such a heterogeneous system is not fully reproducible with respect to product distribution. The change of the base from solid KHCO_3_ to TEA (Table 1, entry 12) causes the decomposition of salt **6a** into a complex mixture of products from which only benzonitrile was unambiguously identified in the ^1^H NMR spectrum of the crude reaction mixture. Another product is probably 4-sulfanyl-1,4-dihydroisoquinolin-3(2*H*)-one, whose isolation in the pure state failed. Comparable results were obtained in DCM at 25 °C with KHCO_3_ (Table 1, entry 13) but with TEA (Table 1, entry 14) only moderate yields of **8a** and **9a** were obtained.

To generalize the results obtained with thiobenzamide, the reaction of **2b** with thioacetamide was also briefly investigated, but the corresponding salt **6b** was not isolated. The reaction in DMF without any additive (16 h, rt) also led to tricyclic 5,5-dimethyl-2-methyl-4,5-dihydrothiazolo[4,5-*c*]isoquinoline (**8b**) but its isolated yield was only 40% in addition to thioacetamide (19%) and unidentified decomposition byproducts. In the case when the reaction was base-catalyzed (DMF, 3 equiv of KHCO_3_, 48 h), the corresponding ECR product **9b** was isolated (48%) together with the starting thioacetamide (21%) and additional unidentified byproducts.

**Scheme 4:**
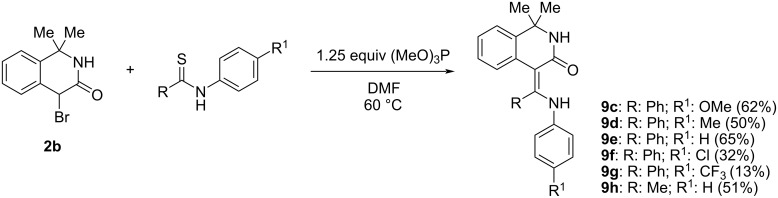
Reaction of 4-bromo-1,1-dimethyl-1,4-dihydroisoquinolin-3(2*H*)-one (**2b**) with 4’-substituted thiobenzanilides and thioacetanilide.

If secondary thioamides (thioacetanilide, *N*-(4-R-phenyl)thiobenzamide) were used instead of primary thiobenzamide or thioacetamide, then the expected ECR only occurred in DMF (Scheme 4) and the corresponding aminomethylidene-1,4-dihydroisoquinolin-3(2*H*)-ones (**9c–h**) were isolated under optimized reaction conditions in varying yields (13–65%) depending mainly on substitution of the *N*-phenyl ring (Scheme 4). Table 2 shows that the optimum conditions involve slightly elevated temperature, absence of base, and the presence of a mild thiophile. On the other hand, higher temperatures, stronger bases and thiophiles decrease the reaction yield, or even cause complete decomposition of the starting materials.

**Table 2:** Reaction of salt **2b** with thiobenzanilide in DMF under different reaction conditions.

Entry	Temperature (°C)	Thiophile (equivalents)	Base (equivalents)	Yield of **9e** (%)

**1**	25	–	–	17
**2**	60	–	–	32
**3**	100	–	–	0^a^
**4**	60	Ph_3_P (1)	–	0^a^
**5**	60	–	Et_3_N (1)	5
**6**	25	–	KHCO_3_ (3)	21
**7**	60	(MeO)_3_P (1.25)	–	65

^a^Decomposition.

The ECR was also performed under optimized conditions with other substituted secondary thioamides. While the parent thioacetanilide, thiobenzanilide and thiobenzanilides containing electron-donating groups gave the products in isolated yields higher than 50%, the presence of an electron-withdrawing group resulted in a substantial decrease. We previously [3] observed the same trend when 3-bromooxindole (**1**) was reacted with thiobenzanilides.

Finally, tertiary *N*,*N*-dimethylthiobenzamide was reacted with **2b** but only formation of the corresponding thioiminium salt was observed in both MeCN and DMF which was in equilibrium with the starting compounds in a ratio of approximately 1:2. The addition of a base or any thiophile always caused only the decomposition to a complex mixture of products in which no ECR product was detected.

**Scheme 5:**
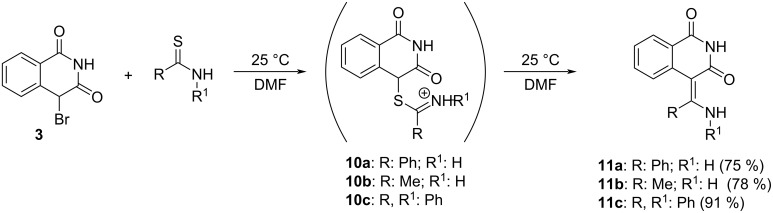
Reaction of 4-bromoisoquinoline-1,3(2*H*,4*H*)-dione (**3**) with thiobenzamide, thioacetamide, and thiobenzanilide.

Next, we performed modification of the lactam **2** structure involving the replacement of a quaternary carbon carrying two electron-donating methyl groups with an electron-withdrawing carbonyl group (**2b** → **3**). The starting isoquinoline-1,3(2*H*,4*H*)-dione was prepared [31] from homophthalic acid and then brominated with NBS to give 4-bromoisoquinoline-1,3(2*H*,4*H*)-dione (**3**) with a yield of 65%. Its reaction with thiobenzamide and thioacetamide in DMF was carried out without isolation of the intermediary thioiminium salts **10a** and **10b** and only gave the ECR products **11a**,**b** (Scheme 5) in good yield (75 and 78%, respectively). No thiazole (cf. reactions of compound **2b**) formation was observed. An even better yield of ECR (**11c**, 91%) was achieved with thiobenzanilide, again without isolation of the intermediary salt **10c**. The presence of any base or thiophile had no positive effect on the course of the reaction.

**Scheme 6:**
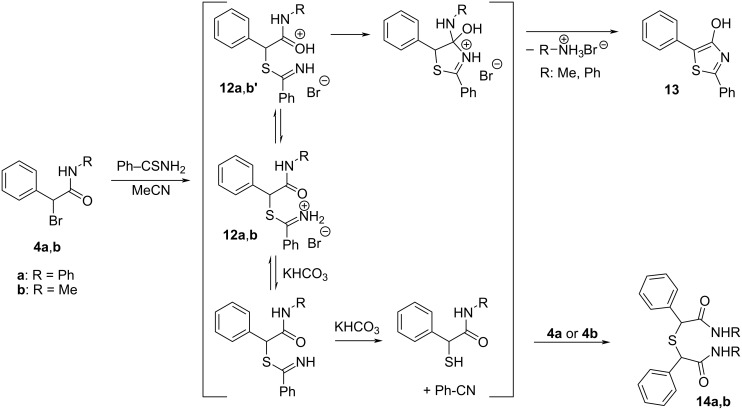
Reaction of *N*-phenyl- and *N*-methyl-2-bromo(phenyl)acetamide (**4a**,**b**) with thiobenzamide in acetonitrile.

Simplification of a structure of lactams **1**, **2b**, and **3** (i.e., cleavage of Ar–N bond or removal of >C(CH_3_)_2_ or >C=O bridge) leads to secondary α-bromo(phenyl)acetamides **4a** and **4b**. When α-bromoamide **4a** was treated with thiobenzamide without any base (entries 1 and 7 in Table 3), 2,5-diphenyl-1,3-thiazol-4-ol (**13**) was the only product (Scheme 6). This means that the cleavage of the amide group that evolves aniline (p*K*_a_ = 4.6) occurred smoothly after the initial thiazole ring closure (through **12a’** in Scheme 6). The addition of a thiophile (trimethyl phosphite) does not turn the reaction toward ECR and only decreases the yield of **13** (Table 3, entries 4–6). The addition of a weak base (KHCO_3_, entries 2 and 8 in Table 3) causes partial elimination of salt **12a** to *N*-phenyl-2-sulfanyl(phenyl)acetamide whose reaction with **4a** gives stable sulfide **14a**, together with a minor occurrence of thiazole **13** and thiobenzamide. A somewhat different behavior was observed with substrate **4b**, where a mixture of thiazole **13**, sulfide **14b** and thiobenzamide was formed in DMF (Table 3, entries 9 and 10) even without any base. This behavior corresponds to a much worse leaving ability of methylamine (p*K*_a_ = 10.6) from cyclic intermediate en route to thiazole **13**. Any attempted ECR starting from **4a**,**b** and thioacetamide and thiobenzanilide failed regardless of the conditions adopted. Complex mixtures of the decomposition products were not separated.

**Table 3:** Reaction of α-bromoamides **4a–c** with thiobenzamide in acetonitrile under different reaction conditions.

Entry	Solvent	Starting α-bromoamide	Temperature (°C)	Thiophile (equivalents)	Base (equivalents)	Yield of **13** (%)^a^

**1**	MeCN	**4a**	25	–	–	93
**2**	MeCN	**4a**	25	–	KHCO_3_ (3)	16^b^
**3**	MeCN	**4a**	25	–	TEA (1)	decomp.
**4**	MeCN	**4a**	25	(MeO)_3_P (1)	–	53
**5**	MeCN	**4a**	60	(MeO)_3_P (1)	–	89
**6**	MeCN	**4a**	25	(MeO)_3_P (1)	NMM^c^ (15%)	44
**7**	DMF	**4a**	25	–	–	91
**8**	DMF	**4a**	25	–	KHCO_3_ (3)	26^d^
**9**	DMF	**4b**	25	–	–	45^e^
**10**	DMF	**4b**	80	–	–	84^f^

^a^All yields were calculated from ^1^H NMR spectra of the crude reaction mixture (see Supporting Information File 1); ^b^thiobenzamide (52%) and sulfide **15a** (32%); ^c^NMM: *N*-methylmorpholine; ^d^thiobenzamide (44%) and sulfide **15a** (30%); ^e^thiobenzamide (30%) and sulfide **15b** (23%); ^f^thiobenzamide (9%) and sulfide **15b** (7%).

## Discussion

All observed reactions involve α-thioiminium salts (**6a**,**b**, **10a**,**b**, **12a**,**b**, **15**) whose formation is easy in a polar aprotic solvent. Acetonitrile is the best solvent for the reaction if the intermediary salt (e.g., **6a**) may be isolated, since it is only sparingly soluble in it. Since the p*K*_a_ values of known primary aliphatic/aromatic α-thioiminium salts (N–H acidity) in water [10,32–33] are less than 7 and 6, respectively, already the medium base (triethylamine with p*K*_a_ > 9) [22] quantitatively generates free imidothioate in all solvents. The change of water to polar aprotic solvents such as DMF should have only a negligible influence on the α-thioiminium salt p*K*_a_ value as in the case of the structurally related ammonium salts [22].

**Scheme 7:**
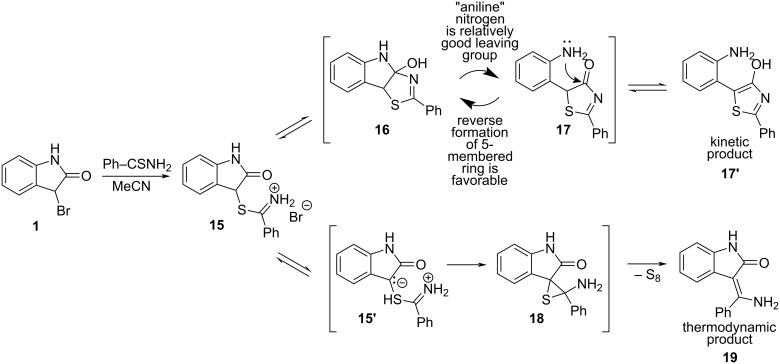
Transformation of salt **15** under kinetic and thermodynamic control conditions [1].

All experiments with salts **6a**,**b** derived from **2b** and thiobenzamide or thioacetamide showed that three of four possible products (thiazoles **8a**,**b**, ECR products **9a**,**b** and nitriles; cf. Scheme 3) are formed depending on the reaction conditions specified in Table 1 and Supporting Information File 1. Only oxidation of thiobenzamide to 3,5-diphenyl-1,2,4-thiadiazole was never observed. Triethylamine (p*K*_a_ = 10.67 in water, 9.25 in DMF, and 18.5 in MeCN) [22] causes the decomposition of imidothioates **6a**,**b'''** in both polar aprotic solvents (DMF and MeCN) through the elimination route. For structurally related *S*-ethyl ethanimidothioate and *S*-ethyl benzimidothioate in aqueous solution, such reaction pathway begins to open [33] at pH > 6 and 8, respectively. The subsequent formation of imidothioates **6a**,**b'''** and their base-catalyzed elimination (Scheme 3) takes place easily, especially in MeCN where triethylamine behaves as a much stronger base. A much weaker base – solid KHCO_3_ – is probably still strong enough to generate free imidothioates **6a**,**b'''** which undergo parallel base-catalyzed elimination (minor route to nitriles) and intramolecular proton-transfer between the carbon and nitrogen to give the zwitterionic intermediates **6a**,**b''** necessary for successful ECR. On the other hand, without any base only thiazoles **7a**,**b** are formed probably from isomeric forms **6a**,**b'** of the starting salts **6a**,**b**. In contrast to the tricyclic intermediate **16** formed from oxindole α-thioiminium salt (**15**), which in MeCN undergoes a ring transformation [1–2] to give 2-phenyl-5-(2-aminophenyl)-4-hydroxy-1,3-thiazole (**17'**) (Scheme 7) under kinetic control, the analogous intermediary hydroxythiazole **7a** (Scheme 3) does not decompose into analogous thiazole **7a'** due to much worse nucleofugality of the leaving amine group.

The leaving abilities of "aniline" nitrogen (anilinium has a p*K*_a_ = 4.60 in water) [22] in intermediate **16** (Scheme 7) and "phenethylamine" nitrogen (2-phenyl-2-propylammonium has a p*K*_a_ = 10.38 in water) [34] in **7a** (Scheme 3) must differ by several orders of magnitude, which makes the irreversible elimination of the water molecule from **7a**,**b** to give **8a**,**b** to be preferred over the ring opening giving **7a**,**b'**. The worse leaving ability of aliphatic amine nitrogen is also manifested in a much lower yield of thiazole **13** from salt **12b** which preferably undergoes elimination, while salt **12a** having a much better leaving aniline moiety, prefers cyclization to give **13** (Scheme 6).

Another key factor for successful ECR concerns the acidity of C–H in particular α-thioiminium salts **6a**,**b**, **10a**,**b**, **12a**, **15** (p*K*_a_^C^) or the ease of proton transfer between the carbon and nitrogen in imidothioate followed by formation of the thiirane ring. Since direct measurements of acidity at carbon (p*K*_a_^C^) and the rate of ring closure is impossible, we calculated the energies of important reaction species on the potential energy surface for imidothioates derived from α-thioiminium salts **6a**, **10a**, **12a**, and **15** at the B3LYP-D3/6-311+G(d,p) level of theory.

**Figure 2:**
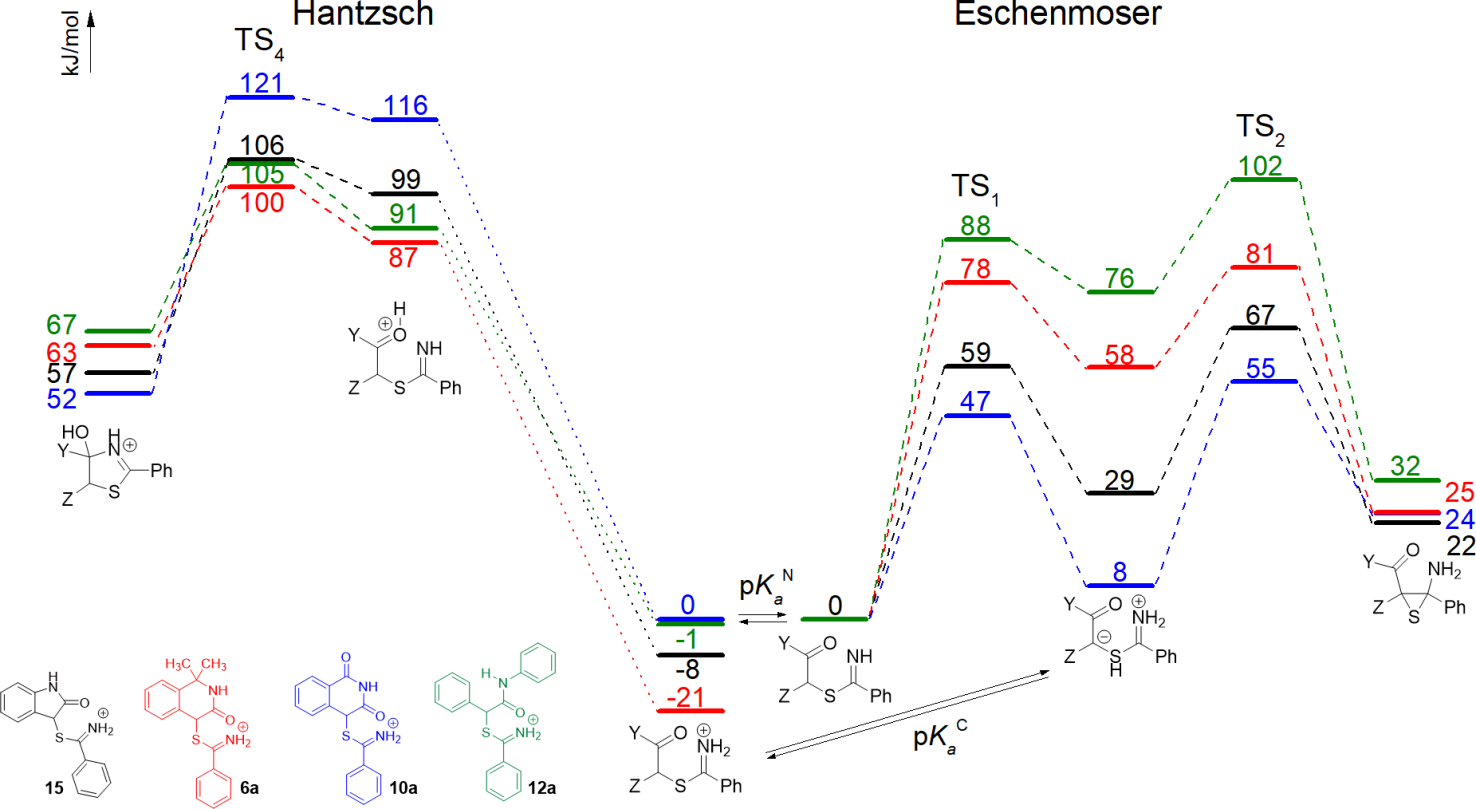
Comparison of energy profiles (relative Gibbs energies at 298 K in kJ·mol^−1^ for the ECR (right) and Hantzsch (left) pathways for salts **6a**, **10a**, **12a**, and **15** calculated at the B3LYP-D3/6-311++G(d,p) level of theory in *N*,*N*-dimethylformamide (SMD). Extended version of Figure 2 involving energies of final products can be found in Supporting Information File 1.

Quantum calculations (Figure 2) have shown that the reaction pathway that involves an intramolecular proton transfer between the α-carbon and imine nitrogen through TS_1_ is much more favorable for imidothioate derived from 4-bromoisoquinoline-1,3(2*H*,4*H*)-dione (**3**) and 3-bromooxindole (**1**) with activation free energies 47 and 59 kJ·mol^−1^ than from 4-bromo-1,1-dimethyl-1,4-dihydroisoquinolin-3(2*H*)-one (**2b**) and *N*-phenyl-2-bromo(phenyl)acetamide (**4a**) with activation free energies 78 and 88 kJ·mol^−1^. These trends fully correspond to a combination of electronic effects (bridging C=O has an acidifying effect as compared to C(CH_3_)_2_) and the effect of ring strain [35–36] (C–H in the 5-membered α-thioiminium salt **15** is more acidic than in the 6-membered salts **6a**,**b** or non-cyclic salts **12b**).

The subsequent formation of the thiirane ring through TS_2_ (which is strongly entropically favored although the ring strain involved in the enthalpy term disfavors it) shows the opposite trend. This means that the activation free energies are decreasing from 47 to 23 kJ·mol^−1^, respectively. Spontaneous co-catenation (or thiophile-assisted when a P(III) compound is added) extrusion of sulfur from thiirane [13] then gives the final ECR product.

The second reaction pathway giving Hantzsch-type products occurs when no base is present in the reaction mixture. It means that the reaction starts directly from α-thioiminium salts (**6a**, **10a**, **12a**, **15**). The reaction involves a proton transfer between the iminium nitrogen and carbonyl oxygen, generating both nucleophilic and electrophilic centers, i.e., a free amino group and protonated carbonyl group, which is much more prone to nucleophilic attack than a carbonyl group itself. Quantum calculations (see the left side of Figure 2) show that the relative stabilities of these isomeric salts have the highest contributions to the overall barrier for Hantzsch reaction pathways because their cyclization through TS_4_ is very fast (activation free energies 5–15 kJ·mol^−1^). The overall (relative) energy barriers now decrease in the order **6a** > **10a** > **15** > **12a**, which is virtually opposite to the order of ECR reactivity. Thus, the substrates with the lowest barriers for ECR couplings have the highest energies for the Hantzsch reaction and vice versa.

The calculated reaction profiles show that there are two factors that influence whether the reaction gives ECR or Hantzsch products. Except the above-mentioned different reaction barriers, it is the energy difference between the protonated α-thioiminium salt and its free base (imidothioate). The p*K*_a_^N^ values (N–H acidity) strongly influence the actual concentrations of α-thioiminium salts and the corresponding imidothioates in each reaction medium. Unfortunately, the p*K*_a_^N^ values of individual thioiminium salts **6a**, **10a**, **12a**, and **15** are not available experimentally, and their exact determination using calculations is also not completely accurate, especially because of the uncertainty of the energy of a solvated proton. However, it can be expected that with the help of calculations we are able to reproduce well the differences in the relative energies of the protonated and deprotonated forms related to the most acidic salt **10a** as standard. It means that we can estimate the minimum difference (vertical shift) between energies and determine the order of N–H acidity of individual salts **10a** > **12a** > **15** > **6a**. (Table 4).

**Table 4:** Calculated energy difference between the α-thioiminium salts and its free base (imidothioates) in absolute values as well as in relative values related to the most acidic salt **10a** (numbers in parentheses).

Salt	Δ*G*_thioiminium_ − (Δ*G*_imidothioate_ + Δ*G*_H_+) (kJ·mol^−1^)

**6a**	595 (−21)
**10a**	574 (0)
**12a**	575 (−1)
**15**	582 (−8)

It is obvious that a higher acidity leads to a greater occurrence of the neutral form (imidothioate) in the given reaction environment and vice versa. Therefore, a higher acidity favors the ECR over the Hantzsch pathway. This, together with the height of the activation barriers for individual pathways and intermediates, explains the observed formation of different types of products. While salt **10a** with a low barrier value for the ECR pathway and the highest CH acidity provides exclusively the ECR coupling product **11a**, the α-thioiminium salt **12a** with a large barrier for the ECR pathway and weaker acidity reacts exclusively in the sense of the Hantszch cyclization pathway. Other substrates move between these two extremes, and the ratio of the resulting products is influenced by external factors such as the amount and strength of the external base.

## Conclusion

Our study corroborates the complexity of the reaction between α-bromolactams/α-bromoamides and primary thioamides represented by thiobenzamide and thioacetamide. The appearance and significance of individual intermediates which are decisive for the formation of individual products is strongly dependent on the reaction conditions and the structure of the starting α-bromolactams/α-bromoamides. Both p*K*_a_^N^ and p*K*_a_^C^ values of the starting α-thioiminium salt appear to be decisive factors regardless of the solvent used for the reaction. For a smooth ECR starting from primary thioamides, it is therefore highly beneficial to generate free imidothioates but without the excess of a base. The suitable base must be strong enough to generate the free imidothioate (p*K*_a_^base^ ≈ p*K*_a_^N^) but still too weak for the (undesired) consecutive elimination giving nitriles. Experiments as well as quantum calculations have shown that the acidifying effect of a solitary phenyl group (Z = Ph in Scheme 2 and in Figure 2) is not sufficient to enable a successful ECR. On the other hand, if the free rotation of phenyl group (Z) is restricted by its further bonding through a group Y then its acidifying effect steeply grows and ECR becomes possible. The thiophilic agent does not have a positive influence on the ECR in those cases which indicates that the cleavage of the intermediary thiirane ring should occur after the rate-limiting step of the ECR sequence.

When no base is present, then the Hantzsch cyclization pathway can occur, and the structure of the product depends on the leaving ability of the lactam/amide nitrogen. A good leaving “aniline” group prefers C–N cleavage, whereas the worse leaving “phenethylamine” prefers water elimination.

## Experimental

The starting 1,1-dimethyl-1,4-dihydroisoquinolin-3(2*H*)-one and isoquinoline-1,3(2*H*,4*H*)-dione were prepared using adapted procedures described in the literature [29–30,37] and their subsequent selective monobromination to **2b** or **3** was achieved with NBS in chloroform under MCPBA initiation (see Supporting Information File 1). α-Bromophenylacetic acid amides (**4a**,**b**) were prepared from 2-bromo-2-phenylacetyl chloride [38] and the corresponding amine in DCM or toluene at reduced temperature (see Supporting Information File 1).

Thiobenzamides and thiobenzanilides were prepared by magnesium chloride-catalyzed thiolysis of commercially available benzonitriles [39] or by thionation of the corresponding N-substituted amides [40] using pyridine–P_4_S_10_ as sulfurization agent. Other chemicals and solvents were purchased from Acros Organics, Sigma-Aldrich, and Fluorochem and were used as received.

^1^H and ^13^C (APT) NMR spectra were recorded on a Bruker Avance III 400 MHz or on a Bruker Ascend 500 MHz instrument. Chemical shifts (δ) are referenced to TMS (δ = 0) or solvent residual peaks δ(CDCl_3_) = 7.24 ppm (^1^H) and 77.0 ppm (^13^C), δ(DMSO-*d*_6_) = 2.50 ppm (^1^H) and 39.6 ppm (^13^C). High-resolution mass spectra were recorded on a MALDI LTQ Orbitrap XL equipped with nitrogen UV laser (337 nm, 60 Hz, 8–20 μJ) in positive ion mode. For the CID experiment using the linear trap quadrupole (LTQ) helium was used as the collision gas and 2,5-dihydroxybenzoic acid (DHB) or (2-methylprop-2-en-1-yliden)malononitrile (DCTB) as the MALDI matrix. Elemental analyses were performed on a Flash 2000 Organic Elemental Analyser (Thermofisher). For samples containing chlorine, mercurimetric titration was used. IR spectra were recorded on a Nicolet iS50 equipped with an ATR diamond crystal (neat solid samples). Flash chromatography was performed using a Büchi Reveleris^®^ X2 flash chromatography system equipped with a UV–vis/ELSD detector and Reveleris Flash pure cartridges (12–40 g, 35–45 μm, 53–80 Å) or puriFlash^®^ Alumine N 32/63 µm cartridges (12 g).

The reaction pathways were calculated [41] with the Gaussian 16 rev. C.01 program, using the B3LYP functional with D3 Grimme’s dispersion correction [42–43] and 6-311++G(d,p) basis set. All reported minima and transition state structures were confirmed by calculation and diagonalization of their Hessian matrices. The reported energies are Gibbs free energies calculated with the SMD method in *N*,*N*-dimethylformamide solvent [44] at 298.15 K and 1 bar standard state.

## Supporting Information

File 1 Experimental part.
