# Supplementary material for: Eschenmoser coupling reactions starting from primary thioamides. When do they work and when not?
Source: Beilstein J Org Chem. 2023 Jun 9;19:808–19. doi: 10.3762/bjoc.19.61 (PMC10280059; doi:10.3762/bjoc.19.61)
Supplement: File 1 — Experimental part. [file Beilstein_J_Org_Chem-19-808-s001.pdf]

BEILSTEIN JOURNAL OF ORGANIC CHEMISTRY

Supporting Information

for

Eschenmoser coupling reactions starting from primary thioamides. When do they work and when not?

Lukáš Marek, Jiří Váňa, Jan Svoboda and Jiří Hanusek

Beilstein J. Org. Chem. **2023,** *19,* 808–819. doi:10.3762/bjoc.19.61

Experimental part

License and Terms: This is a supporting information file under the terms of the Creative Commons Attribution License (https://creativecommons.org/licenses/by/4.0). Please note that the reuse, redistribution and reproduction in particular requires that the author(s) and source are credited and that individual graphics may be subject to special legal provisions.
The license is subject to the *Beilstein Journal of Organic Chemistry* terms and conditions: (https://www.beilstein-journals.org/bjoc/terms)

Table of contents

Synthesis and characterization of **2b**, **3**, **4a**,**b**, **6a**, **8a**,**b**, **8a-Me**, **9a**–**h**, **11a**–**c**, **14a**,**b** S2

Figure S1 - ESI-MS CID of compounds **6a** and **7a** S12

^{1}H and ^{13}C NMR spectra of **2b**, **3**, **4a**,**b**, **6a**, **8a**,**b**, **8a-Me**, **9a**–**h**, **11a**–**c**, **14a**,**b** S13

HMBC and HSQC NMR spectra of **8a-Me** S34

MALDI-HRMS spectra of **2b**, **3**, **4a**,**b**, **6a**, **8a**,**b**, **8a-Me**, **9a**–**h**, **11a**–**c**, **14a**,**b** S35

IR spectrum of **6a** S40

Quantum chemical coordinates for compounds S41

References S95

Synthesis of 4-bromo-1,1-dimethyl-1,4-dihydroisoquinoline-3(2*H*)-one (2b)

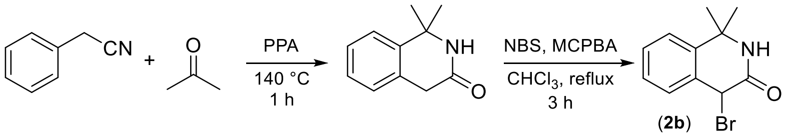

Polyphosphoric acid (450 g) was heated [1] to 135 °C in a 1-L-three-necked round-bottomed flask equipped with a reflux condenser and phenylacetonitrile (60 g, 0.51 mol) was added during 3 min under stirring. After 10 min, acetone (80 mL, 1.1 mol) was added dropwise during 1 hour and the reaction mixture was stirred at 140 °C for the next 1 hour. Then, another portion of acetone (15 mL) was added, the reaction mixture was stirred for 20 min, cooled, and poured into ice water (3 L). Extraction with dichloromethane (3 × 250 mL), washing of the combined organic layers with 5% aq. $NaHCO_3$ (2 × 250 mL), drying (anhydrous Na_2SO_4), and filtration of the organic phase through a plug of silica gel (60 g) gave a clear solution which was evaporated under reduced pressure. Yield 52 g (63%) of yellow solid 1,1-dimethyl-1,4-dihydroisoquinoline-3(2*H*)-one with m.p. 131-132 °C and ^1H NMR (500 MHz, $CDCl_3$) δ: 7.87 (bs, 1H, NH), 7.21 – 7.33 (m, 3H, Ar-H), 7.14 (d, *J* 7.2 Hz, 1H, Ar-H), 3.65 (s, 2H, CH_2), 1.60 (s, 6H, 2×CH_3) is in accord with ref. [2].

A solution of 1,1-dimethyl-1,4-dihydroisoquinoline-3(2*H*)-one (4.1 g, 23.4 mmol), *N*-bromosuccinimide (4.3 g, 24.3 mmol) and *m*-chlorobenzoic acid (0.6 g of 75% aq. solution) in CCl_4 (90 mL) was refluxed for 3.5 hours. The resulting yellow solution was washed with water (50 mL), 5% aq. $NaHCO_3$ (40 mL) and brine (50 mL) and dried with anhydrous Na_2SO_4. Evaporation of the solvent gave the crude product which was crystallized from hot acetonitrile/THF (2:1) with charcoal. Yield 3.9 g (66 %) of white crystalline **2b** with m.p. 206-207 °C. ^1H NMR (500 MHz, $CDCl_3$) δ: 8.01 (bs, 1H, NH), 7.36 – 7.43 (m, 2H, Ar-H), 7.29 – 7.36 (m, 2H, Ar-H), 5.52 (s, 1H, CH), 1.71 and 1.72 (2×s, 6H, 2×CH_3); ^{13}C NMR (125 MHz, $CDCl_3$) δ: 167.3 (C=O), 141.2 (C), 131.5 (C), 129.8 (CH), 129.5 (CH), 128.1 (CH), 124.6 (CH), 57.0 (C), 41.7 (CH), 31.4 (CH_3), 30.4 (CH_3); HRMS: calcd. for $C_{11}H_{13}BrNO$ $[M+H^+]$: calcd. 254.0175, found 254.0178; Elemental analysis: calcd. C: 51.99, H: 4.76, N: 5.51, Br: 31.44; found C: 52.33, H: 4.77, N: 5.30, Br: 31.10.

Synthesis of 4-bromoisoquinoline-1,3(2*H*,4*H*)-dione (3)

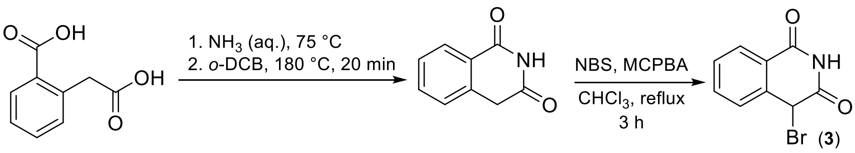

Homophthalic acid (10 g, 55.6 mmol) was mixed [3] with 25% aq. ammonia (15 mL, 0.2 mol), heated at 75 °C until complete dissolution, and then evaporated. The same operation was repeated once more with 25% aq. ammonia (10 mL, 0.13 mol). The resulting solid residue was suspended in *o*-chlorobenzene (25 mL) and heated at 175–180 °C for 20 min without a condenser. Then, the solvent was distilled off at 225 °C by bubbling of dry inert gas (nitrogen or argon) and the residue was dissolved in hot MeOH (30 mL). Cooling of the solution gave

6.8 g (71%) of isoquinoline-1,3(2*H*,4*H*)-dione as a white crystalline solid with mp. 237-238 °C and NMR spectra ^{1}H NMR (500 MHz, DMSO-d_6) δ (ppm): 11.30 (bs, 1H, NH), 8.01 (d, *J* 7.8 Hz, 1H, ArH), 7.64 (t, *J* 7.5 Hz, 1H, ArH), 7.45 (t, *J* 7.6 Hz, 1H, ArH), 7.37 (d, *J* 7.7 Hz, 1H, ArH), 4.03 (s, 2H, CH_2); ^{13}C NMR (125 MHz, DMSO-d_6) δ (ppm): 171.1 (C=O), 165.5 (C=O), 136.8 (C), 133.6 (CH), 128.0 (CH), 127.6 (CH), 127.3 (CH), 125.1 (C), 36.1 (CH_2) both in accordance with ref. [4].

The above prepared isoquinoline-1,3(2*H*,4*H*)-dione (0.8 g, 5 mmol) was suspended in amylene-stabilized chloroform (60 mL) and *N*-bromosuccinimide (0.9 g, 5 mmol) together with *m*-chloroperoxybenzoic acid (0.12 g of 75% aq. solution) were added. The reaction mixture was refluxed for 3 h and then evaporated under vacuum. The solid residue was quickly crystallized from boiling acetonitrile and THF (10 + 1 mL) to give 0.8 g (65%) of pale brown crystals of compound **3** with mp. 174-176 °C. ^{1}H NMR (500 MHz, $CDCl_3$) δ: 8.52 (bs, 1H, NH), 8.22 (d, *J* 7.8 Hz, 1H, Ar-H), 7.72 (t, *J* 7.6 Hz, 1H, Ar-H), 7.55-7.62 (m, 2H, Ar-H), 5.66 (s, 1H, CH); ^{13}C APT NMR (125 MHz, $CDCl_3$) δ (ppm): 167.6 (C=O), 163.1 (C=O), 136.5 (C), 134.8 (CH), 130.1 (CH), 129.5 (CH), 129.2 (CH), 123.8 (C), 38.1 (CH); HRMS: calcd. for $C_9H_7BrNO_2$ [M+H$^+$]: calcd. 239.9655, found 239.9658; Elemental analysis: calcd. C: 45.03, H: 2.52, N: 5.83; found C: 45.09, H: 2.53, N: 5.50.

α-Bromo(phenyl)acetamides (4a,b)

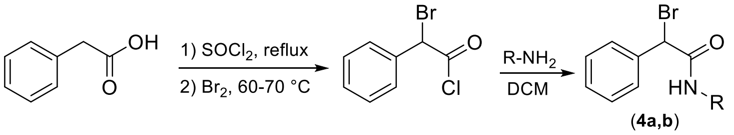

(**4a,b**)
a: R = Ph
b: R = Me

A mixture [5] of phenylacetic acid (22 g, 160 mmol) and thionyl chloride (60 mL, 818 mmol) was refluxed for 1.5 h and then cooled to 35 °C. Bromine (9.8 mL, 192 mmol) and aq. hydrobromic acid (56%) (10 drops) were added and the resulting solution was heated at 60 °C for 1 h and then for 2 h at 70 °C. Excess of thionyl chloride was distilled off under reduced pressure (300–400 torr) to give an oily residue which was purified by distillation using a short column (bp 135-136 °C at 18 torr). Yield 31.2 g (91 %) of yellowish liquid. ^{1}H NMR (400 MHz, $CDCl_3$) δ: 7.44-7.53 (m, 2H, Ar-H), 7.36-7.44 (m, 3H, Ar-H), 5.66 (s, 1H, CH).

The corresponding amine (10 mmol) was dissolved in a mixture of dry DCM (25 mL) with pyridine (1 mL, 12 mmol). After cooling to −40 °C a solution of α-bromo(phenyl)acetyl chloride (2.2 g, 10 mmol) in DCM (10 mL) was added dropwise and the reaction mixture was stirred at −40 °C for 30 min and then for 2.5 h at room temperature. The reaction mixture was diluted with DCM (30 mL) and washed with aq. HCl (5%, 40 mL), water (30 mL), aq. $NaHCO_3$ (5%, 30 mL) and brine (30 mL). The organic layer was dried with anhydrous Na_2SO_4 and evaporated to obtain amides **4a,b**.

N-Phenyl-2-bromo(phenyl)acetamide (4a)

White crystalline solid (1.9 g, 65 %); mp. 142 – 143 °C (ref. [6] gives 144 – 145 °C); ^{1}H NMR (500 MHz, $CDCl_3$) δ: 8.34 (bs, 1H, NH), 7.48-7.56 (m, 4H, Ar-H), 7.30-7.41 (m, 5H, Ar-H), 7.16 (t, *J* 7.4, 1H, Ar-H), 5.56 (s, 1H, CH).

N-Methyl-2-bromo(phenyl)acetamide (4b)

White crystalline solid crystallized from $CHCl_3$/n-hexane 1:2 (1.2 g, 52 %); mp. 94 – 96 °C (ref. [7] gives 99 – 100 °C); ^{1}H NMR (500 MHz, $CDCl_3$) δ: 7.41-7.46 (m, 2H, Ar-H), 7.30-7.40 (m, 3H, Ar-H), 6.70 (bs, 1H, NH), 2.90 (d, *J* 4.9, 3H, CH_3).

1,1-Dimethyl-3-oxo-1,2,3,4-tetrahydroisoquinolin-4-yl benzimidothioate hydrobromide (6a)

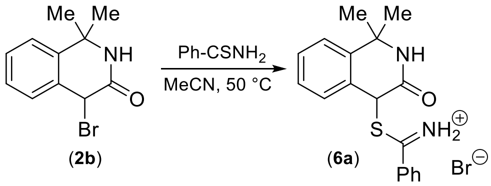

Compound **2b** (0.51 g, 2 mmol) was dissolved in acetonitrile (25 mL) at 50 °C and a hot solution (50 °C) of thiobenzamide (0.27 g, 2 mmol) in acetonitrile (5 mL) was added in one portion. The reaction mixture was stirred for 30 min at 50 °C and then cooled. The precipitated salt was filtered off, washed with cold acetonitrile (5 mL) and diethylether (10 mL). Yield 0.76 g (97%) of white solid with m.p. 176-177 °C. HRMS: calcd. for $C_{18}H_{19}N_2OS$ [M+H$^+$]: calcd. 311.1213, found 311.1216; Elemental analysis: calcd. C: 55.25, H: 4.89, N: 7.16, Br: 20.42; S: 8.19; found C: 55.15, H: 4.92, N: 7.12, S: 8.26, Br: 20.51. IR (cm^{-1}): 3025 (s), 2891 (s), 1635 (s), 1423 (m), 1329 (m), 767 (m).

Transformation of salt 6a to 5,5-dimethyl-2-phenyl-4,5-dihydrothiazolo[4,5-*c*]isoquinoline (8a) and 4,5,5-trimethyl-2-phenyl-4,5-dihydrothiazolo[4,5-*c*]isoquinoline (8a-Me)

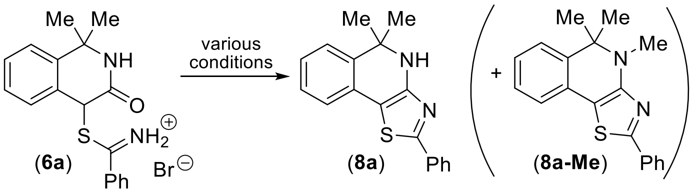

In DMF:

Salt **6a** (100 mg, 0.25 mmol) was dissolved in DMF (0.5 mL) at room temperature and stirred for 12 h. The reaction mixture was then poured into cold water (50 mL) and extracted with EtOAc (2 × 10 mL). The organic layer was dried with anhydrous Na_2SO_4 and evaporated to give 90 mg (74%) of orange solid **8a** with mp 86-89 °C. ^{1}H NMR (500 MHz, $CDCl_3$) δ 7.86-7.94 (m, 2H, Ar-H), 7.35-7.42 (m, 3H, Ar-H), 7.19 (td, *J* 7.2 and 1.3 Hz, 2H, Ar-H), 7.13 (td, *J* 7.2 and 1.3 Hz, 2H, Ar-H), 4.62 (bs, 1H, NH), 1.56 (s, 6H, 2×CH_3); ^{13}C NMR (125 MHz, $CDCl_3$) δ 164.4 (C), 155.4 (C), 136.5 (C), 133.7 (C), 129.8 (CH), 128.8 (CH), 128.4 (C), 127.5

(CH), 126.2 (CH), 125.8 (CH), 123.6 (CH), 122.3 (CH), 105.1 (C), 55.9 (C), 29.8 (CH_3); HRMS: calcd. for $C_{18}H_{17}N_2S$ [M+H$^+$]: calcd. 293.1113, found 293.1108; Elemental analysis: calcd. C: 73.94, H: 5.52, N: 9.58, S: 10.96; found C: 73.69, H: 5.56, N: 9.81, S: 11.02.

In MeCN:

Salt **6a** (400 mg, 1 mmol) was suspended in MeCN (30 mL) and stirred for 12 h at 70 °C in a tightly closed heavy-wall screw-capped vial. The resulting solution was neutralized with Et_3N (0.15 mL, 1.1 mmol) and evaporated with non-porous alumina. Column chromatography (silica gel, petrolether/EtOAc 4:1) gave 0.21 g (72%) of compound **8a** with the characteristics mentioned above.

In (MeO)$_3$P:

Salt **6a** (186 mg, 0.5 mmol) was suspended in neat trimethyl phosphite (2 mL) and stirred without (entry 1 in the table below) or with an appropriate additive (TFA: entry 2; TEA: entry 3 in table below) for 12 h at 70 °C. The resulting solution was then diluted with CH_2Cl_2 (10 mL) and evaporated with non-porous alumina (5 g). The residue was submitted to column chromatography (silica gel) and eluted with hexane/EtOAc 95:5 to give **8a-Me** and then with hexane/EtOAc 60:40 to give product **8a** in the yields given below:

Entry	Additive (equiv)	Yield **8a** mg (%)	Yield **8a-Me** mg (%)	Overall yield **8a** + **8a-Me** (%)
1	None	53 (36)	26 (16)	52
2	CF_3COOH (0.8)	93 (61)	41(26)	87
3	Et_3N (0.7)	40 (27)	29 (18)	45

Compound **8a-Me**: mp 104-105 °C. ^{1}H NMR (500 MHz, $CDCl_3$) δ 7.94 (dd, *J* 8.2 and 1.2 Hz, 2H, Ar-H), 7.33-7.42 (m, 3H, Ar-H), 7.24 (d, *J* 7.8 Hz, 1H, Ar-H), 7.18 (dt, *J* 7.4 and 1.1 Hz, 1H, Ar-H), 7.08-7.13 (m, 2H, Ar-H), 3.27 (s, 3H, NCH_3), 1.56 (s, 6H, 2×CH_3); ^{13}C NMR (125 MHz, $CDCl_3$) δ 162.8 (C), 157.6 (C), 136.2 (C), 134.0 (C), 129.5 (CH), 128.8 (CH), 128.7 (C), 127.5 (CH), 125.9 (CH), 125.8 (CH), 123.7 (CH), 122.0 (CH), 104.9 (C), 59.7 (C), 31.0 (NCH_3), 24.3 (CH_3); HRMS: calcd. for $C_{19}H_{19}N_2S$ [M+H$^+$]: calcd. 307.1264, found 307.1264; Elemental analysis: calcd. C: 74.47, H: 5.92, N: 9.14, S: 10.46; found C: 74.71, H: 5.90, N: 9.01, S: 10.42; IR (cm^{-1}): 3064 (m), 2975 (m), 1597 (m), 1550 (s), 1457 (m), 752 (m).

Synthesis of 8b

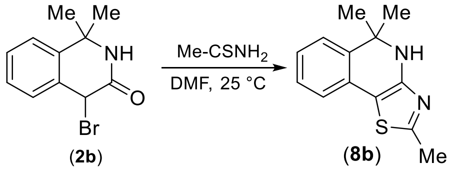

Compound **2b** (254 mg, 1 mmol) was dissolved in DMF (1.5 mL) at 25 °C, then a solution of thioacetamide (75 mg, 1 mmol) in DMF (1 mL) was added in one portion and the mixtures

stirred for 16 h. All volatiles were evaporated in vacuo and the residue was dissolved in mixture of DCM and MeOH (3:1, 20 mL). The solution was co-evaporated with non-porous alumina (5 g) and the residue was submitted to column chromatography (silica gel) eluted with petrolether/EtOAc 4:1 – 2:1 to give **8b** with mp 104-107 °C. ^{1}H NMR (500 MHz, DMSO-d_6) δ 7.19 (d, *J* 7.5 Hz, 1H, Ar-H), 7.13 (t, *J* 7.5 Hz, 1H, Ar-H), 7.05 (t, *J* 7.5 Hz, 1H, Ar-H), 6.91 (d, *J* 7.5 Hz, 1H, Ar-H), 6.66 (bs, 1H, NH), 2.57 (s, 3H, CH_3), 1.44 (s, 6H, 2×CH_3); ^{13}C NMR (125 MHz, DMSO-d_6) δ 163.2 (C), 155.4 (C), 136.1 (C), 128.7 (C), 127.3 (CH), 125.3 (CH), 123.6 (CH), 121.1 (CH), 100.2 (C), 55.2 (C), 29.8 (CH_3), 19.4 (CH_3); HRMS: calcd. for $C_{13}H_{15}N_2S$ [M+H$^+$]: calcd. 231.0951, found 231.0951; Elemental analysis: calcd. C: 67.79, H: 6.13, N: 12.16, S: 13.92; found C: 67.42, H: 6.16, N: 12.31, S: 14.02.

Eschenmoser coupling reaction giving 9a,b

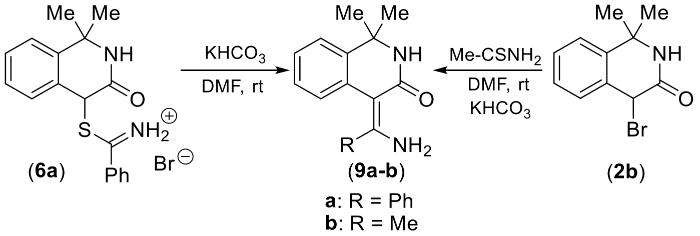

Salt **6a** (300 mg, 0.77 mmol) and finely powdered $KHCO_3$ (230 mg, 2.3 mmol) were suspended in DMF (5 mL) and stirred in screw-capped vial for 48 h at room temperature. Then, DCM (10 mL) was added and the reaction mixture was filtered. The solid residue was washed with DCM (10 mL) and the combined filtrates were co-evaporated with non-porous alumina (5 g) and subjected to automated flash chromatography (25 g column with spherical SiO_2; hexane/EtOAc - gradient 10–100% EtOAc) to give product **9a**.

(*Z*)-4-(Amino(phenyl)methylidene)-1,1-dimethyl-1,4-dihydroisoquinolin-3(2*H*)-one (9a)
Yellow crystalline solid (132 mg, 62 %); mp 227 – 229 °C; ^{1}H NMR (500 MHz, $CDCl_3$) δ: 9.60 (bs, 1H, ½NH_2), 7.35-7.43 (m, 3H, Ar-H_{o+p}), 7.29-7.34 (m, 2H, Ar-H_m), 7.15 (dd, *J* 7.7 and 0.9 Hz, 1H, Ar-H_8), 6.91 (dt, *J* 7.6 and 1.1 Hz, 1H, Ar-H), 6.71 (dt, *J* 7.6 and 1.2 Hz, 1H, Ar-H), 6.41 (dd, *J* 8.0 and 0.8 Hz, 1H, Ar-H_5), 5.76 (bs, 1H, NH), 5.58 (bs, 1H, ½NH_2), 1.62 (s, 6H, 2×CH_3); ^{13}C NMR (125 MHz, $CDCl_3$) δ (ppm): 171.1 (C=O), 156.1 (C), 139.3 (C), 138.7 (C), 133.2 (C), 129.6 (CH), 128.9 (CH), 128.7 (CH), 128.4 (CH), 125.6 (CH), 123.6 (CH), 121.9 (CH), 96.7 (C), 54.3 (C), 29.5 (CH_3); HRMS: calcd. for $C_{18}H_{19}N_2O$ [M+H$^+$]: calcd. 279.1492, found 279.1490; Elemental analysis: calcd. C: 77.67, H: 6.52, N: 10.06; found C: 77.66, H: 6.56, N: 10.32.

Compound **9b** was prepared from **2b** and thioacetamide without the isolation of intermediary salt **6b** as follows. Compound **2b** (0.51 g, 2 mmol) was dissolved in DMF (2 mL) at room temperature and a solution of thioacetamide (0.15 g, 2 mmol) in 1.5 mL of DMF was added in one portion. Then, finely powdered $KHCO_3$ (0.6 g, 6 mmol) was added and the reaction mixture was stirred at 25 °C for 48 h. Then, the reaction mixture was diluted with MeCN (25 mL) and filtered through a plug of cellite. All volatiles were evaporated, the residue was dissolved in

MeOH (20 mL), and co-evaporated again with non-porous alumina (5 g). Column chromatography (silica gel, petrolether/EtOAc 50:50–0:100 and then MeOH/EtOAc 5:95) gave compound **9b**.

(*Z*)-4-(1-Aminoethylidene)-1,1-dimethyl-1,4-dihydroisoquinolin-3(2*H*)-one (9b)

White crystalline solid (209 mg, 48 %); mp 186 – 188 °C; ^{1}H NMR (500 MHz, $CDCl_3$) δ: 9.24 (vbs, 1H, ½NH_2), 7.16-7.24 (m, 2H, Ar-H), 7.12 (d, *J* 7.3 Hz, 1H, Ar-H), 7.07 (t, *J* 7.5 Hz, 1H, Ar-H), 5.82 (bs, 1H, NH), 4.55 (vbs, 1H, ½NH_2), 2.25 (s, 3H, CH_3), 1.50 (s, 6H, 2×CH_3); ^{13}C NMR (125 MHz, $CDCl_3$) δ (ppm): 171.2 (C), 153.8 (C), 139.9 (C), 133.6 (C), 126.6 (CH), 126.1 (CH), 123.8 (CH), 122.1 (CH), 97.0 (C), 54.2 (C), 29.3 (CH_3), 21.8 (CH_3); HRMS: calcd. for $C_{13}H_{17}N_2O$ [M+H^{+}]: calcd. 217.1335, found 217.1338; Elemental analysis: calcd. C: 72.19, H: 7.46, N: 12.95; found C: 71.94, H: 7.46, N: 12.91.

Synthesis of 9c–h

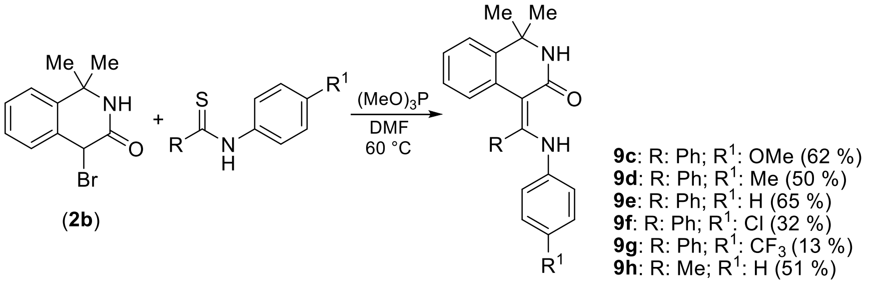

Compound **2b** (0.51 g, 2 mmol) was dissolved in DMF (5 mL) at room temperature and a solution of the corresponding secondary thioamide (2 mmol in 1.5 mL of DMF) was added in one portion. After 5 min, trimethyl phosphite (295 µL, 2.5 mmol, 1.25 equiv) was added and the reaction mixture was stirred at 60 °C for 16 h. Then, all volatiles were evaporated, the residue was dissolved in $CHCl_3$/EtOH (1:1, 10 mL), and co-evaporated again with non-porous alumina (5 g). Column chromatography (silica gel, *n*-hexane/EtOAc 6:1–4:1) gave compounds **9c–h**.

(*Z*)-4-{[(4-Methoxyphenyl)amino][phenyl]methylidene}-1,1-dimethyl-1,4-dihydroisoquinolin-3(2*H*)-one (9c)

Yellow solid (477 mg, 62 %); mp 209 – 211 °C; ^{1}H NMR (500 MHz, $CDCl_3$) δ: 11.76 (bs, 1H, NH), 7.21-7.27 (m, 3H, Ar-H), 7.11-7.21 (m, 3H, Ar-H), 6.91 (t, *J* 7.5, 1H, Ar-H), 6.69 (t, *J* 7.6 Hz, 1H, Ar-H), 6.55-6.65 (m, 4H, Ar-H), 6.33 (d, *J* 8.0 Hz, 1H, Ar-H), 6.00 (s, 1H, Ar-H), 3.68 (s, 3H, OCH_3), 1.64 (s, 6H, 2×CH_3); ^{13}C NMR (125 MHz, $CDCl_3$) δ (ppm): 171.3 (C), 155.3 (C), 153.5 (C), 139.9 (C), 134.6 (C), 133.7 (C), 133.1 (C), 130.9 (CH), 129.0 (CH), 128.8 (CH), 128.5 (CH), 125.5 (CH), 113.6 (CH), 99.7 (C), 55.3 (OCH_3), 54.3 (C), 29.2 (CH_3); HRMS: calcd. for $C_{25}H_{25}N_2O_2$ [M+H^{+}]: calcd. 385.1911, found 385.1910; Elemental analysis: calcd. C: 78.10, H: 6.29, N: 7.29; found C: 78.30, H: 6.37, N: 7.36.

(*Z*)-4-{[(4-Methylphenyl)amino][phenyl]methylidene}-1,1-dimethyl-1,4-dihydroisoquinolin-3(2*H*)-one (9d)

Crude product obtained after chromatography was crystallized from MeOH/$CHCl_3$ (5:1). Yellow solid (368 mg, 50 %); mp 253 – 254 °C; ^{1}H NMR (500 MHz, $CDCl_3$) δ: 11.78 (bs, 1H, NH), 7.23-7.30 (m, 3H, Ar-H), 7.17-7.23 (m, 2H, Ar-H), 7.16 (d, *J* 7.6, 1H, Ar-H), 6.92 (t, *J* 7.4 Hz, 1H, Ar-H), 6.84 (d, *J* 7.9 H, 2H, Ar-H), 6.69 (t, *J* 7.5 Hz, 1H, Ar-H), 6.53 (d, *J* 8.0 Hz, 2H, Ar-H), 6.34 (d, *J* 7.9 Hz, 1H, Ar-H), 5.86 (s, 1H, Ar-H), 2.18 (s, 3H, CH_3), 1.65 (s, 6H, 2×CH_3); ^{13}C NMR (125 MHz, $CDCl_3$) δ (ppm): 171.2 (C), 152.8 (C), 140.0 (C), 138.0 (C), 134.7 (C), 133.1 (C), 132.0 (C), 130.8 (CH), 129.1 (CH), 129.0 (CH), 128.9 (CH), 128.6 (CH), 125.6 (CH), 123.8 (CH), 122.7 (CH), 121.8 (CH), 100.3 (C), 54.4 (C), 29.2 (CH_3), 20.7 (CH_3); HRMS: calcd. for $C_{25}H_{25}N_2O$ [M+H$^+$]: calcd. 369.1961, found 369.1960; Elemental analysis: calcd. C: 81.49, H: 6.57, N: 7.60; found C: 81.48, H: 6.52, N: 7.77.

(*Z*)-1,1-Dimethyl-4-[phenyl(phenylamino)methylidene]-1,4-dihydroisoquinolin-3(2*H*)-one (9e)

Yellow solid (461 mg, 65 %); mp 231 – 232 °C; ^{1}H NMR (500 MHz, $CDCl_3$) δ: 11.81 (bs, 1H, NH), 7.24-7.30 (m, 3H, Ar-H), 7.18-7.23 (m, 2H, Ar-H), 7.16 (d, *J* 7.7, 1H, Ar-H), 7.03 (t, *J* 7.7 Hz, 1H, Ar-H), 6.93 (t, *J* 7.4 H, 2H, Ar-H), 6.84 (t, *J* 7.3 Hz, 1H, Ar-H), 6.70 (t, *J* 7.5 Hz, 2H, Ar-H), 6.62 (d, *J* 7.8 Hz, 1H, Ar-H), 6.36 (d, *J* 7.9 Hz, 1H, Ar-H), 6.26 (s, 1H, Ar-H), 1.65 (s, 6H, 2×CH_3); ^{13}C NMR (125 MHz, $CDCl_3$) δ (ppm): 171.2 (C), 152.1 (C), 140.7 (C), 140.3 (C), 134.5 (C), 132.9 (C), 130.8 (CH), 129.2 (CH), 129.0 (CH), 128.6 (CH), 128.4 (CH), 125.6 (CH), 124.0 (CH), 122.4 (CH), 122.3 (CH), 121.8 (CH), 101.1 (C), 54.4 (C), 29.1 (CH_3); HRMS: calcd. for $C_{24}H_{22}N_2O$ [M+H$^+$]: calcd. 355.1805, found 355.1806; Elemental analysis: calcd. C: 81.33, H: 6.26, N: 7.90; found C: 81.16, H: 6.30, N: 7.93.

(*Z*)-4-{[(4-Chlorophenyl)amino][phenyl]methylidene}-1,1-dimethyl-1,4-dihydroisoquinolin-3(2*H*)-one (9f)

Yellow solid (251 mg, 32 %); mp 222 – 224 °C; ^{1}H NMR (500 MHz, $CDCl_3$) δ: 11.78 (bs, 1H, NH), 7.27-7.33 (m, 1H, Ar-H), 7.20-7.26 (m, 4H, Ar-H), 7.17 (d, *J* 7.7, 1H, Ar-H), 6.90-7.03 (m, 3H, Ar-H), 6.71 (t, *J* 7.4 Hz, 1H, Ar-H), 6.53 (d, *J* 8.6 Hz, 2H, Ar-H), 6.36 (d, *J* 7.9 Hz, 1H, Ar-H), 6.25 (s, 1H, Ar-H), 1.65 (s, 6H, 2×CH_3); ^{13}C NMR (125 MHz, $CDCl_3$) δ (ppm): 171.1 (C), 151.5 (C), 140.3 (C), 139.4 (C), 134.2 (C), 132.6 (C), 130.7 (CH), 129.4 (CH), 129.0 (CH), 128.8 (CH), 128.4 (C), 127.4 (CH), 125.7 (CH), 124.3 (CH), 123.4 (CH), 121.9 (CH), 101.7 (C), 54.5 (C), 29.2 (CH_3); HRMS: calcd. for $C_{24}H_{22}ClN_2O$ [M+H$^+$]: calcd. 389.1415, found 389.1417; Elemental analysis: calcd. C: 74.12, H: 5.44, N: 7.20; found C: 74.34, H: 5.46, N: 7.09.

(*Z*)-4-{[(4-Trifluormethylphenyl)amino][phenyl]methylidene}-1,1-dimethyl-1,4-dihydroisoquinolin-3(2*H*)-one (9g)

Crude product obtained after chromatography was crystallized from MeOH/EtOAc (2:1). Yellow solid (112 mg, 13 %); mp 234 – 236 °C; ^{1}H NMR (500 MHz, $CDCl_3$) δ: 11.86 (bs, 1H, NH), 7.32-7.37 (m, 1H, Ar-H), 7.23-7.30 (m, 6H, Ar-H), 7.19 (d, *J* 7.7, 1H, Ar-H), 6.98 (t, *J*

7.4 Hz, 1H, Ar-H), 6.74 (d, *J* 7.5 H, 2H, Ar-H), 6.60 (d, *J* 8.3 Hz, 2H, Ar-H), 6.40 (d, *J* 7.9 Hz, 1H, Ar-H), 6.06 (s, 1H, Ar-H), 1.67 (s, 6H, 2×CH_3); ^{13}C NMR (125 MHz, $CDCl_3$) δ (ppm): 170.9 (C), 150.3 (C), 144.1 (C), 140.6 (C), 134.0 (C), 132.2 (C), 130.6 (CH), 129.7 (CH), 129.2 (CH), 129.0 (CH), 125.8 (CH), 125.7 (C, q, $^3J_{C-F}$ = 3.7 Hz), 124.7 (CH), 124.3 (C, q, $^1J_{C-F}$ = 269.5 Hz), 123.4 (C, q, $^2J_{C-F}$ = 32.7 Hz), 122.01 (CH), 121.0 (CH), 103.2 (C), 54.7 (C), 29.4 (CH_3); HRMS: calcd. for $C_{25}H_{22}F_3N_2O$ [M+H$^+$]: calcd. 423.1679, found 423.1682; Elemental analysis: not measured due to fluorine content damaging column.

(*Z*)-1,1-Dimethyl-4-[1-(phenylamino)ethylidene]-1,4-dihydroisoquinolin-3(2*H*)-one (9h)
Yellow solid (296 mg, 51 %); mp 191 – 193 °C; ^1H NMR (500 MHz, $CDCl_3$) δ: 12.05 (bs, 1H, NH), 7.34 (t, *J* 7.7 Hz, 1H, Ar-H), 7.20-7.27 (m, 3H, Ar-H), 7.14-7.19 (m, 3H, Ar-H), 7.07-7.12 (m, 1H, Ar-H), 5.74 (s, 1H, Ar-H), 2.34 (s, 3H, CH_3), 1.55 (s, 6H, 2×CH_3); ^{13}C NMR (125 MHz, $CDCl_3$) δ (ppm): 171.3 (C), 153.5 (C), 140.1 (C), 139.7 (C), 133.6 (C), 128.9 (CH), 127.0 (CH), 126.2 (CH), 125.0 (CH), 124.7 (CH), 124.0 (CH), 122.2 (CH), 98.4 (CH), 54.3 (C), 29.4 (CH_3), 19.5 (CH_3); HRMS: calcd. for $C_{19}H_{21}N_2O$ [M+H$^+$]: calcd. 293.1648, found 293.1646; Elemental analysis: calcd. C: 78.05, H: 6.89, N: 9.57; found C: 78.32, H: 7.00, N: 9.35.

Synthesis of 11a–c
Compound **3** (0.24 g, 1 mmol) was dissolved in DMF (1.5 mL) at room temperature and a solution of the corresponding thioamide (1 mmol) in DMF (1.5 mL) was added in one portion and stirred for 12 h at room temperature. Then, all volatiles were evaporated with non-porous alumina (2 g), and the residue was submitted to flash column chromatography (silica gel cartridge, CH_2Cl_2/MeOH gradient 0–8% during 15 min) giving compounds **11a–c**.

(*Z*)-4-(Amino(phenyl)methylidene)isoquinoline-1,3(2*H*,4*H*)-dione (11a)
Yellow solid (0.2 g, 75 %); mp 282 – 283 °C; ^1H NMR (500 MHz, DMSO-d_6) δ: 11.08 (bs, 1H, NH), 10.80 (bd, *J* 2.8 Hz, 1H, ½NH_2), 8.60 (bd, *J* 2.4 Hz, 1H, ½NH_2), 7.93 (dd, *J* 7.6 and 1.6 Hz, 1H, Ar-H), 7.57 (t, *J* 7.4 Hz, 1H, Ar-H), 7.49 (t, *J* 7.6, 2H, Ar-H_m), 7.42 (d, *J* 7.2 Hz, 2H, Ar-H_o), 6.93-7.03 (m, 2H, Ar-H), 6.32 (d, *J* 8.0 Hz, 1H, Ar-H); ^{13}C NMR APT (125 MHz, DMSO-d_6) δ (ppm): 166.9 (C=O), 166.1 (C=O), 163.9 (C), 137.7 (C), 137.4 (C), 131.0 (CH), 130.7 (CH), 129.3 (CH), 128.6 (CH), 127.2 (CH), 125.5 (CH), 122.7 (CH), 122.4 (C), 92.4 (C); HRMS: calcd. for $C_{16}H_{13}N_2O_2$ [M+H$^+$]: calcd. 265.0972, found 265.0974; Elemental analysis: calcd. C: 72.72, H: 4.58, N: 10.60; found C: 72.55, H: 4.51, N: 10.40.

(*Z*)-4-(1-Aminoethylidene)isoquinoline-1,3(2*H*,4*H*)-dione (11b)
Light yellow solid (0.16 g, 78 %); mp 278 – 280.5 °C; ^1H NMR (500 MHz, DMSO-d_6) δ: 10.87 (bs, 1H, NH), 10.80 (bs, 1H, ½NH_2), 8.47 (bs, 1H, ½NH_2), 8.03 (d, *J* 7.8 Hz, 1H, Ar-H), 7.61 (d, *J* 8.3 Hz, 1H, Ar-H), 7.53 (t, *J* 7.7, 2H, Ar-H), 7.18 (t, *J* 7.3 Hz, 1H, Ar-H), 2.51 (s, 3H, CH_3); ^{13}C NMR (125 MHz, DMSO-d_6) δ (ppm): 166.4, 165.9, 163.7, 138.0, 132.2, 127.5, 124.7, 122.1, 93.2, 23.8; HRMS: calcd. for $C_{11}H_{11}N_2O_2$ [M+H$^+$]: calcd. 203.0815, found 203.0816; Elemental analysis: calcd. C: 65.34, H: 4.98, N: 13.85; found C: 65.40, H: 4.95, N: 13.99.

(*Z*)-4-(Phenyl(phenylamino)methylidene)isoquinoline-1,3(2*H*,4*H*)-dione (11c)
Yellow solid (0.3 g, 91 %); mp 268 – 270 °C; ^{1}H NMR (500 MHz, DMSO-d_6) δ: 13.12 (bs, 1H, NH), 11.41 (bs, 1H, NH), 7.97 (d, *J* 7.7 Hz, 1H, Ar-H), 7.44 (t, *J* 7.2 Hz, 1H, Ar-H), 7.24-7.39 (m, 4H, Ar-H), 7.15 (t, *J* 7.8, 2H, Ar-H), 7.01-7.08 (m, 2H, Ar-H), 6.96 (t, *J* 7.7 Hz, 1H, Ar-H), 6.86 (d, *J* 7.8 Hz, 2H, Ar-H) 6.25 (d, *J* 8.2 Hz, 1H, Ar-H); ^{13}C NMR APT (125 MHz, DMSO-d_6) δ (ppm): 167.7 (C=O), 163.8 (C=O), 161.7 (C), 138.4 (C), 136.9 (C), 133.5 (C), 131.1 (CH), 130.5 (CH), 130.2 (CH), 129.4 (CH), 128.8 (CH), 127.3 (CH), 126.1 (CH), 125.4 (CH), 125.1 (CH), 123.5 (CH), 123.2 (C), 96.1 (C); HRMS: calcd. for $C_{22}H_{17}N_2O_2$ [M+H$^+$]: calcd. 341.1285, found 341.1288; Elemental analysis: calcd. C: 77.63, H: 4.74, N: 8.23; found C: 77.90, H: 4.66, N: 8.19.

Experiments with 4a

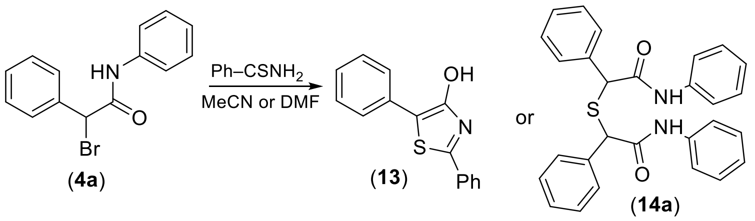

N-Phenyl-2-bromo(phenyl)acetamide (**4a**, 145 mg, 0.5 mmol) was dissolved in MeCN or DMF (2.5 mL) and a solution of thiobenzamide (69 mg, 0.5 mmol) in the same solvent (2.5 ml) was added in one portion. The reaction mixture was stirred for 16 h at room temperature. In some experiments the base/thiophilic agent were also present. The crude reaction mixture was evaporated under vacuum and a sample was subjected to ^{1}H NMR analysis (see Table 3 in the main text). Pure constituents present in reaction mixture were obtained using column chromatography (silica gel, petrolether/DCM/EtOAc 250:100:40).

4-Hydroxy-2,5-diphenyl-1,3-thiazole (**13**) and thiobenzamide were identified by comparison of their ^{1}H and ^{13}C NMR spectra in DMSO-d_6 with original samples prepared previously [8] in our laboratory.

2,2'-Thiobis(*N*-phenyl-2-phenylacetamide) (14a)

White solid (48 mg, 21 %); mp 212 – 215 °C; ^{1}H NMR (500 MHz, DMSO-d_6) δ: 10.22 and 10.26 (2×bs, 2H, 2×NH), 7.56 and 7.52 (2×d, *J* 7.9 and 7.5 Hz, 4H, Ar-H), 7.44-7.50 (m, 4H, Ar-H), 7.22-7.42 (m, 10H, Ar-H), 7.06 and 7.03 (2×t, *J* 7.4 Hz, 2H, Ar-H), 4.69 and 4.63 (2×s, 1H, 2×CH); ^{13}C NMR (125 MHz, DMSO-d_6) δ (ppm): 167.7 and 167.6 (C=O), 138.8 and 138.7 (C), 137.4 and 137.2 (C), 128.9 and 128.8 (CH), 128.8 and 128.7 (CH), 128.4 and 128.3 (CH), 128.2 and 128.1 (CH), 123.9 and 123.8 (CH), 119.6, 119.5 (CH), 53.8 and 53.5 (CH); HRMS: calcd. for $C_{28}H_{25}N_2O_2S$ [M+H$^+$]: calcd. 453.1631, found 453.1639; Elemental analysis: calcd. C: 74.31, H: 5.35, N: 6.19, S: 7.09; found C: 74.64, H: 5.42, N: 5.82, S: 7.22.
Note: Both NMR spectra consist of two close sets of signals mutually approaching at heating. Unfortunately, the coalescence was not attained even at the highest temperature (75 °C) of measurement. Another explanation involves two sets of signals for diastereoisomers (set of two enantiomers and *meso*-form).

Experiments with 4b

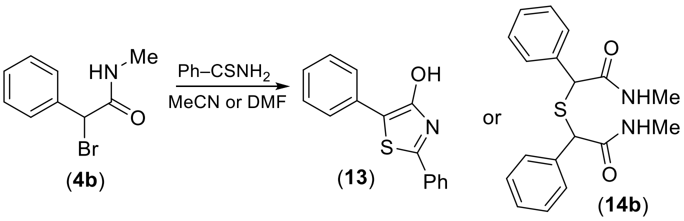

N-Methyl-2-bromo(phenyl)acetamide (456 mg, 2 mmol) was dissolved in MeCN (10 mL) and a solution of thiobenzamide (274 mg, 2 mmol) in MeCN (5 ml) was added, followed by finely powdered $KHCO_3$ (600 mg, 6 mmol). The reaction mixture was stirred for 16 h at room temperature. Reaction contents were then filtered, and the filter cake washed with additional MeCN (5 mL). The filtrate was evaporated in vacuo with non-porous alumina (5 g) and subsequent column chromatography (silica gel, DCM/EtOAc/MeOH 30:10:1) gave yellow solid (124 mg) which was triturated with $CHCl_3$ (3 mL) and filtered.

4-Hydroxy-2,5-diphenyl-1,3-thiazole (**13**) and thiobenzamide were identified by comparison of their ^{1}H and ^{13}C NMR spectra in DMSO-d_6 with original samples prepared previously [8] in our laboratory.

2,2'-Thiobis(*N*-methyl-2-phenylacetamide) (14b)

White solid (104 mg, 16 %); mp 223 – 226 °C; ^{1}H NMR (500 MHz, DMSO-d_6) δ: 8.16 and 8.05 (2×bq, *J* 4.5 Hz, 1H, NH), 7.25-7.43 (m, 5H, Ar-H), 4.36 and 4.26 (2×s, 1H, CH-S), 2.59 and 2.48 (2×d, *J* 4.5 Hz, 3H, CH_3); ^{13}C NMR (125 MHz, DMSO-d_6) δ (ppm): 169.2 and 169.2 (C=O), 137.9 and 137.7, 128.4 and 128.1 (CH), 128.3 and 127.7 (CH), 127.7 and 127.5 (CH), 53.2 and 53.0 (CH), 26.0 and 25.8 (CH_3); HRMS: calcd. for $C_{18}H_{21}N_2O_2S$ [M+H$^+$]: calcd. 329.1318, found 329.1323; Elemental analysis: calcd. C: 65.83, H: 6.14, N: 8.53, S: 9.76; found C: 65.72, H: 6.18, N: 8.89, S: 9.42.
Note: Both NMR spectra consist of two close sets of signals (ratio 1:4) mutually approaching at heating. Unfortunately, the coalescence was not attained even at the highest temperature (75 °C) of measurement. Another explanation involves two sets of signals for diastereoisomers (set of two enantiomers and *meso*-form).

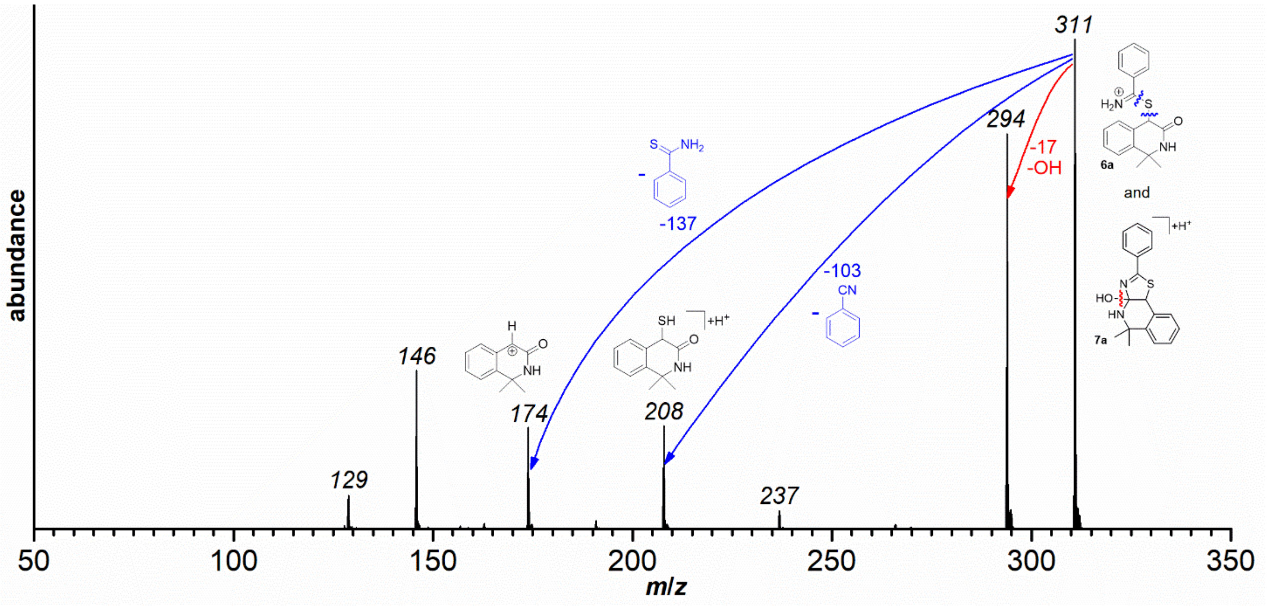

Figure S1. ESI-MS CID spectrum showing different fragmentation patterns of isobaric compounds **6a** and **7a**.

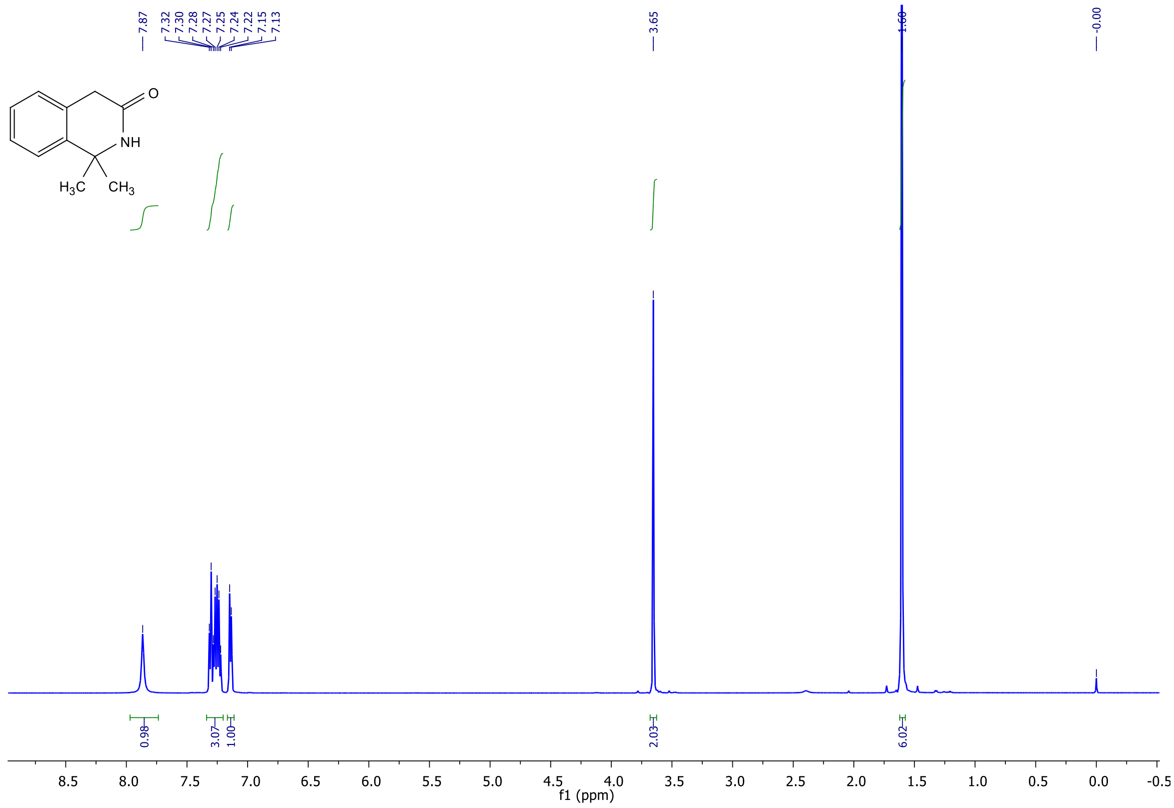
7.87
7.32
7.30
7.28
7.27
7.25
7.24
7.22
7.15
7.13
3.65
1.60
-0.00
O
NH
H3C
CH3
0.98
3.07
1.00
2.03
6.02
f1 (ppm)

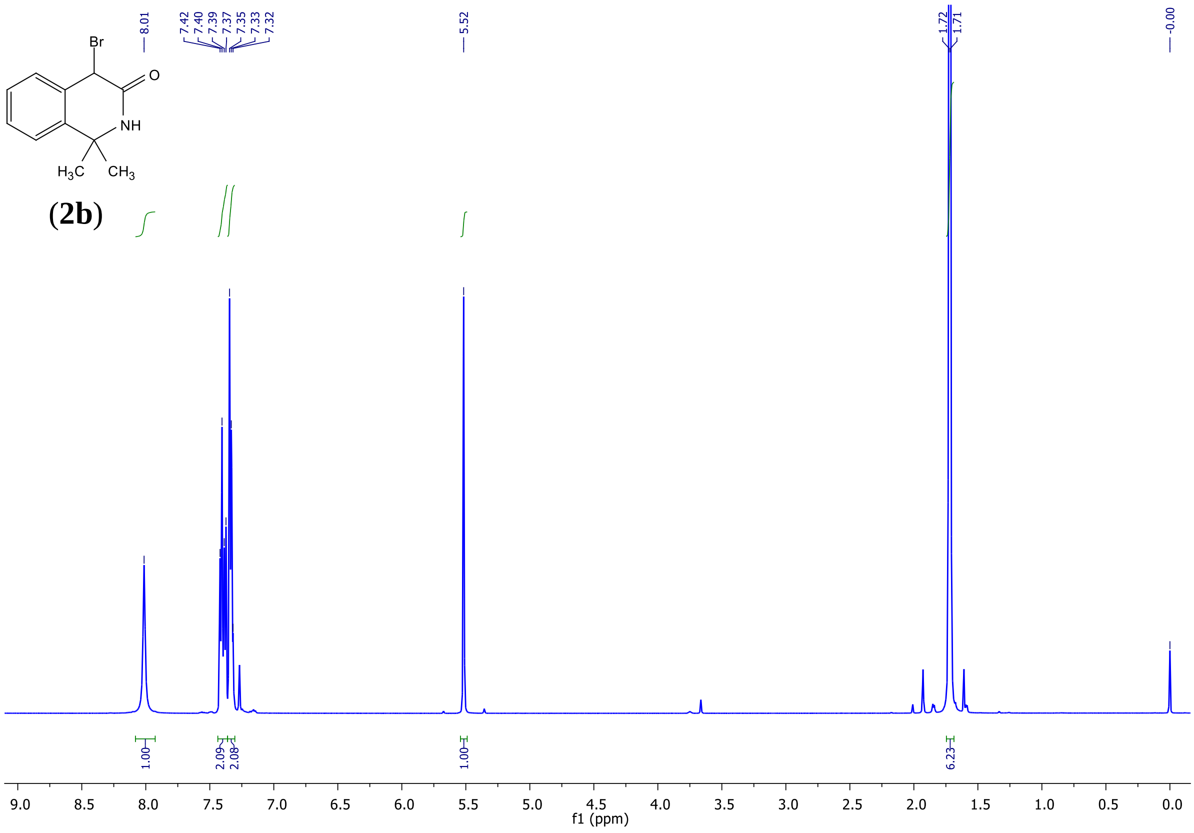
8.01
7.42
7.40
7.39
7.37
7.35
7.33
7.32
5.52
1.72
1.71
-0.00
Br
O
NH
H3C
CH3
1.00
2.09
2.08
1.00
6.23
f1 (ppm)

(2b)

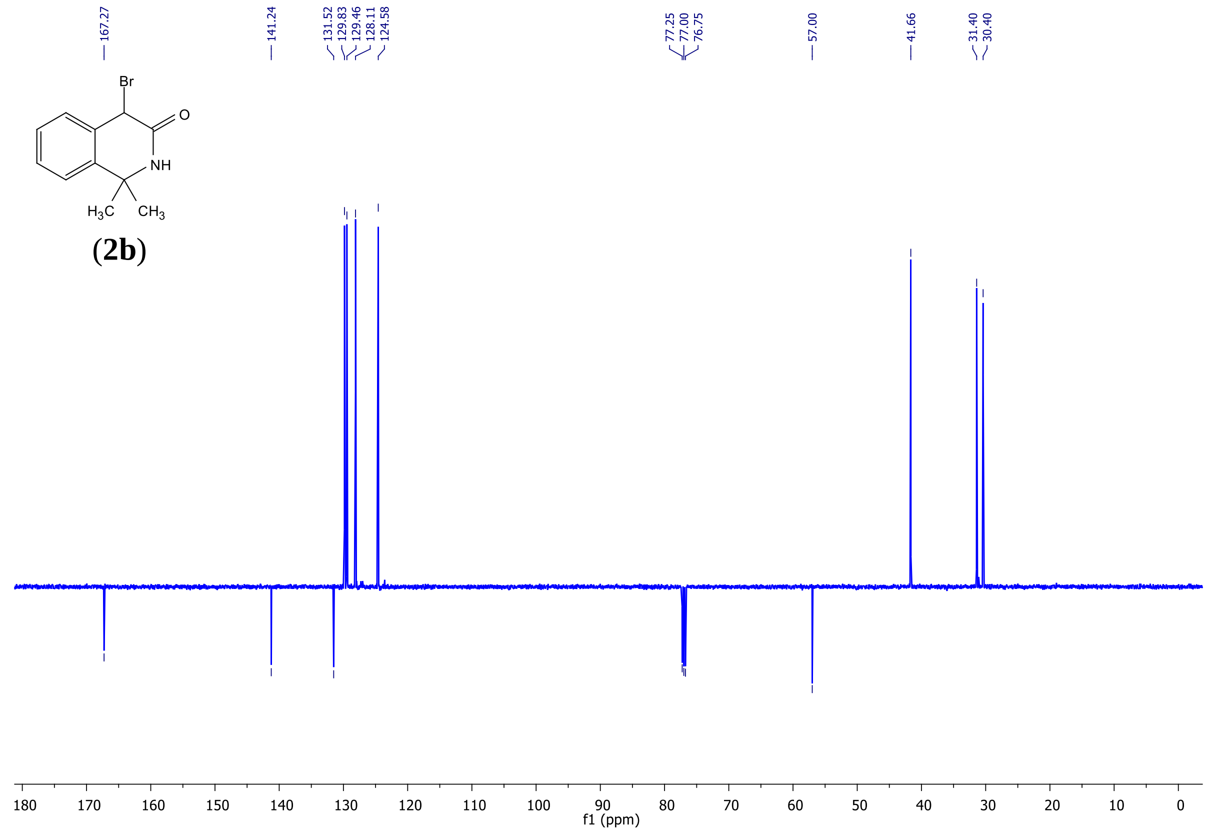

Br
O
NH
H3C
CH3
(2b)
f1 (ppm)

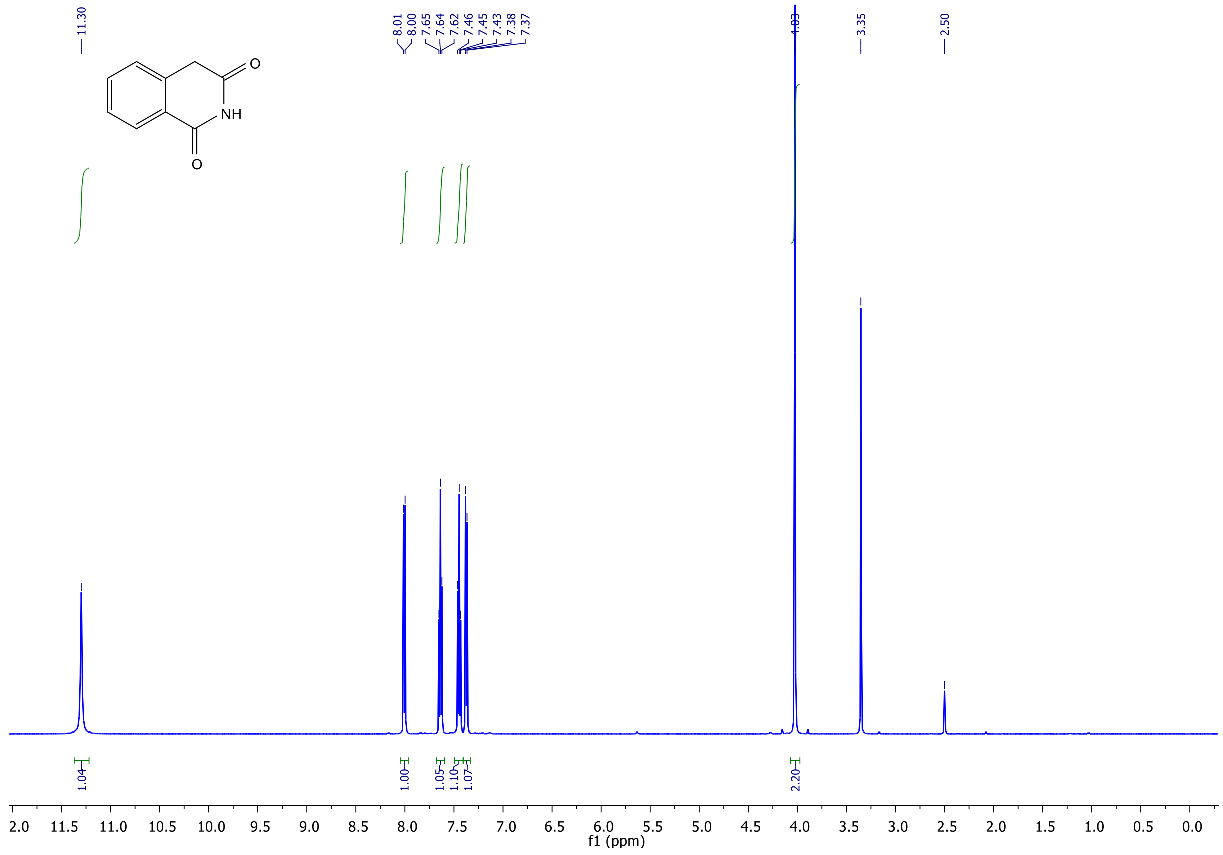

O
NH
O
f1 (ppm)

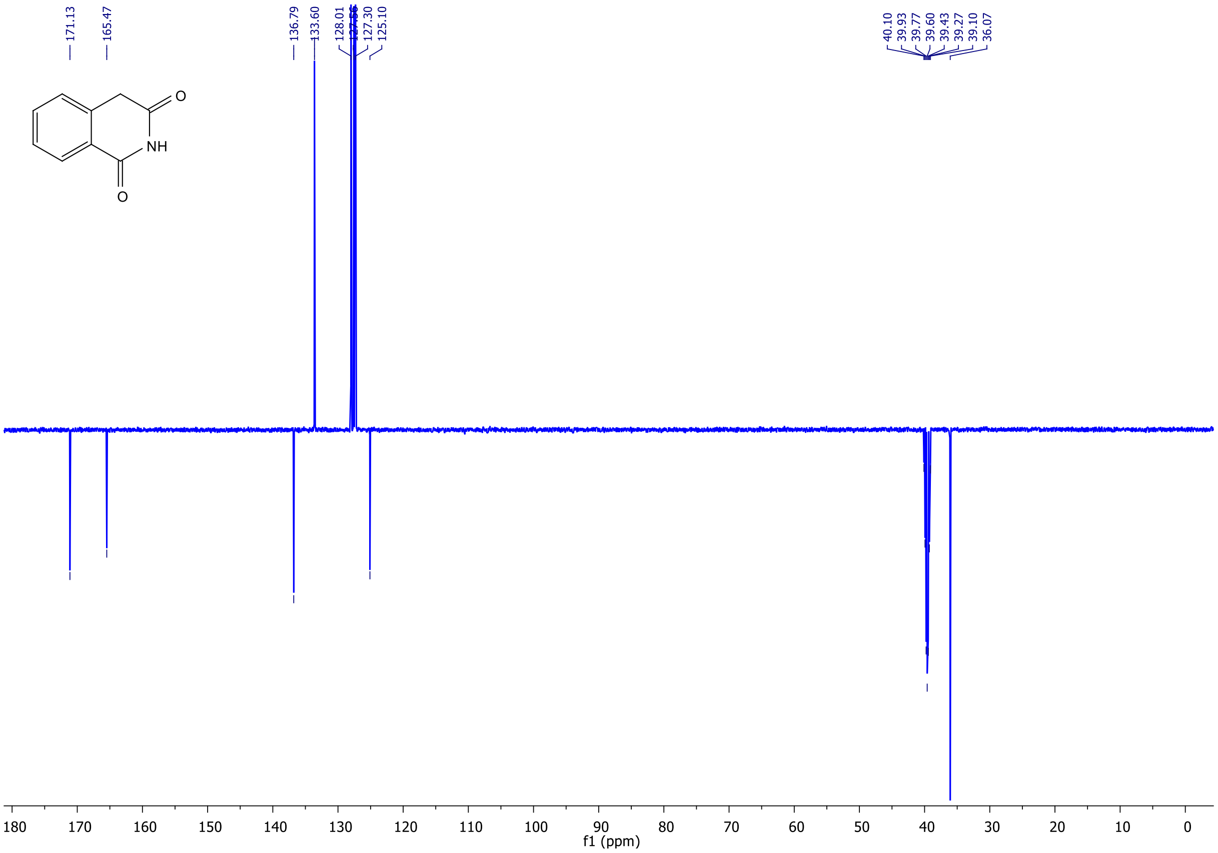

171.13
165.47
136.79
133.60
128.01
127.30
125.10
40.10
39.93
39.77
39.60
39.43
39.27
39.10
36.07
O
NH
O
f1 (ppm)

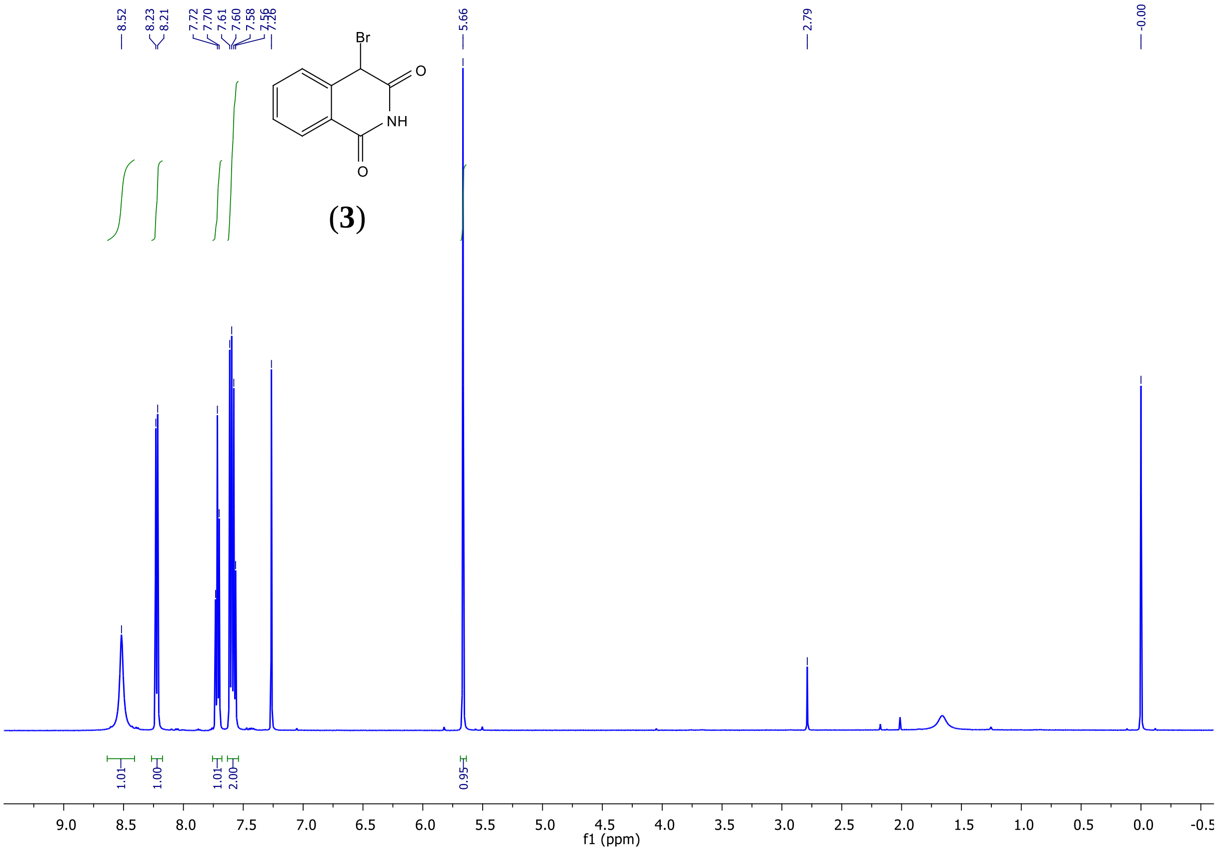

8.52
8.23
8.21
7.72
7.70
7.61
7.60
7.58
7.56
7.26
5.66
2.79
-0.00
Br
O
NH
O
1.01
1.00
1.01
2.00
0.95
f1 (ppm)

(3)

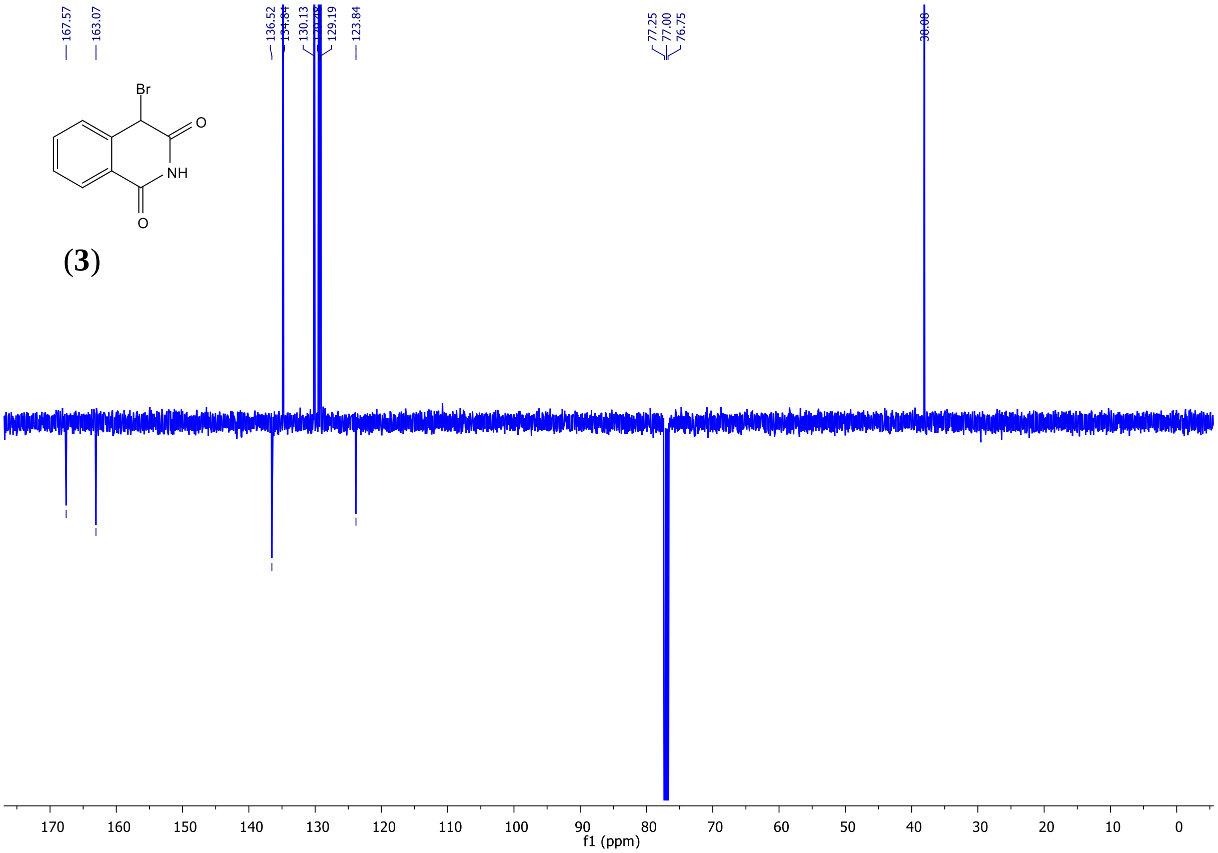

(3)

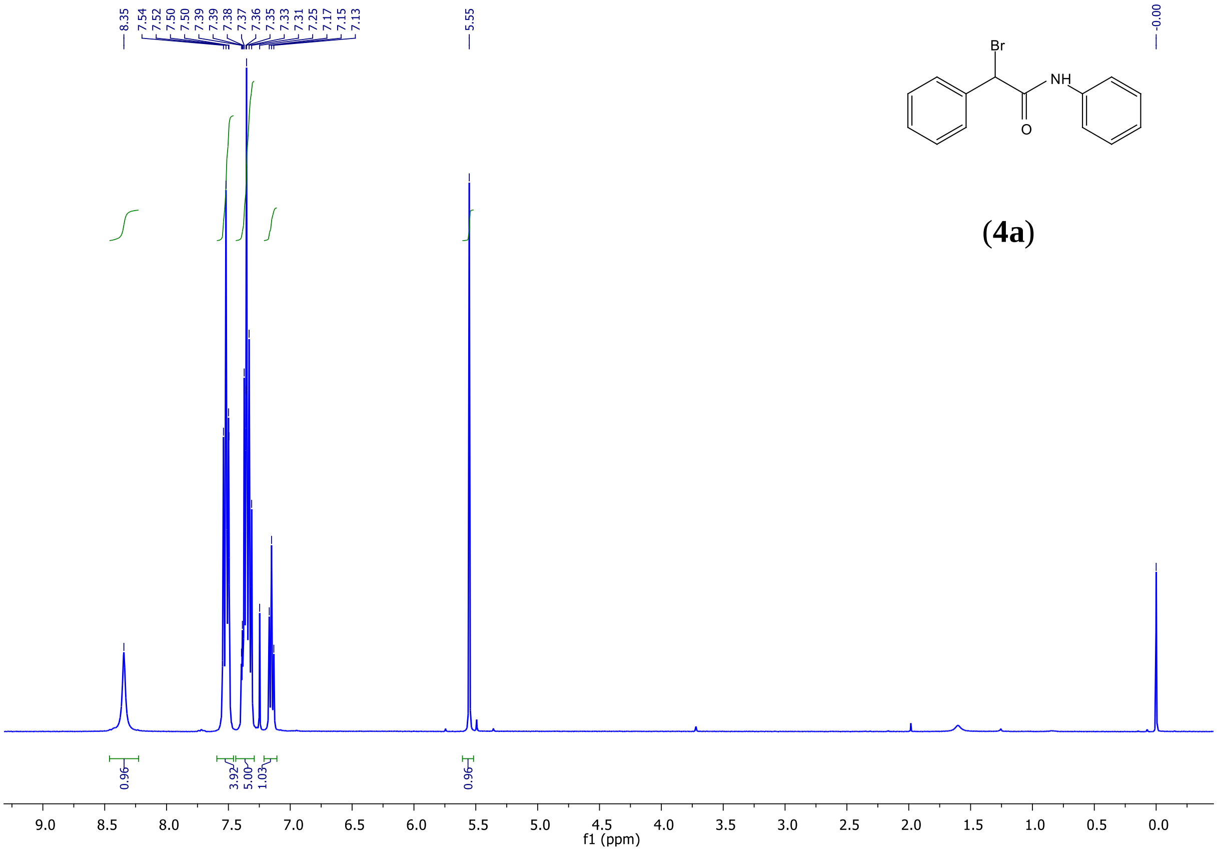

(4a)

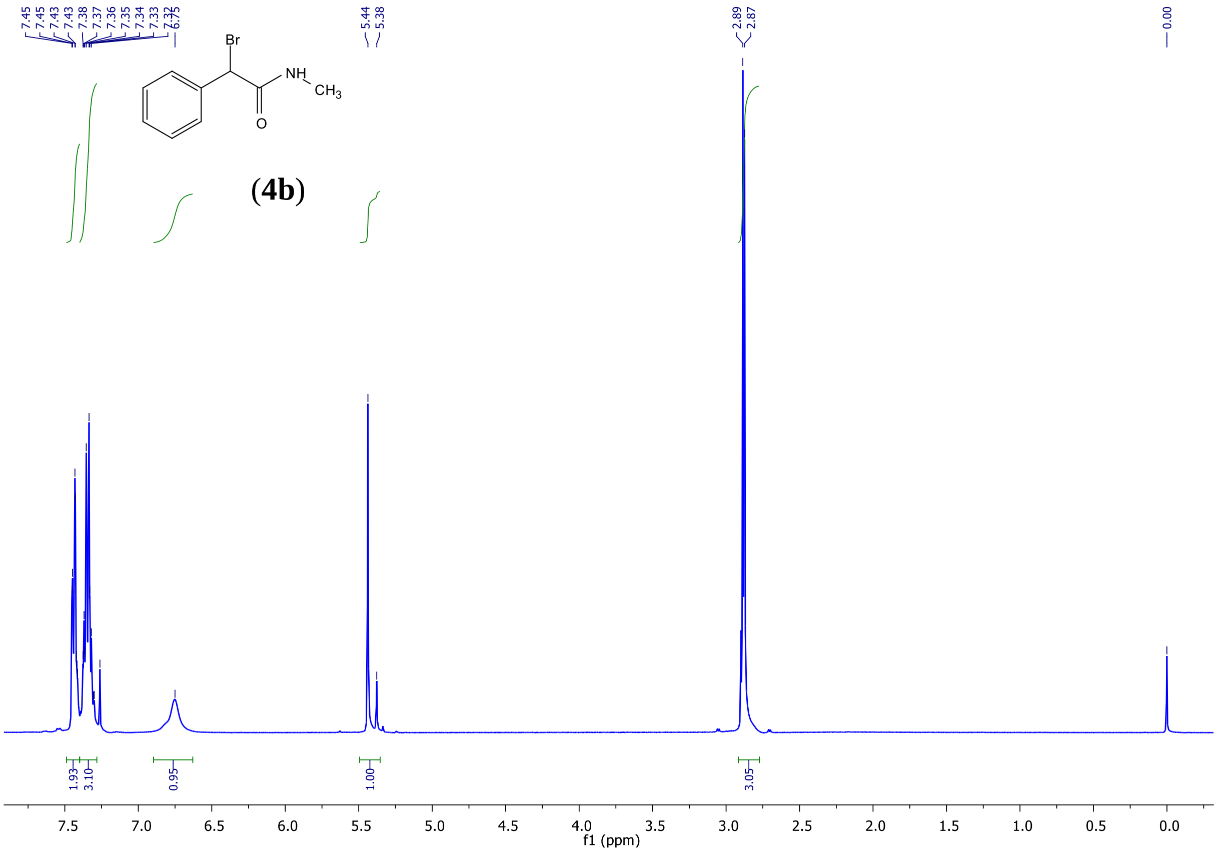

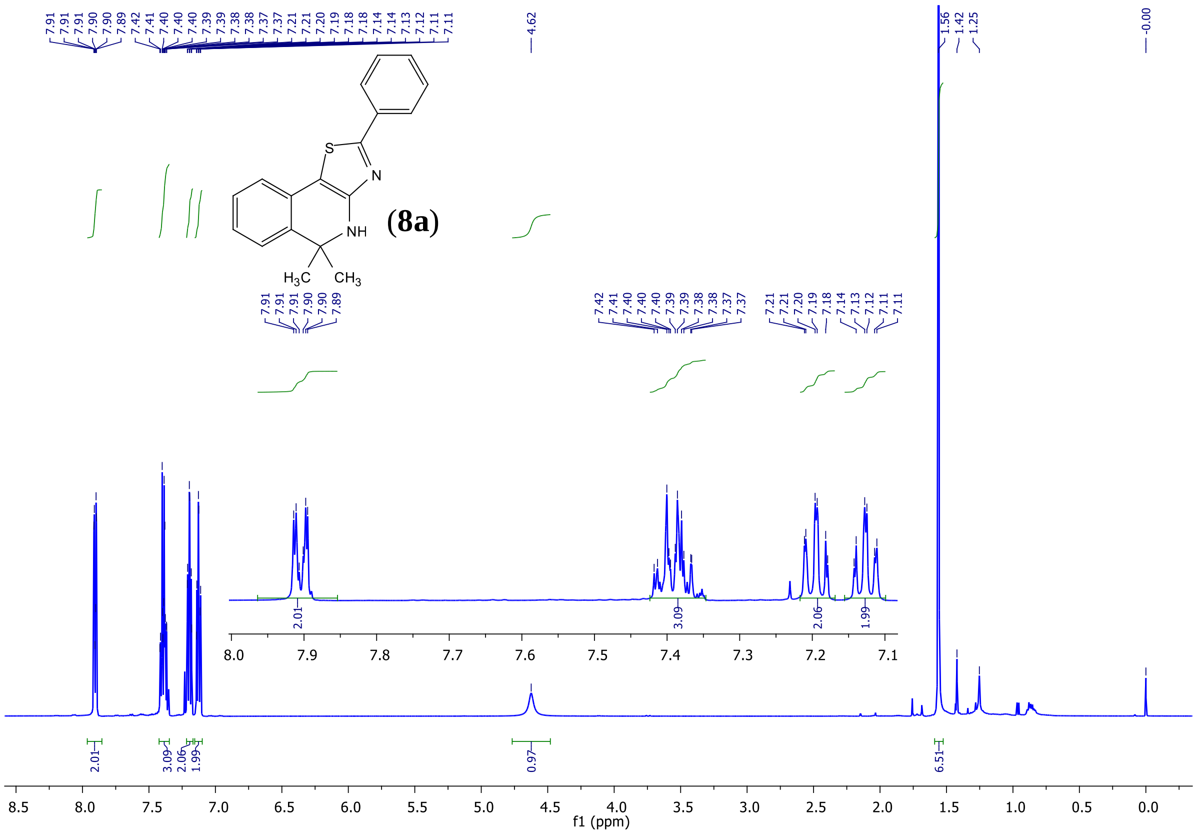

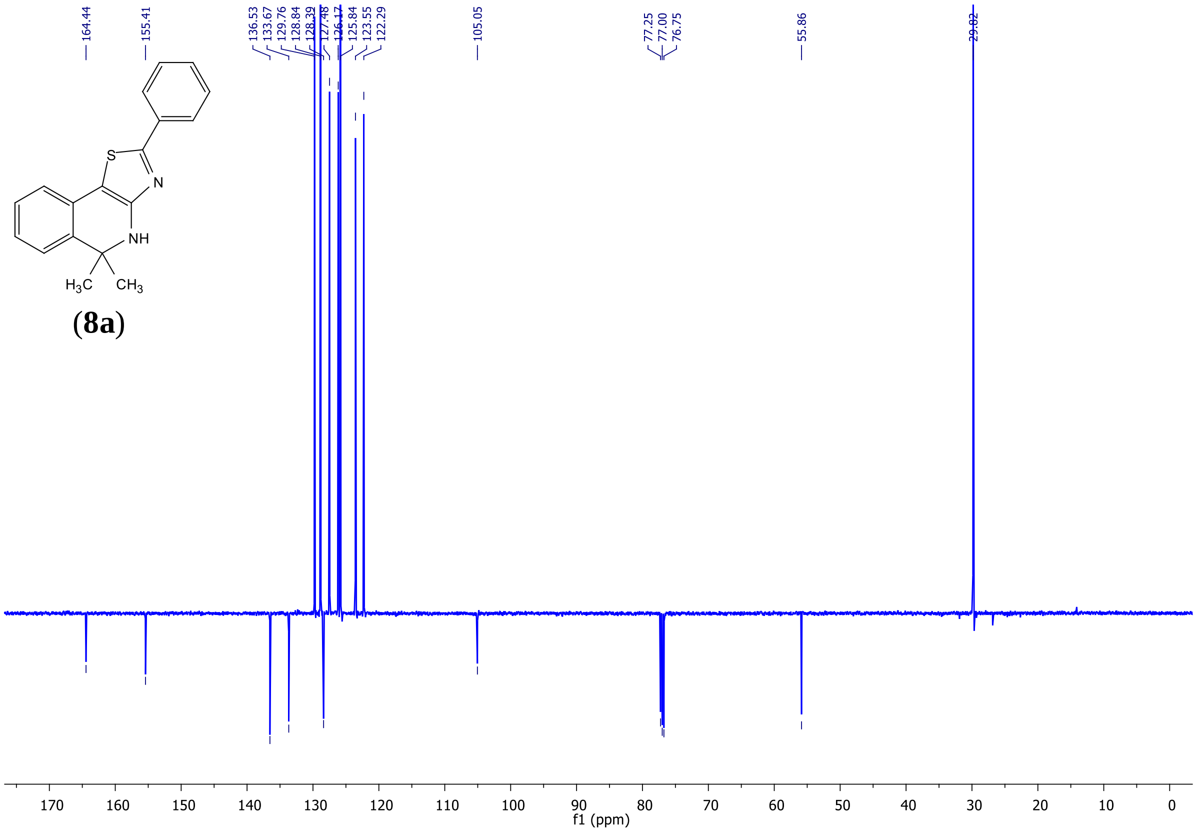

(8a)

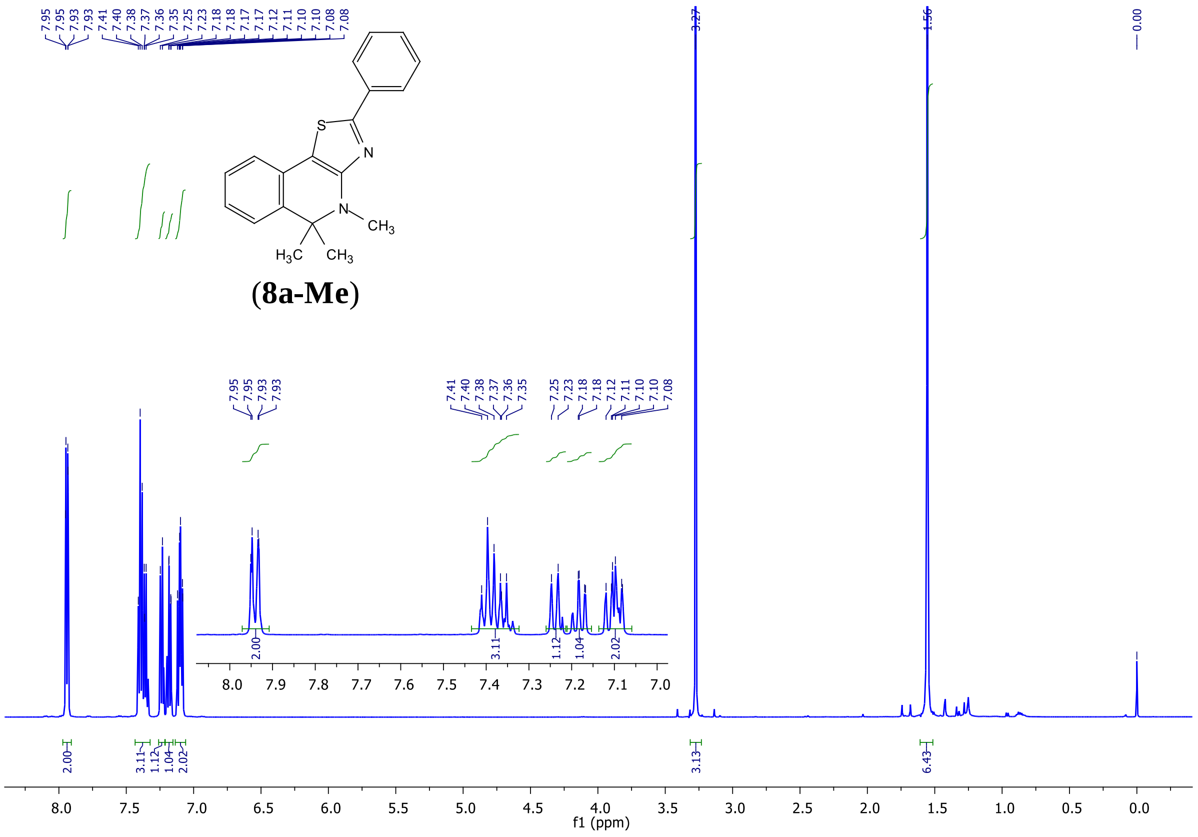

(8a-Me)

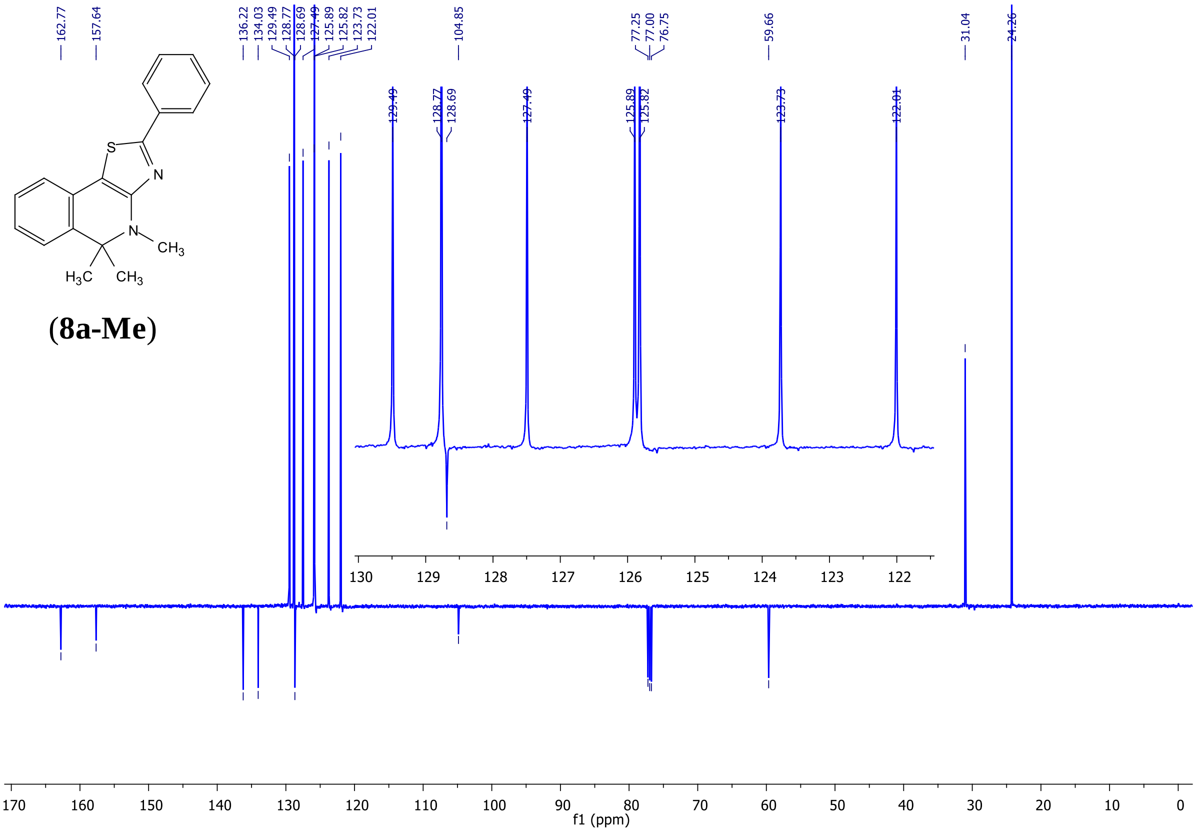
162.77
157.64
136.22
134.03
129.49
128.77
128.69
127.49
125.89
125.82
123.73
122.01
104.85
77.25
77.00
76.75
59.66
31.04
24.26
f1 (ppm)

(8a-Me)

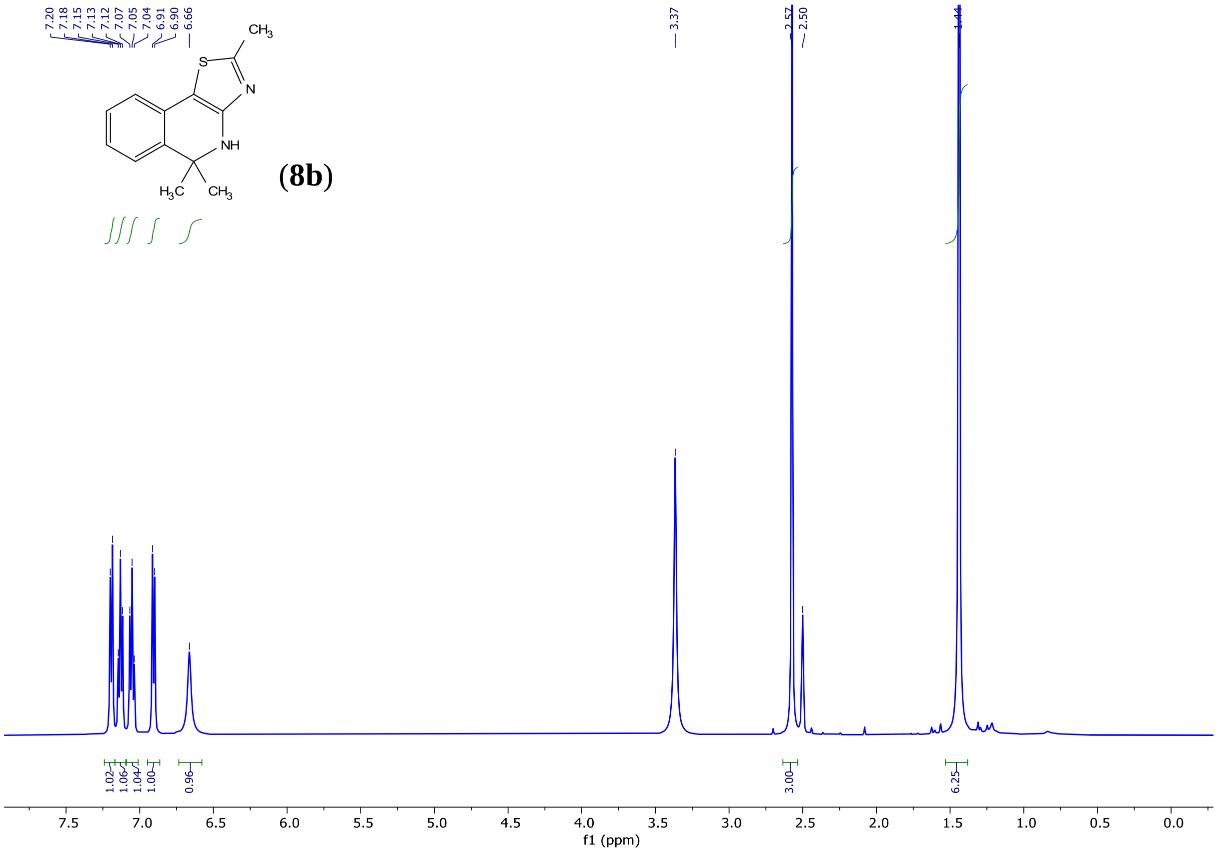
7.20
7.18
7.15
7.13
7.12
7.07
7.05
7.04
6.91
6.90
6.66
3.37
2.57
2.50
1.44
1.02
1.06
1.04
1.00
0.96
3.00
6.25
f1 (ppm)

(8b)

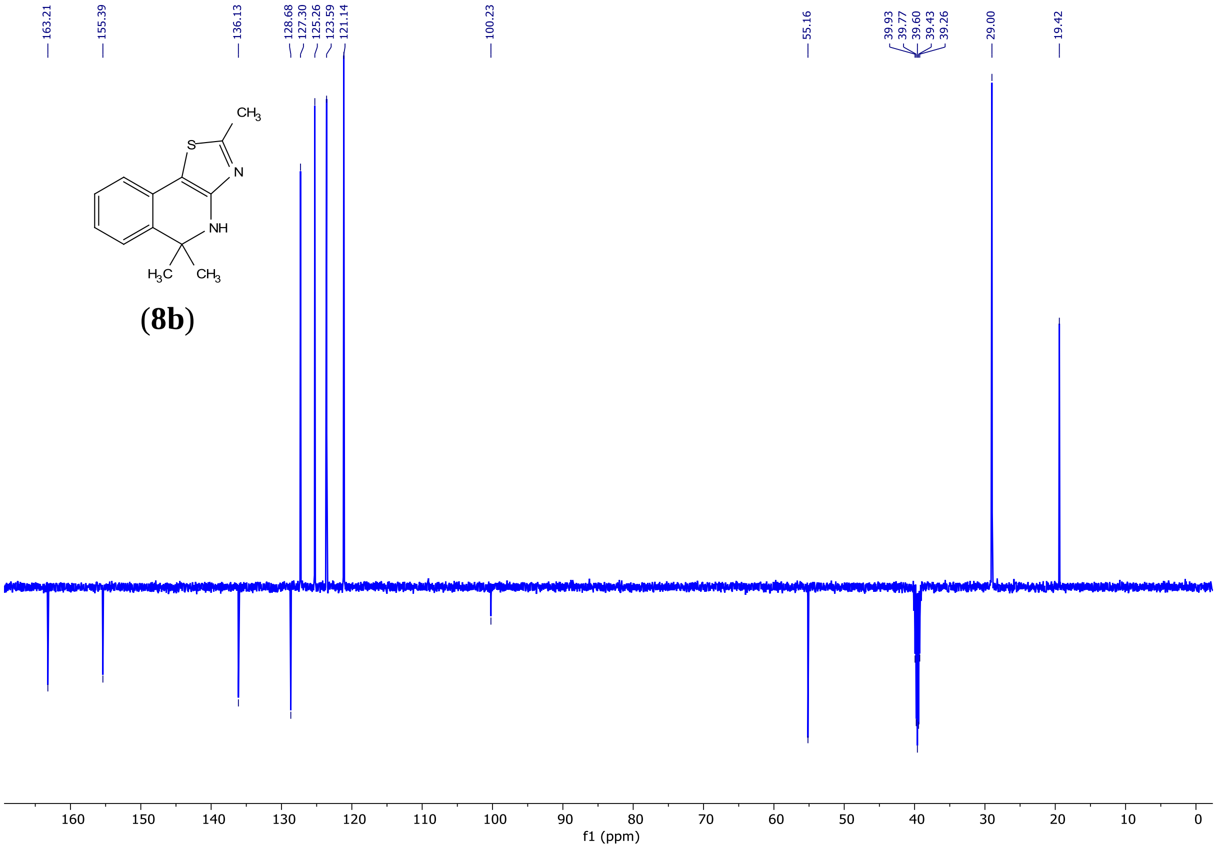
163.21
155.39
136.13
128.68
127.30
125.26
123.59
121.14
100.23
55.16
39.93
39.77
39.60
39.43
39.26
29.00
19.42
CH3
S
N
NH
H3C
CH3
160 150 140 130 120 110 100 90 80 70 60 50 40 30 20 10 0
f1 (ppm)

(8b)

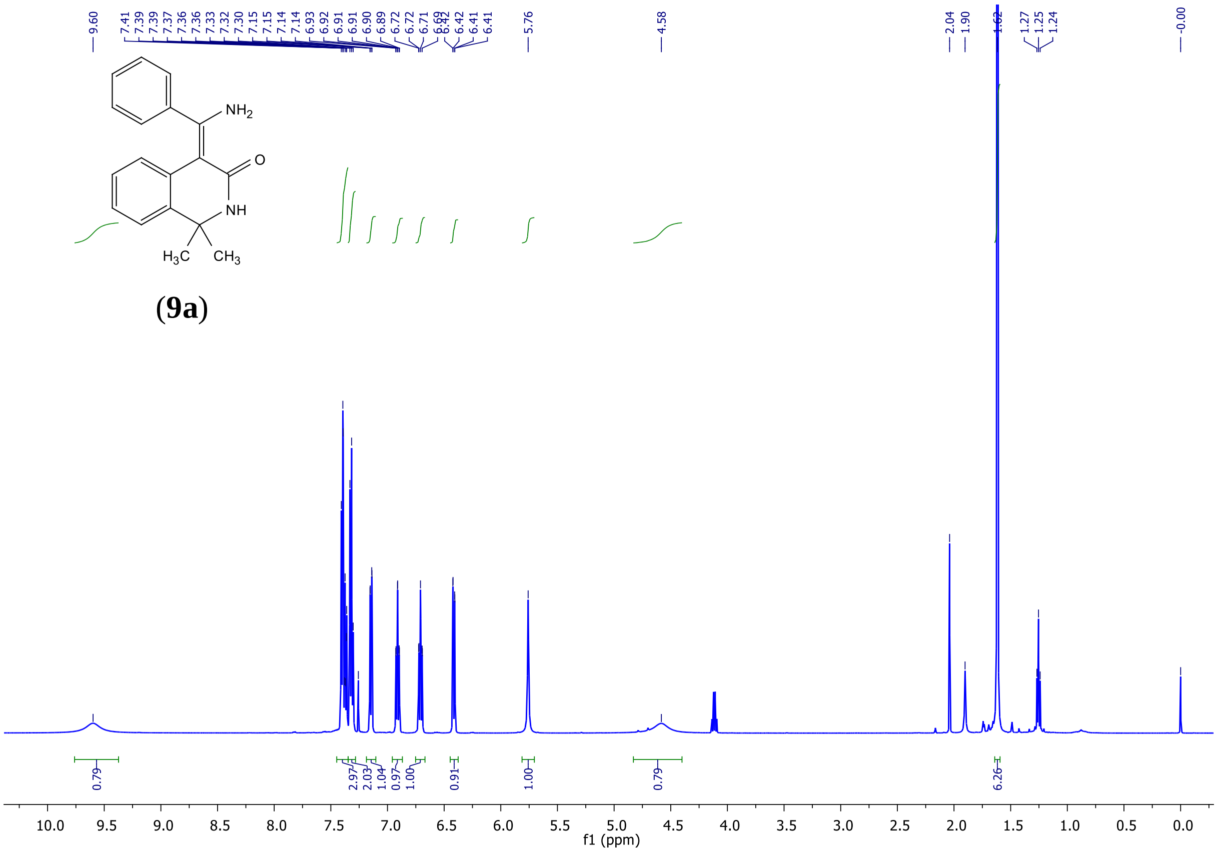
9.60
7.41
7.39
7.39
7.37
7.36
7.36
7.33
7.32
7.30
7.15
7.15
7.14
7.14
6.93
6.92
6.91
6.91
6.90
6.89
6.72
6.72
6.71
6.69
6.42
6.42
6.41
6.41
5.76
4.58
2.04
1.90
1.62
1.27
1.25
1.24
-0.00
NH2
O
NH
H3C
CH3
0.79
2.97
2.03
1.04
0.97
1.00
0.91
1.00
0.79
6.26
10.0 9.5 9.0 8.5 8.0 7.5 7.0 6.5 6.0 5.5 5.0 4.5 4.0 3.5 3.0 2.5 2.0 1.5 1.0 0.5 0.0
f1 (ppm)

(9a)

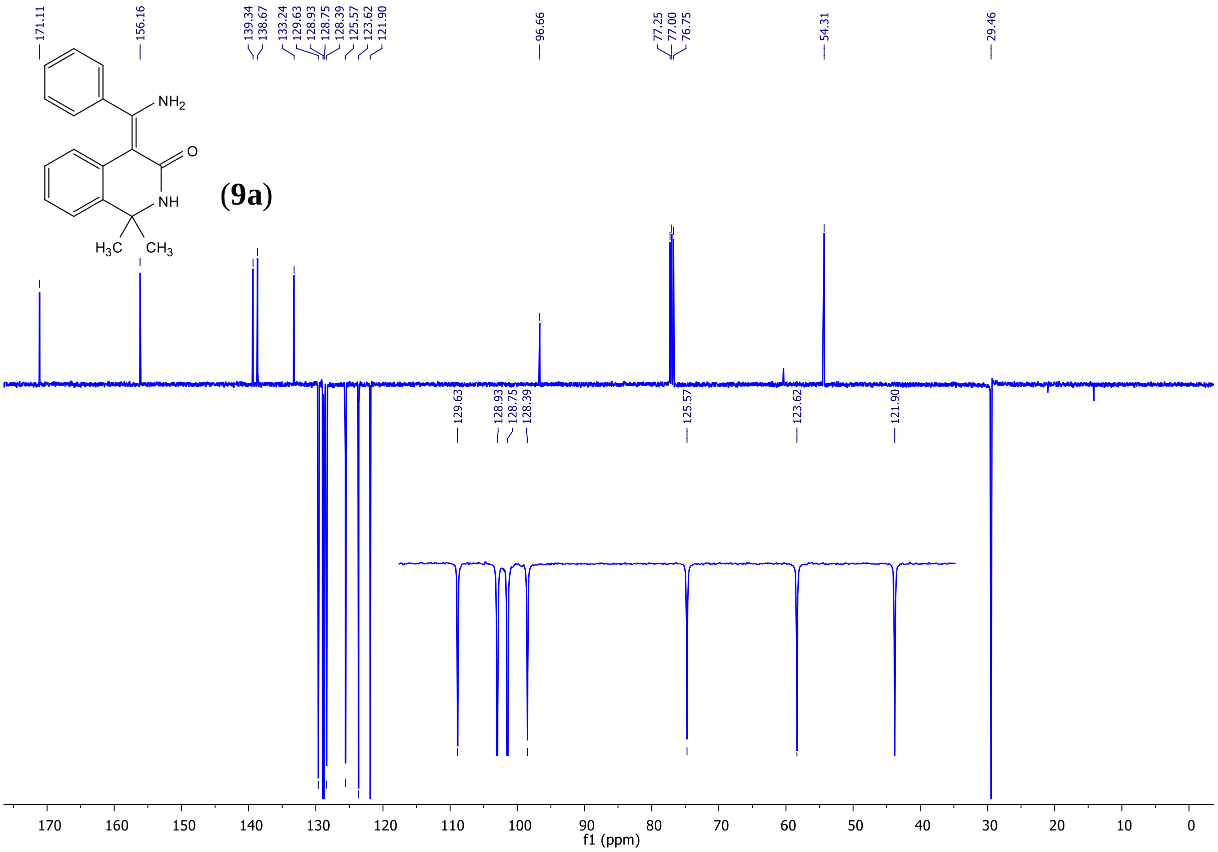

(9a)

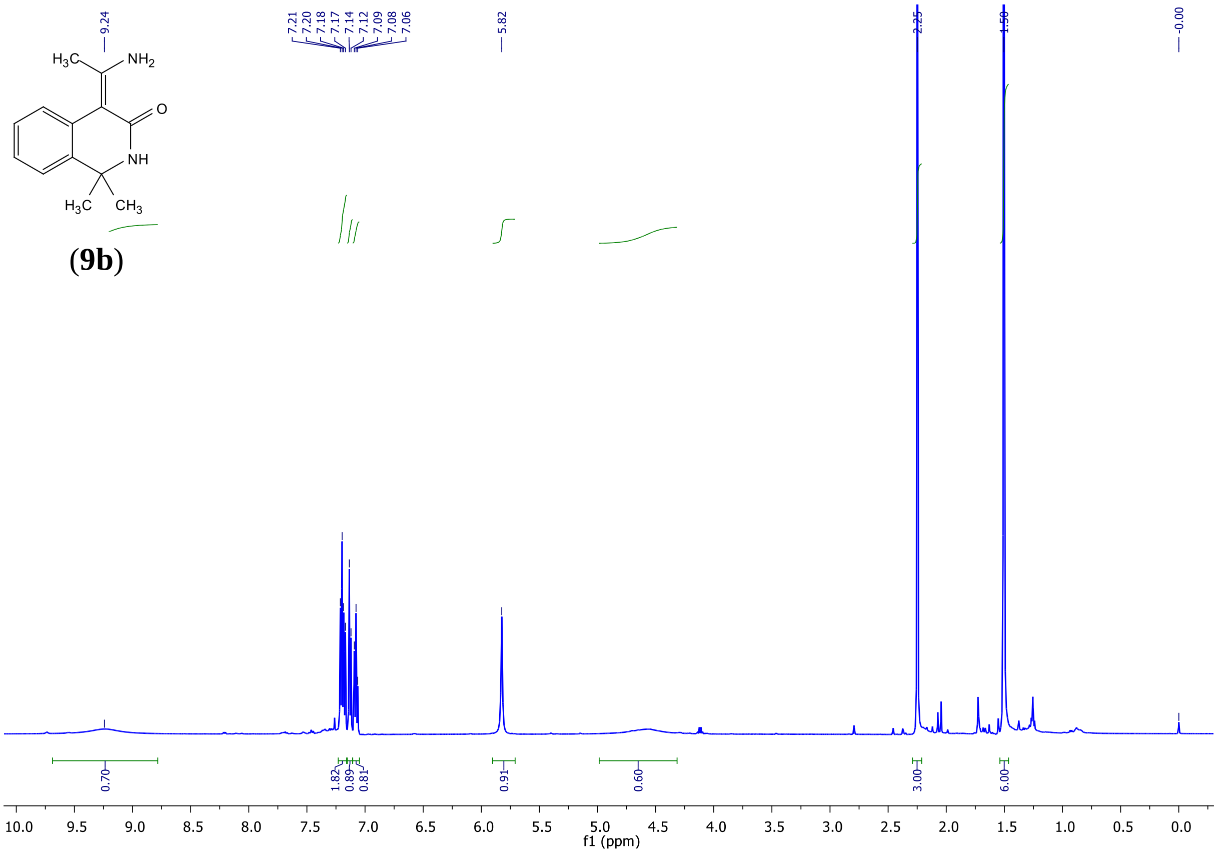

(9b)

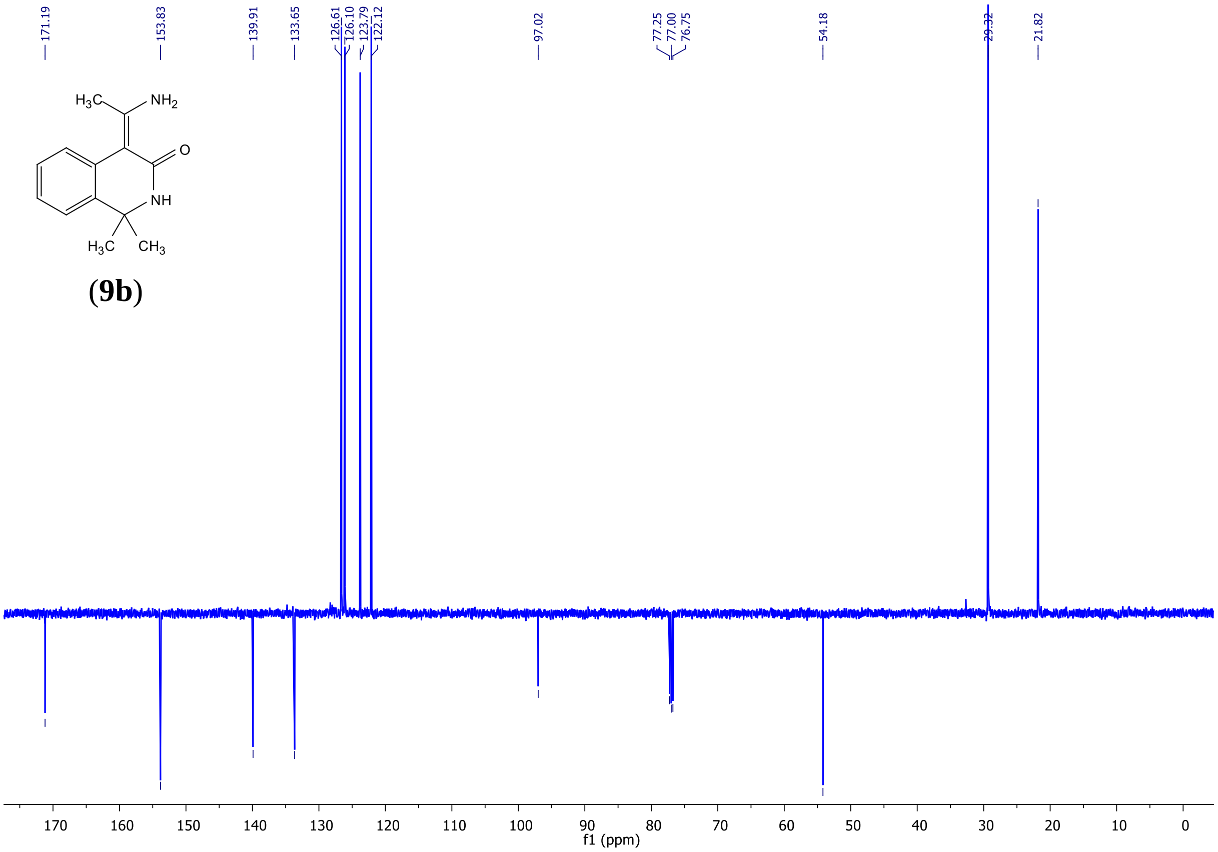

(9b)

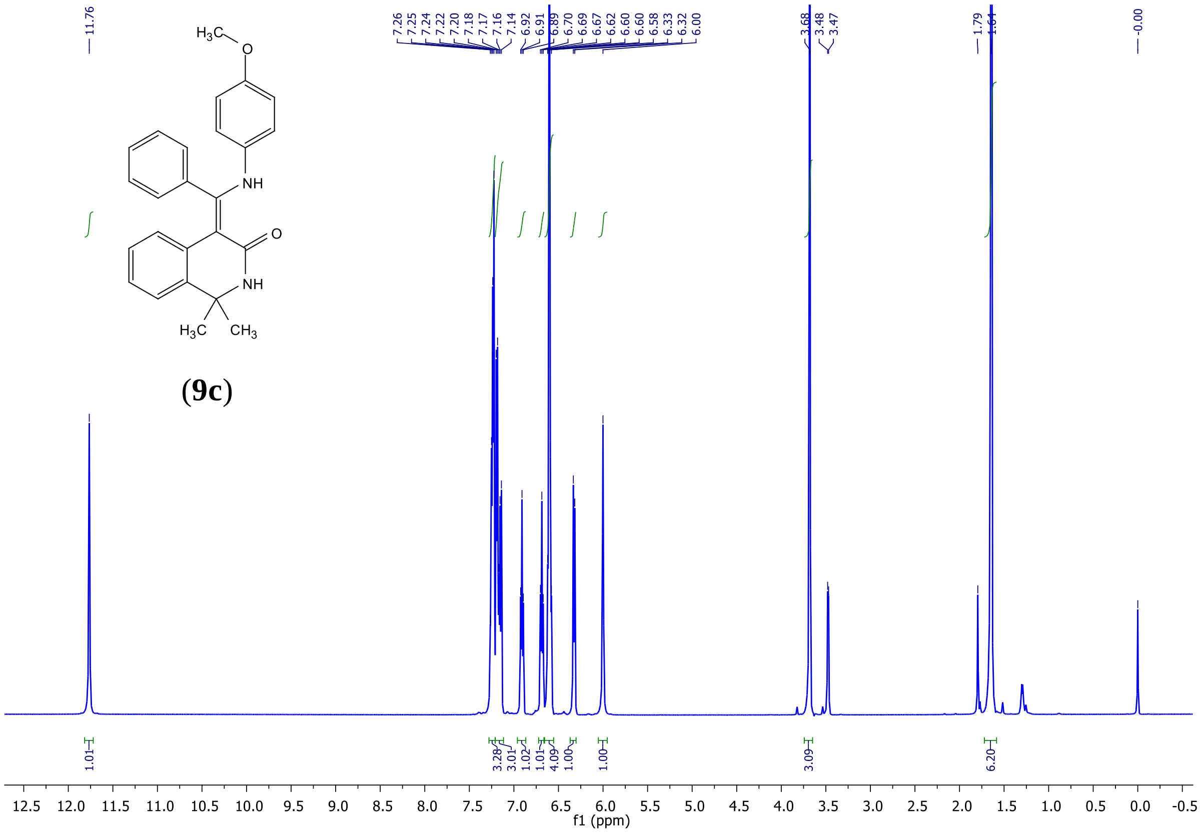

(9c)

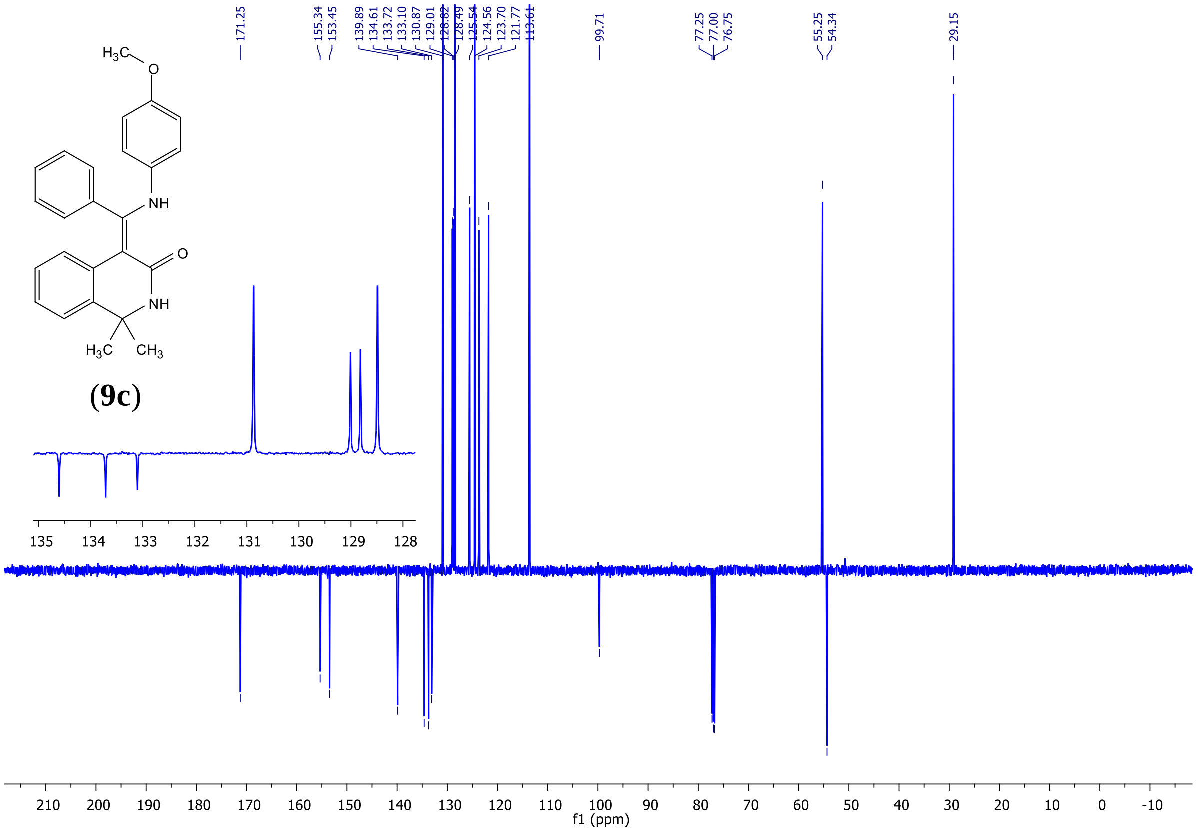

(**9c**)

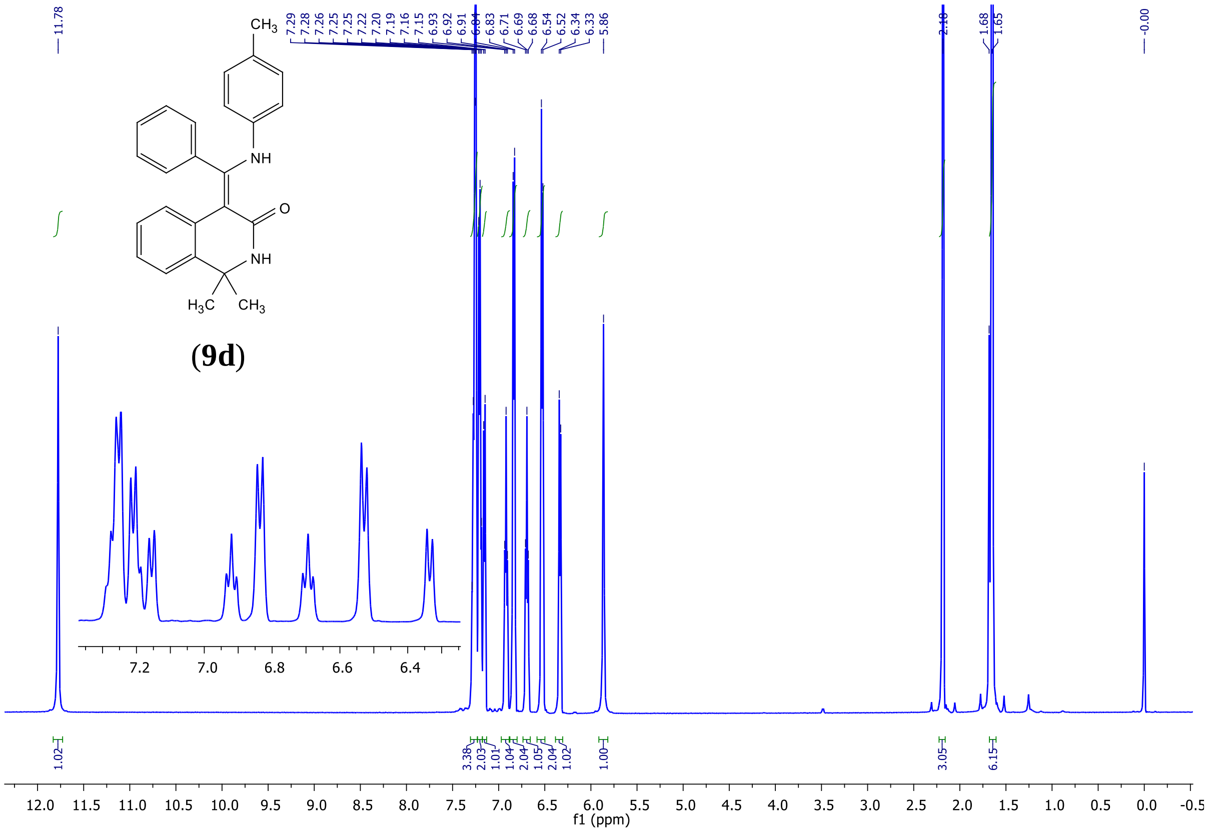

(**9d**)

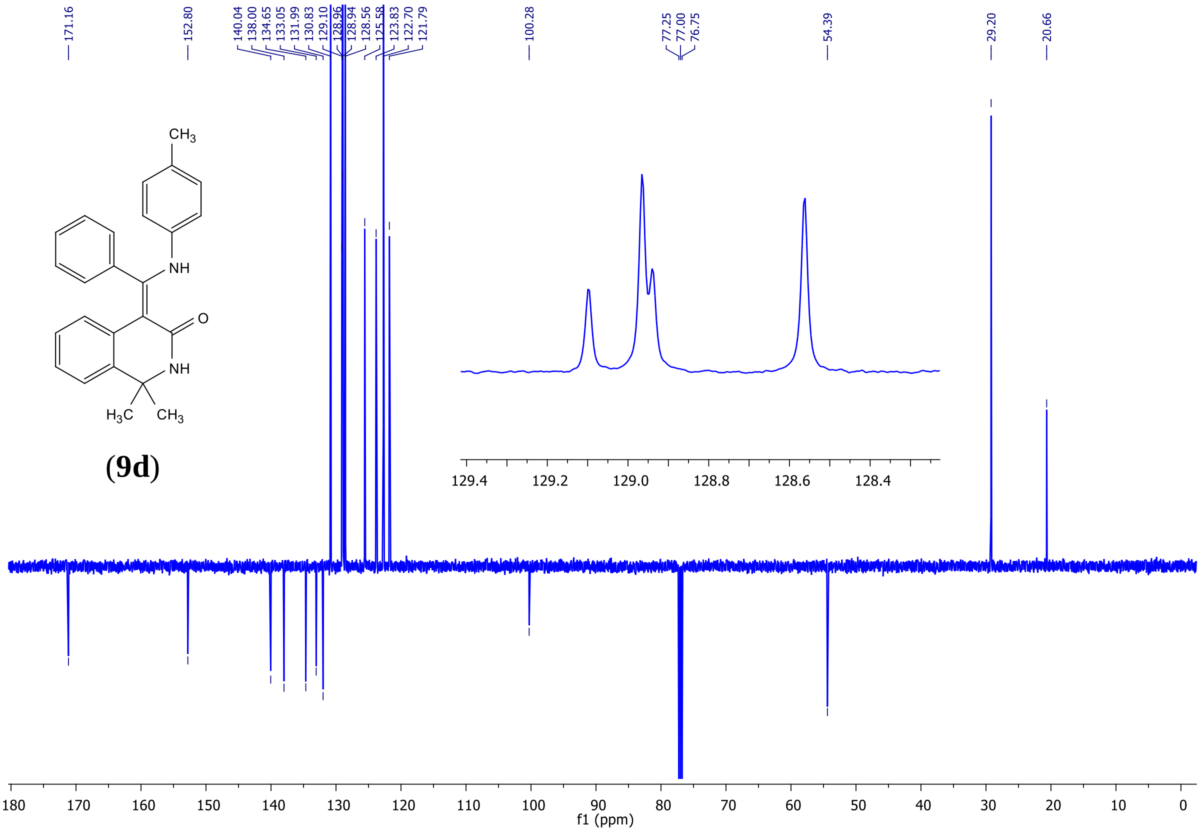

(9d)

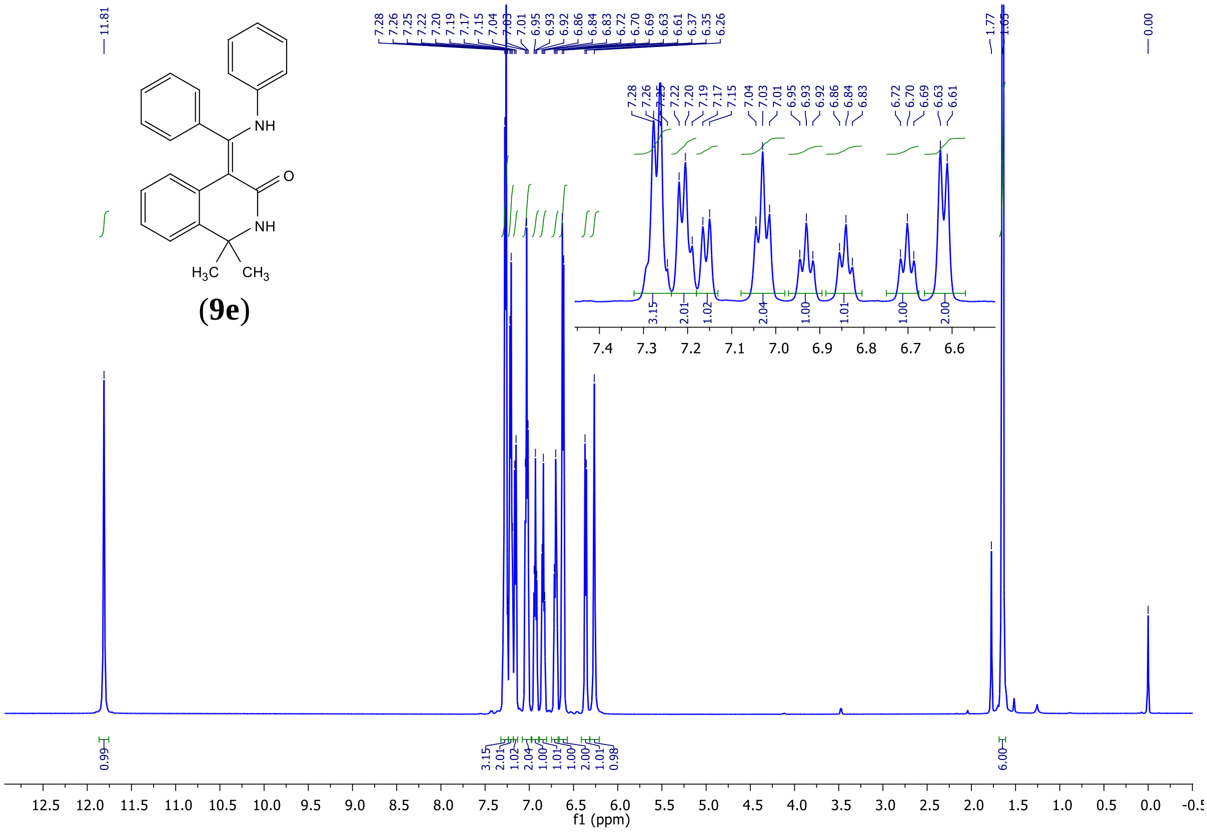

(9e)

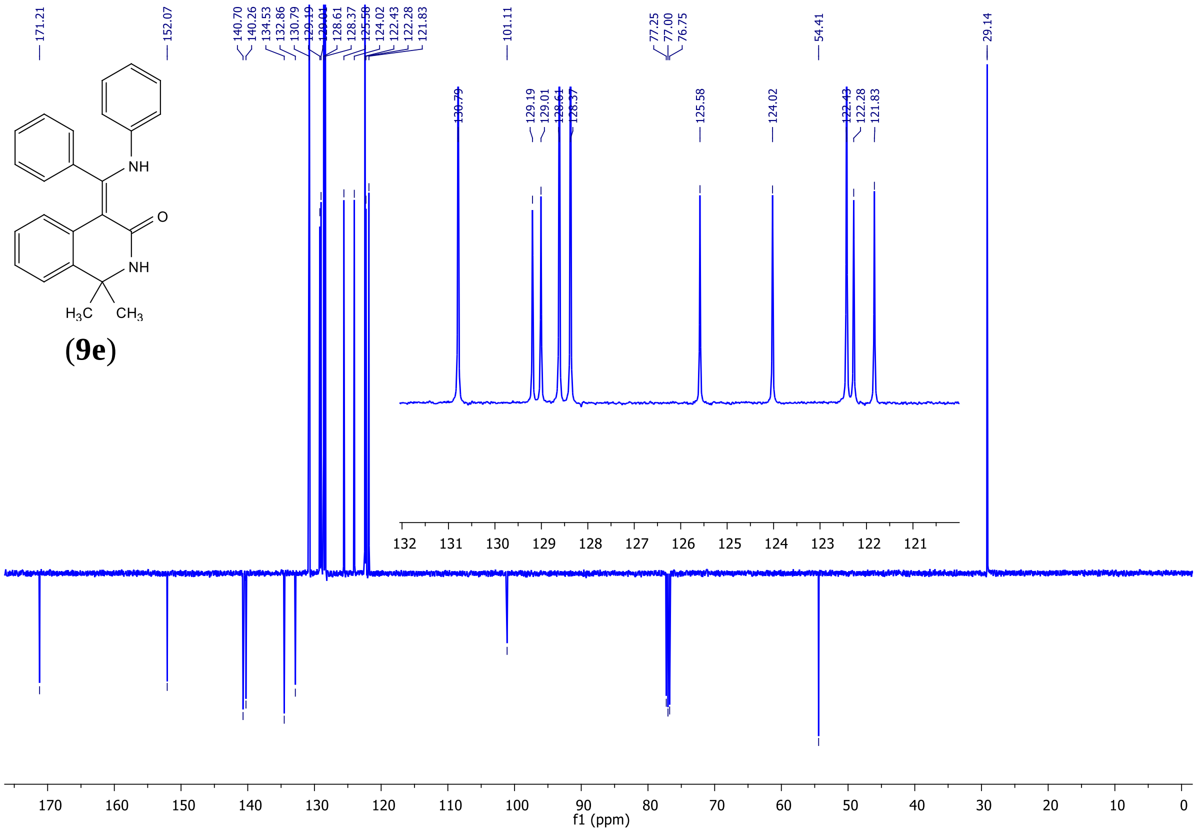
171.21
152.07
140.70
140.26
134.53
132.86
130.79
129.19
129.01
128.61
128.37
125.58
124.02
122.43
122.28
121.83
101.11
77.25
77.00
76.75
54.41
29.14
NH
O
NH
H3C
CH3
f1 (ppm)

(9e)

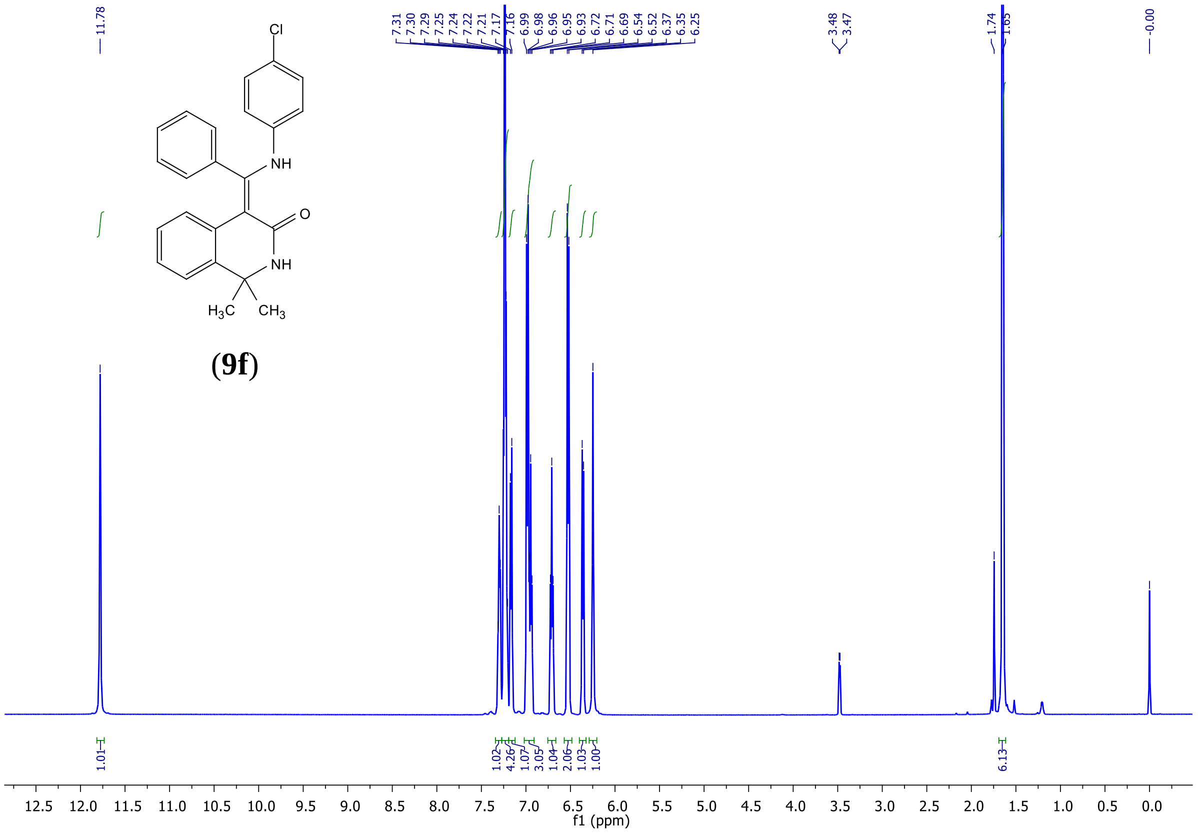
11.78
7.31
7.30
7.29
7.25
7.24
7.22
7.21
7.17
7.16
6.99
6.98
6.96
6.95
6.93
6.72
6.71
6.69
6.54
6.52
6.37
6.35
6.25
3.48
3.47
1.74
1.65
-0.00
Cl
NH
O
NH
H3C
CH3
1.01
1.02
4.26
1.07
3.05
1.04
2.06
1.03
1.00
6.13
f1 (ppm)

(9f)

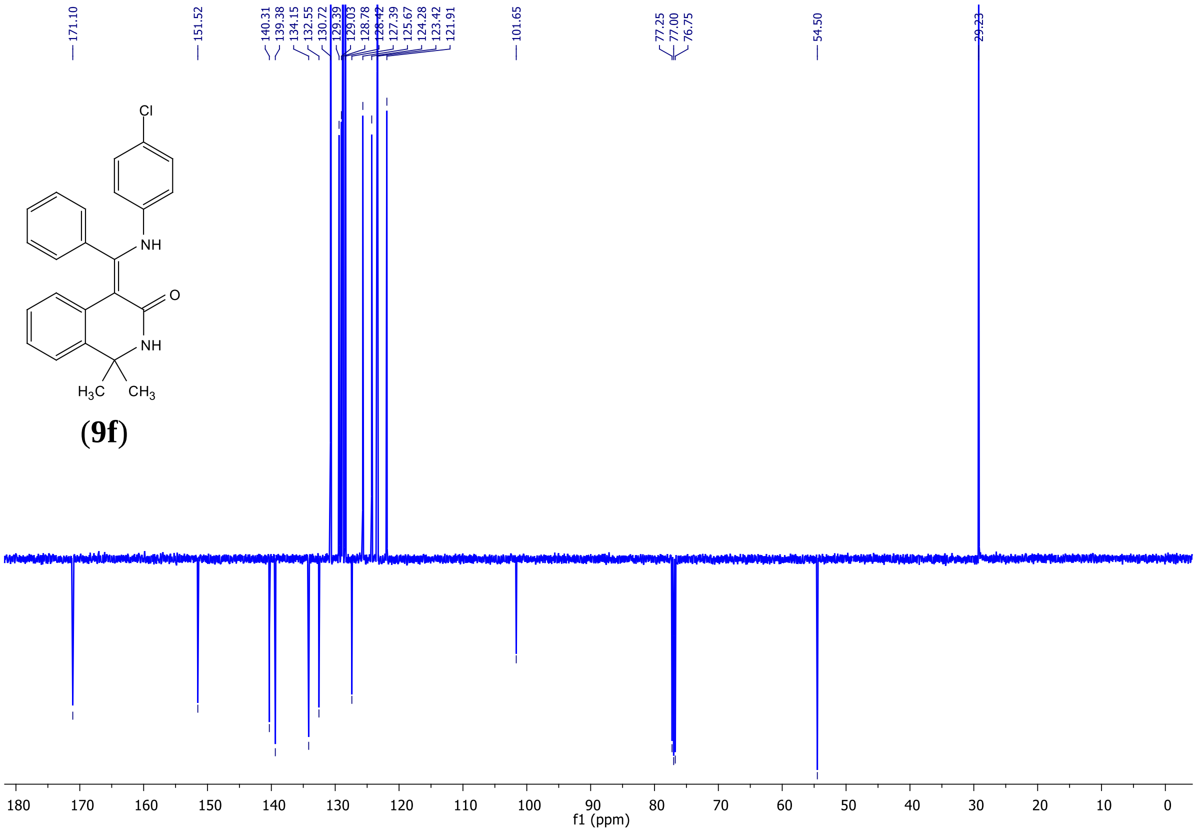

171.10
151.52
140.31
139.38
134.15
132.55
130.72
129.39
129.03
128.78
127.39
125.67
124.28
123.42
121.91
101.65
77.25
77.00
76.75
54.50
29.23
Cl
NH
O
NH
H_3C
CH_3
f1 (ppm)

(9f)

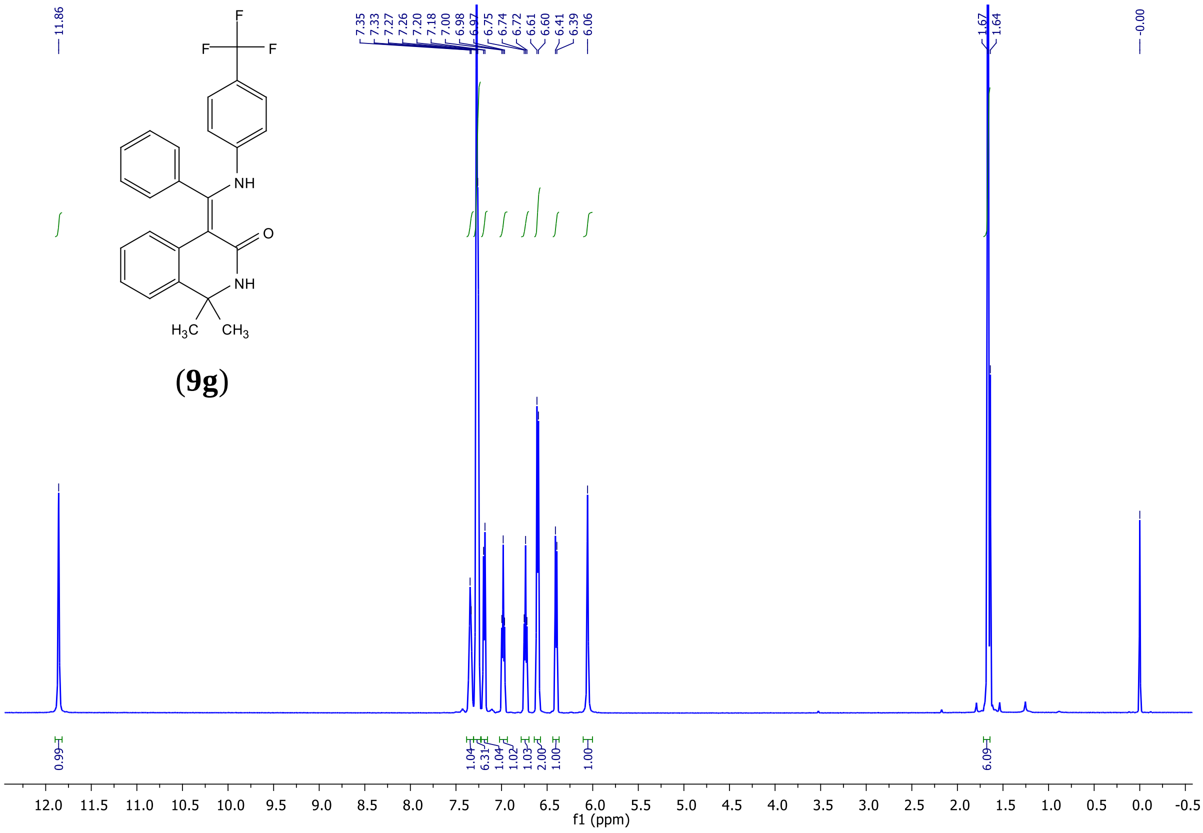

11.86
7.35
7.33
7.27
7.26
7.20
7.18
7.00
6.98
6.97
6.75
6.74
6.72
6.61
6.60
6.41
6.39
6.06
1.67
1.64
-0.00
F
F
F
NH
O
NH
H_3C
CH_3
0.99
1.04
6.31
1.04
1.02
1.03
2.00
1.00
1.00
6.09
f1 (ppm)

(9g)

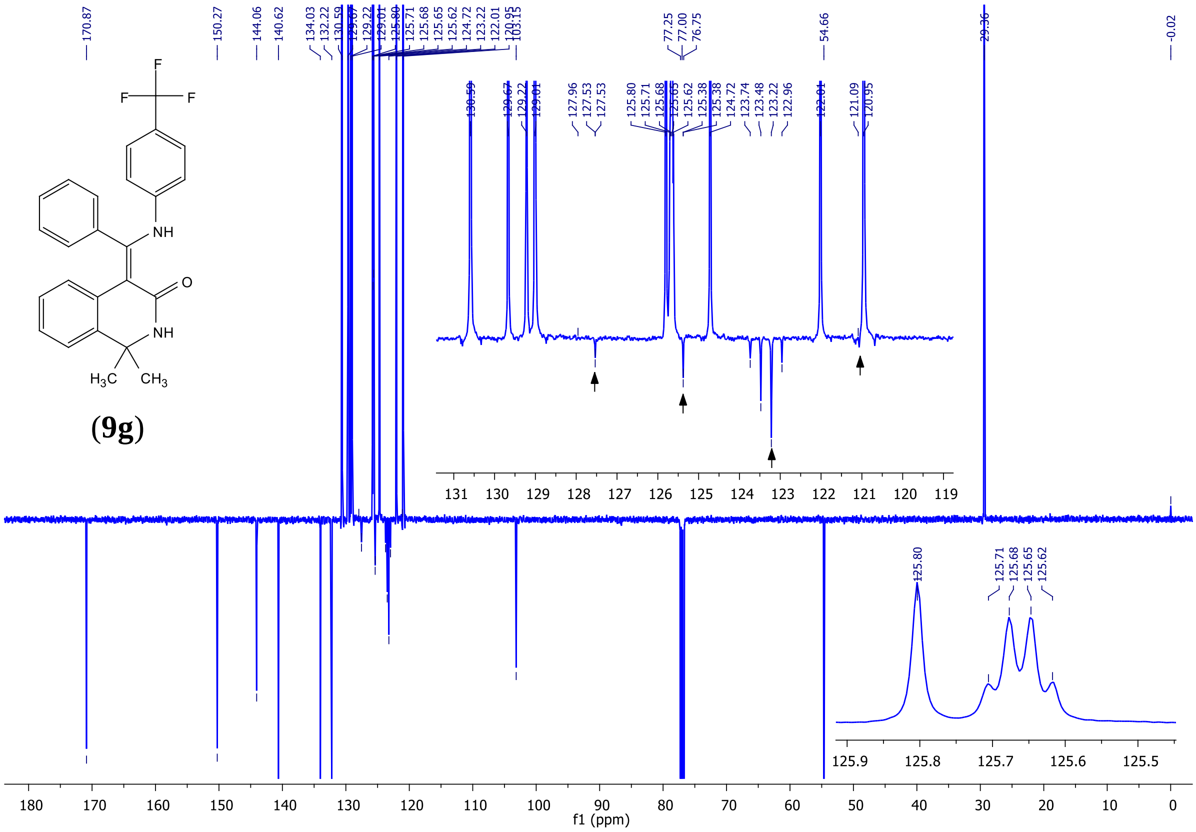

(9g)

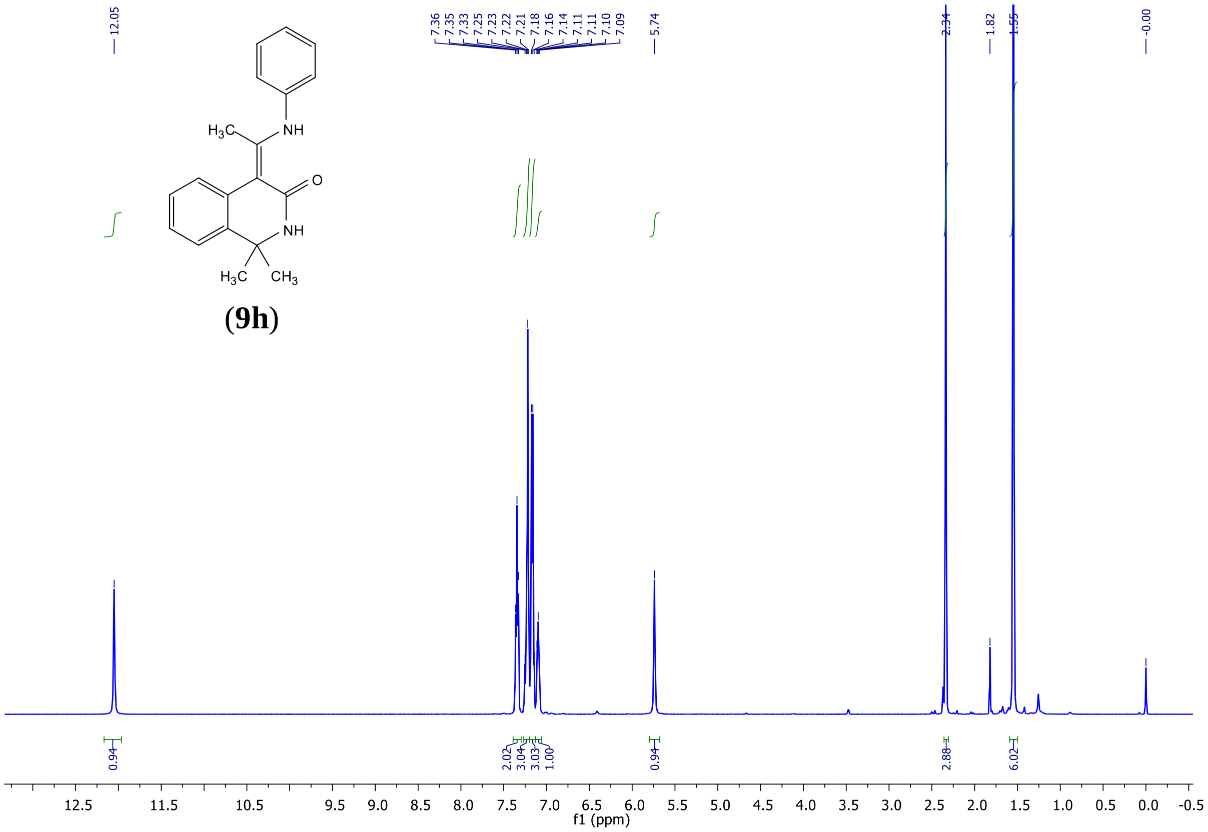

(9h)

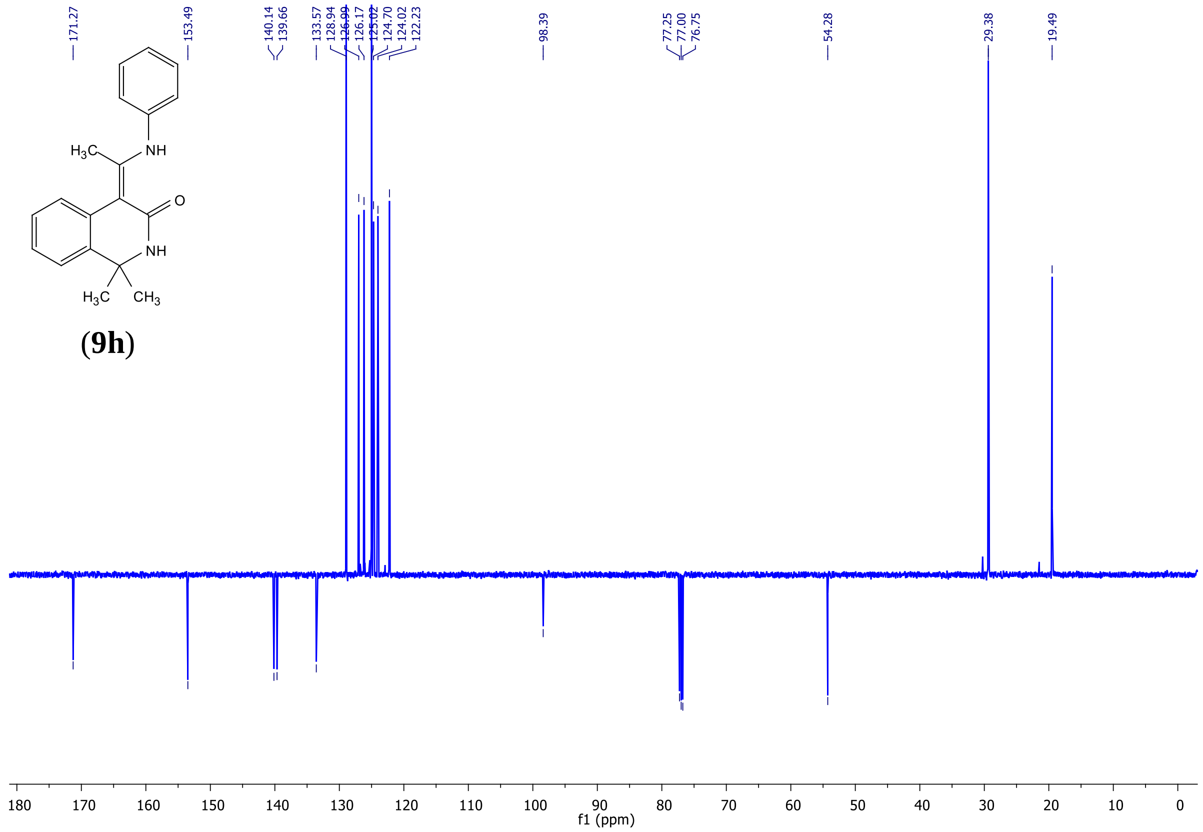

(9h)

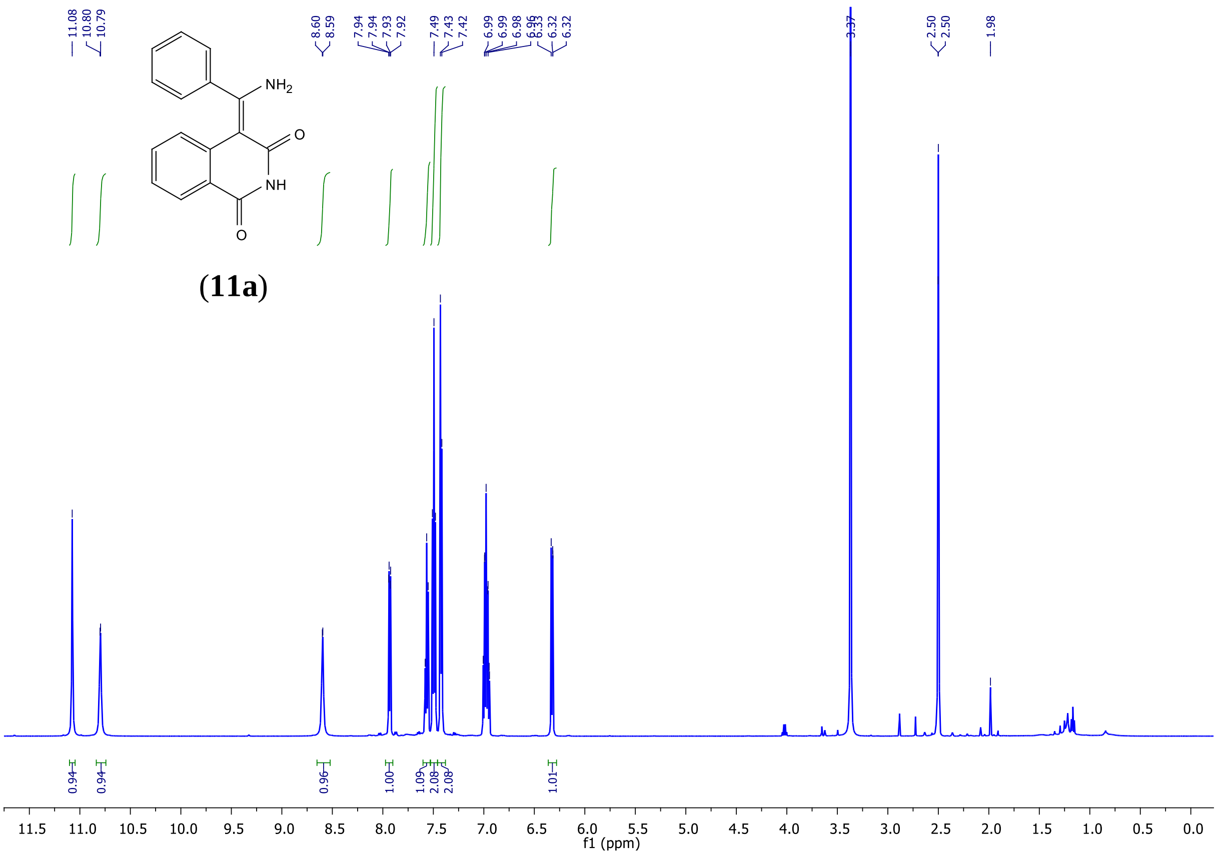

(11a)

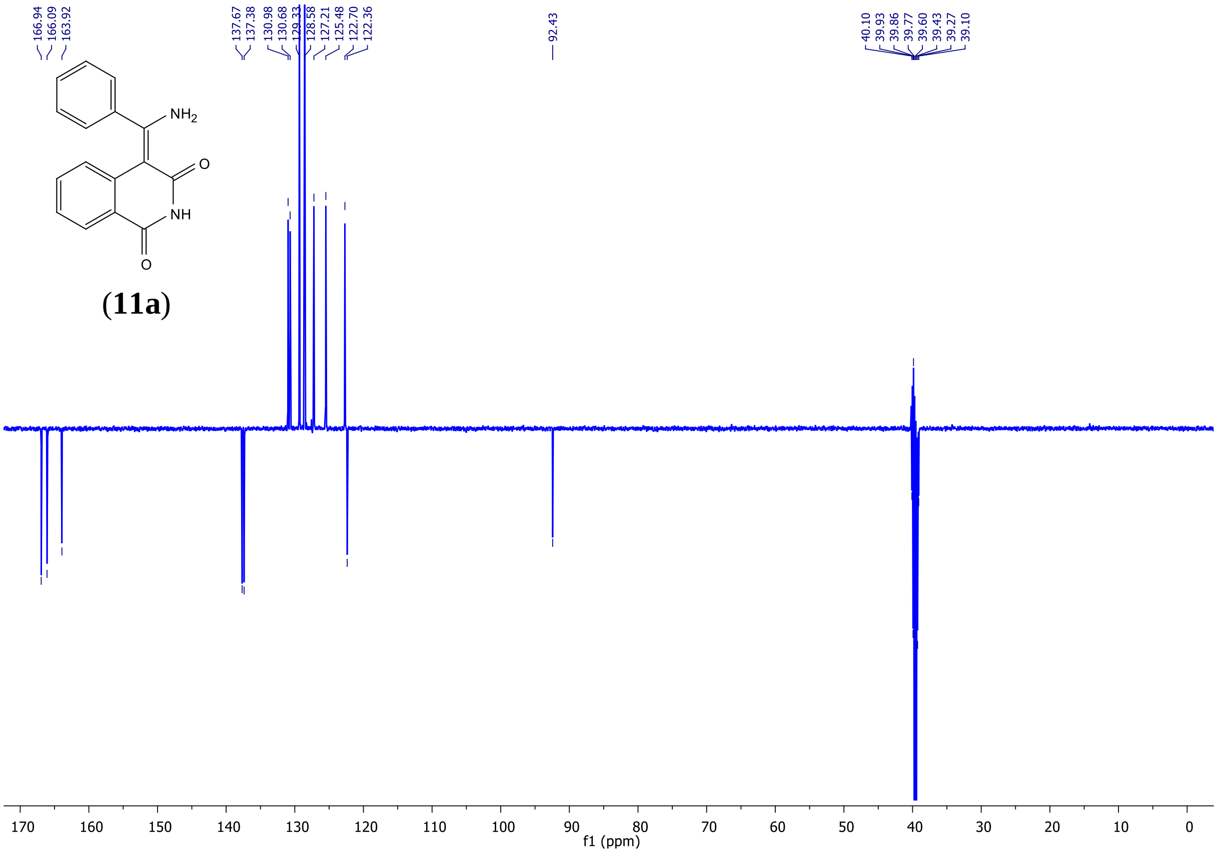

166.94
166.09
163.92
137.67
137.38
130.98
130.68
129.33
128.58
127.21
125.48
122.70
122.36
92.43
40.10
39.93
39.86
39.77
39.60
39.43
39.27
39.10
NH_2
O
NH
O
170 160 150 140 130 120 110 100 90 80 70 60 50 40 30 20 10 0
f1 (ppm)

(11a)

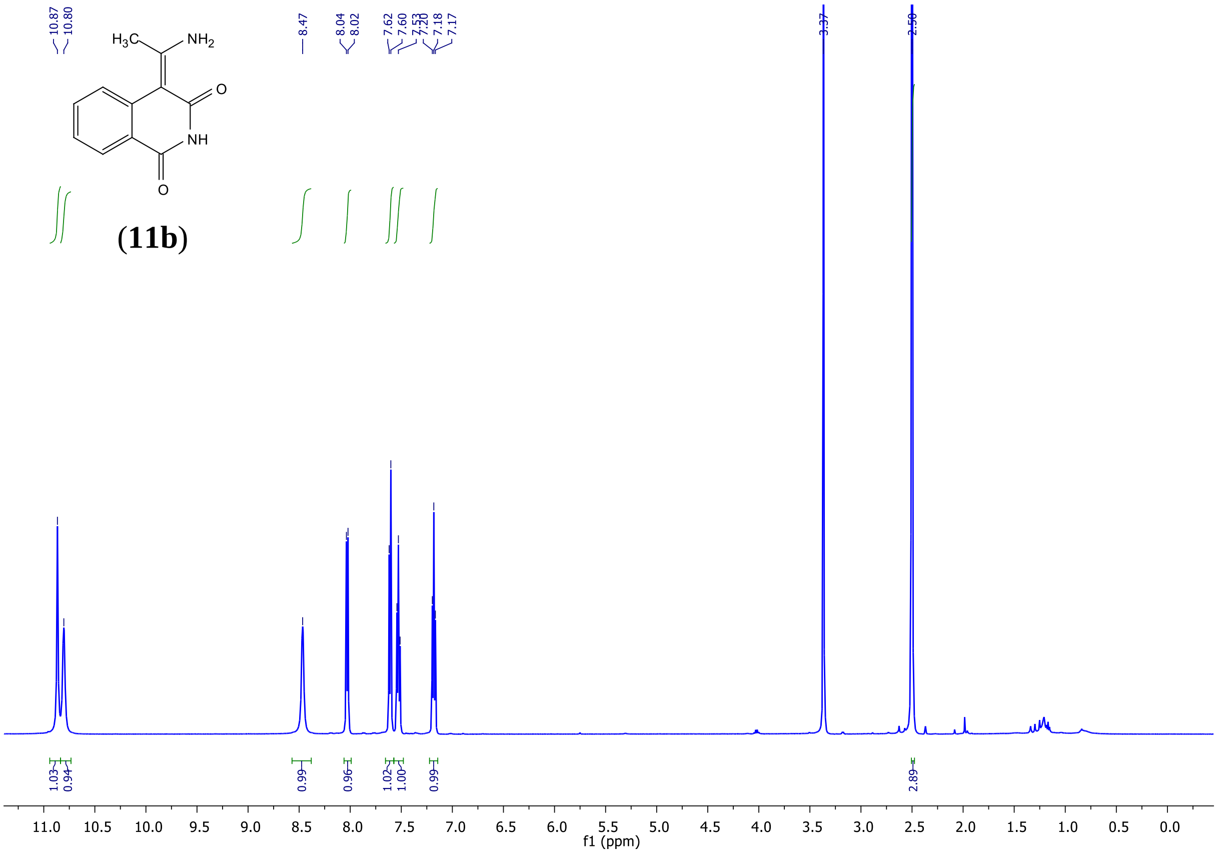

10.87
10.80
8.47
8.04
8.02
7.62
7.60
7.53
7.20
7.18
7.17
3.37
2.50
H_3C
NH_2
O
NH
O
1.03
0.94
0.99
0.96
1.02
1.00
0.99
2.89
11.0 10.5 10.0 9.5 9.0 8.5 8.0 7.5 7.0 6.5 6.0 5.5 5.0 4.5 4.0 3.5 3.0 2.5 2.0 1.5 1.0 0.5 0.0
f1 (ppm)

(11b)

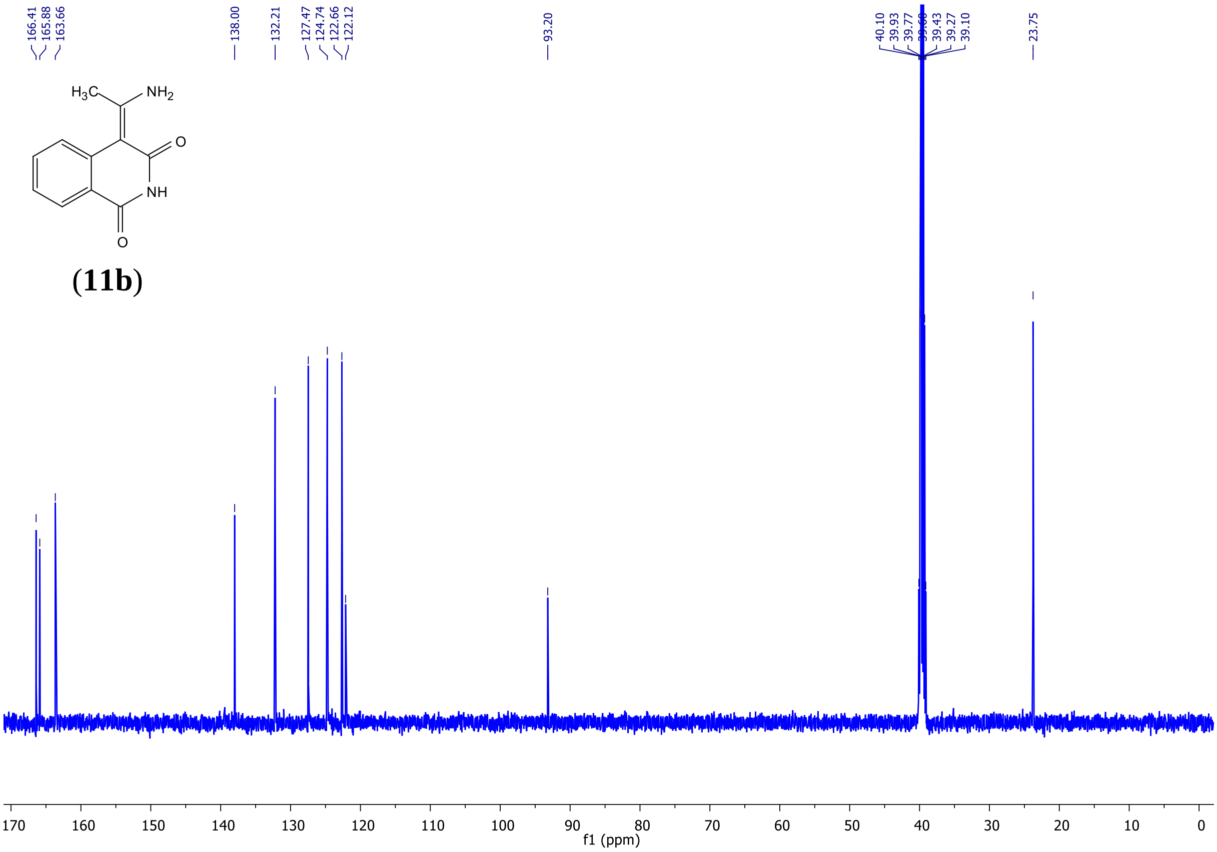
166.41
165.88
163.66
138.00
132.21
127.47
124.74
122.66
122.12
93.20
40.10
39.93
39.77
39.43
39.27
39.10
23.75
H3C
NH2
O
NH
O
(11b)
f1 (ppm)

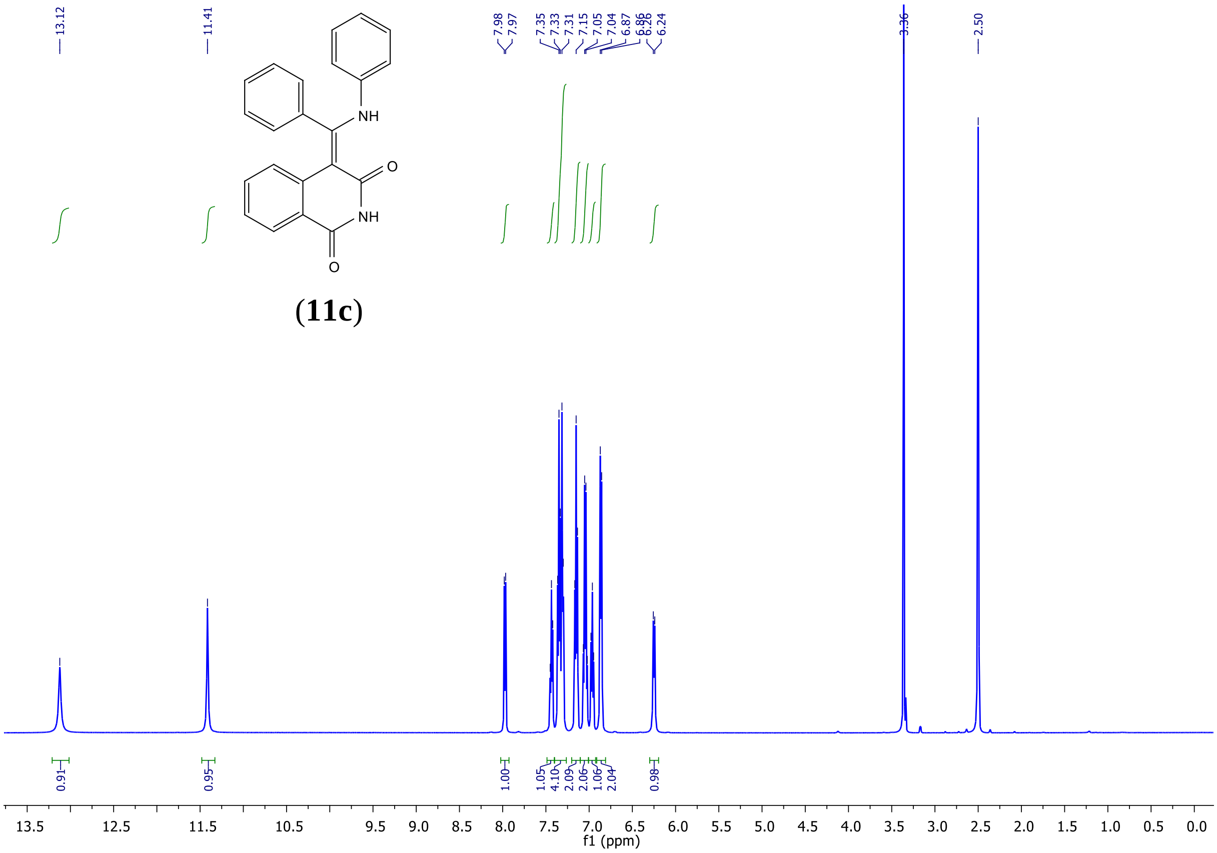
13.12
11.41
7.98
7.97
7.35
7.33
7.31
7.15
7.05
7.04
6.87
6.24
3.36
2.50
NH
O
NH
O
(11c)
0.91
0.95
1.00
1.05
4.10
2.09
2.06
1.06
2.04
0.98
f1 (ppm)

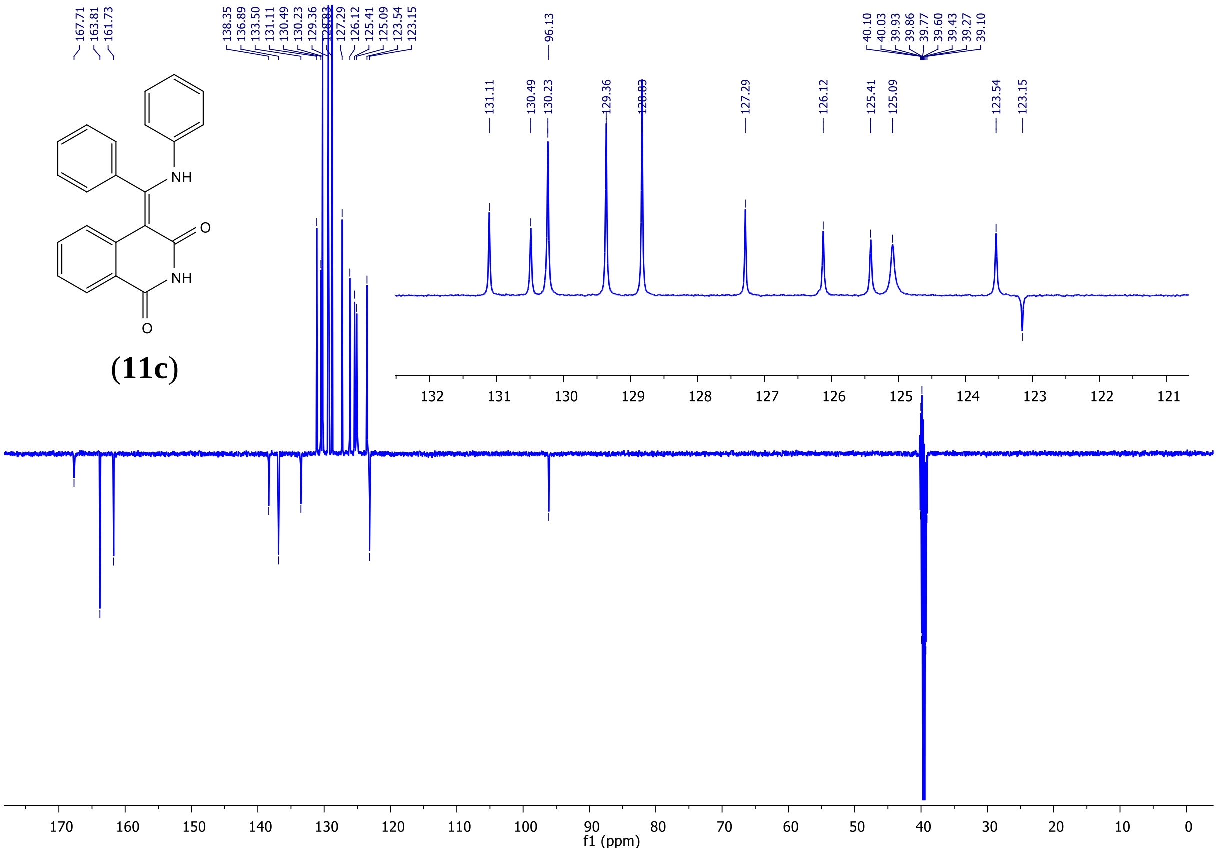

(**11c**)

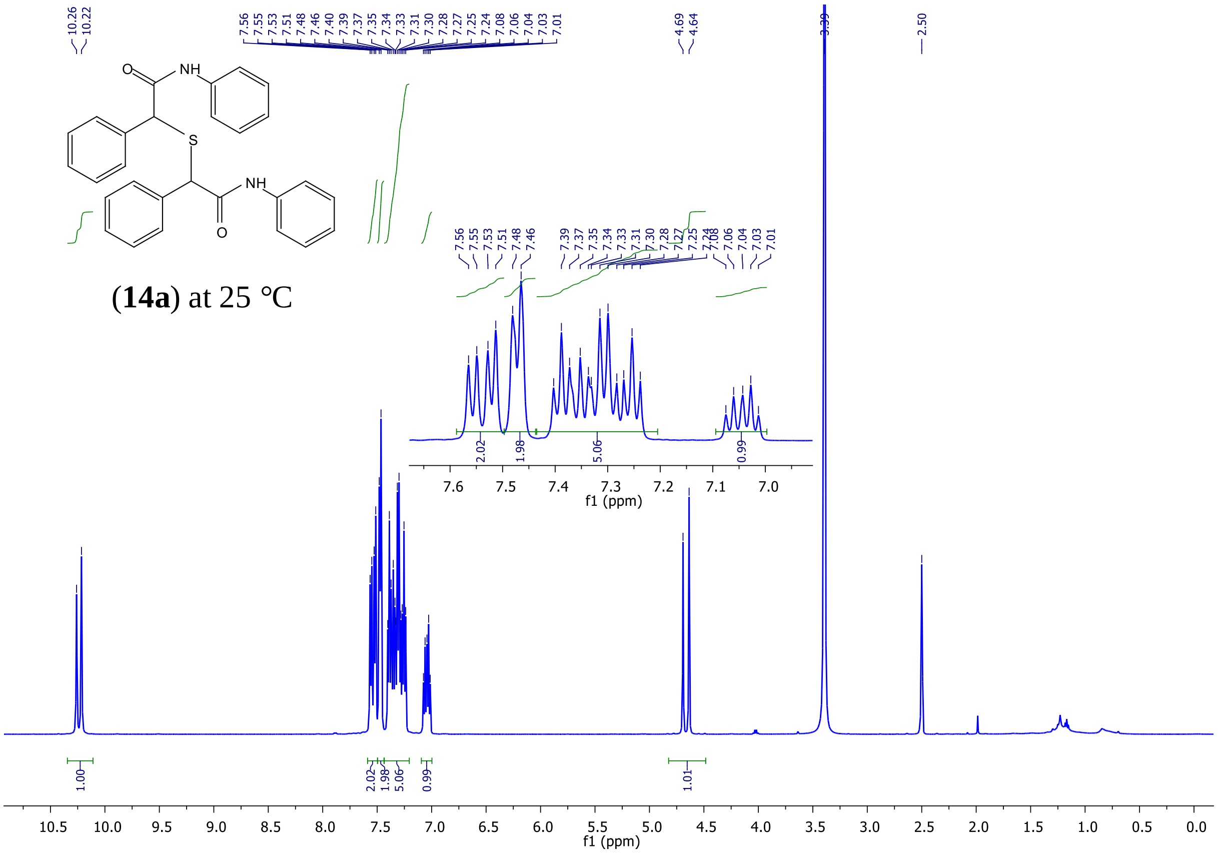

(**14a**) at 25 °C

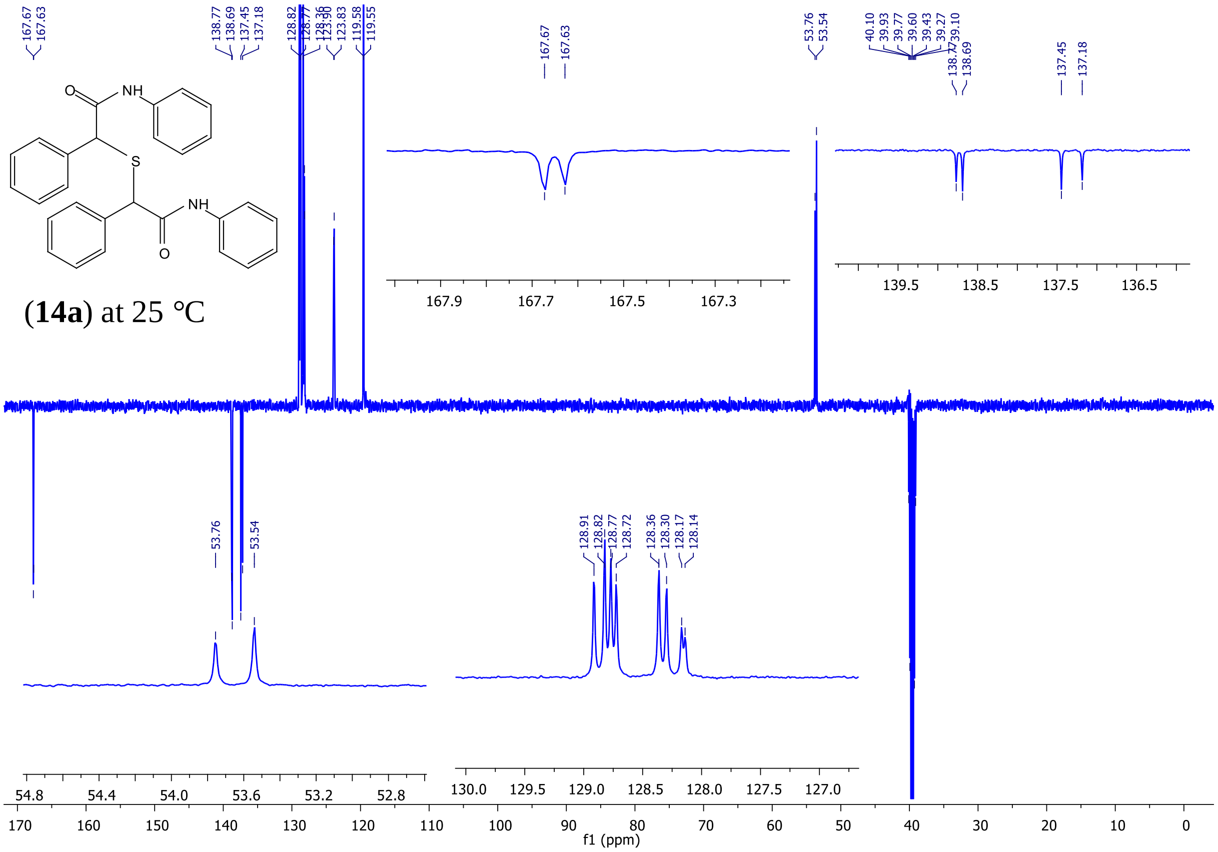
(14a) at 25 °C
f1 (ppm)

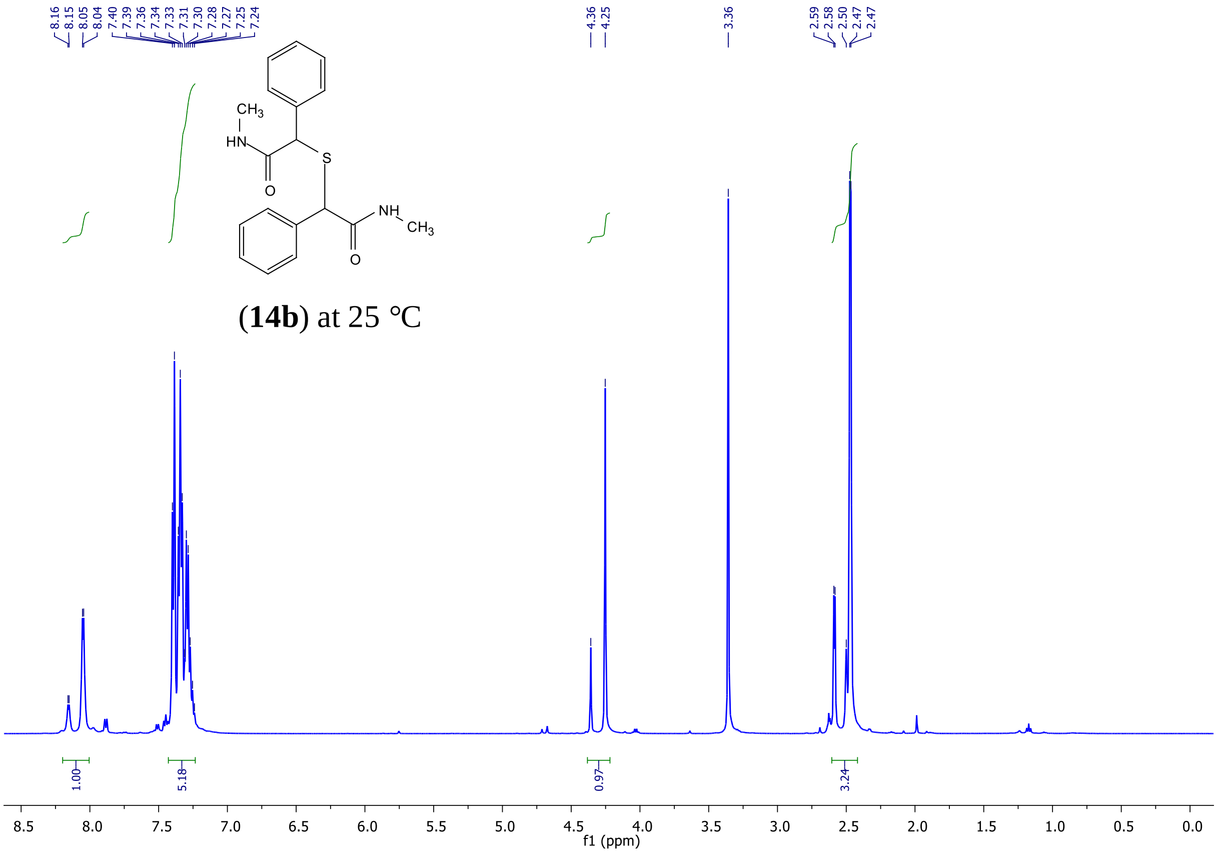
(14b) at 25 °C
f1 (ppm)

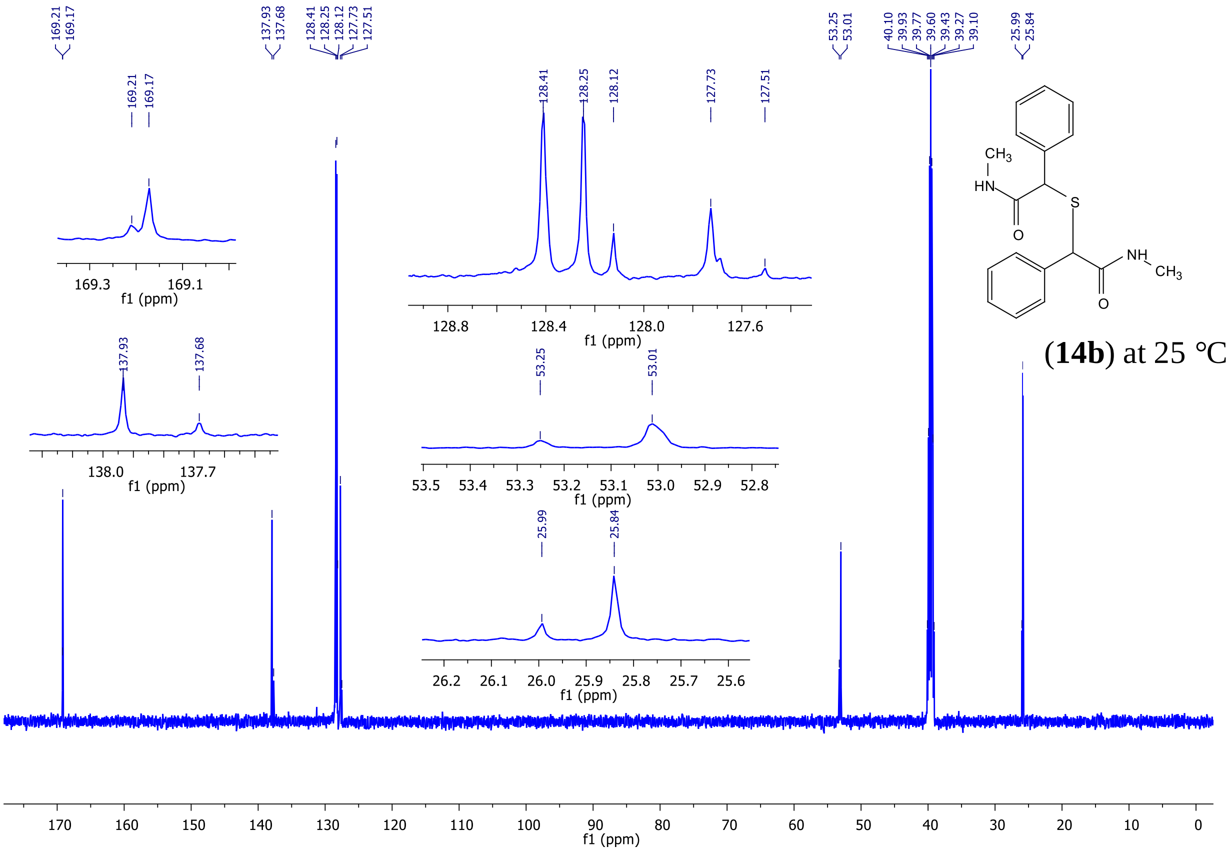
169.21
169.17
137.93
137.68
128.41
128.25
128.12
127.73
127.51
53.25
53.01
40.10
39.93
39.77
39.60
39.43
39.27
39.10
25.99
25.84
CH3
HN
S
O
NH
CH3
O
f1 (ppm)

(**14b**) at 25 ℃

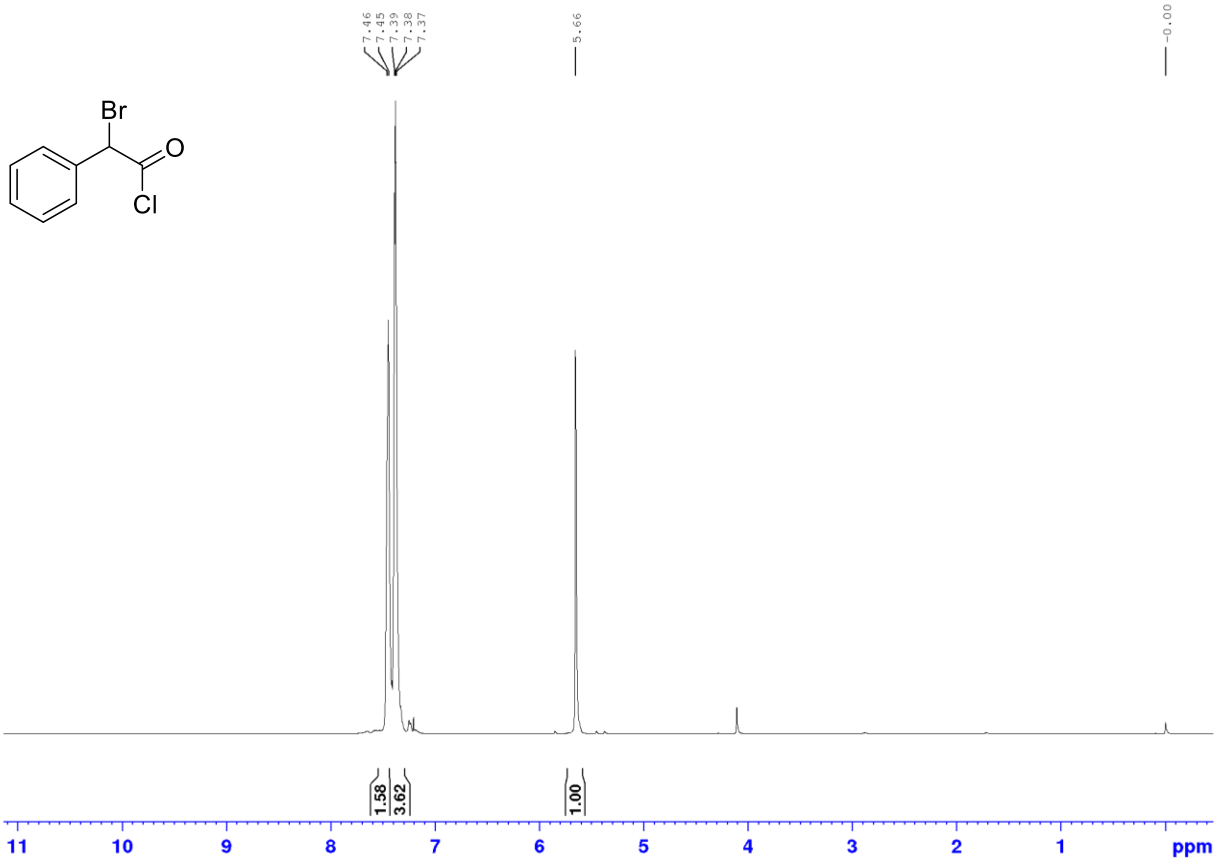
7.46
7.45
7.39
7.38
7.37
5.66
-0.00
Br
O
Cl
1.58
3.62
1.00
ppm

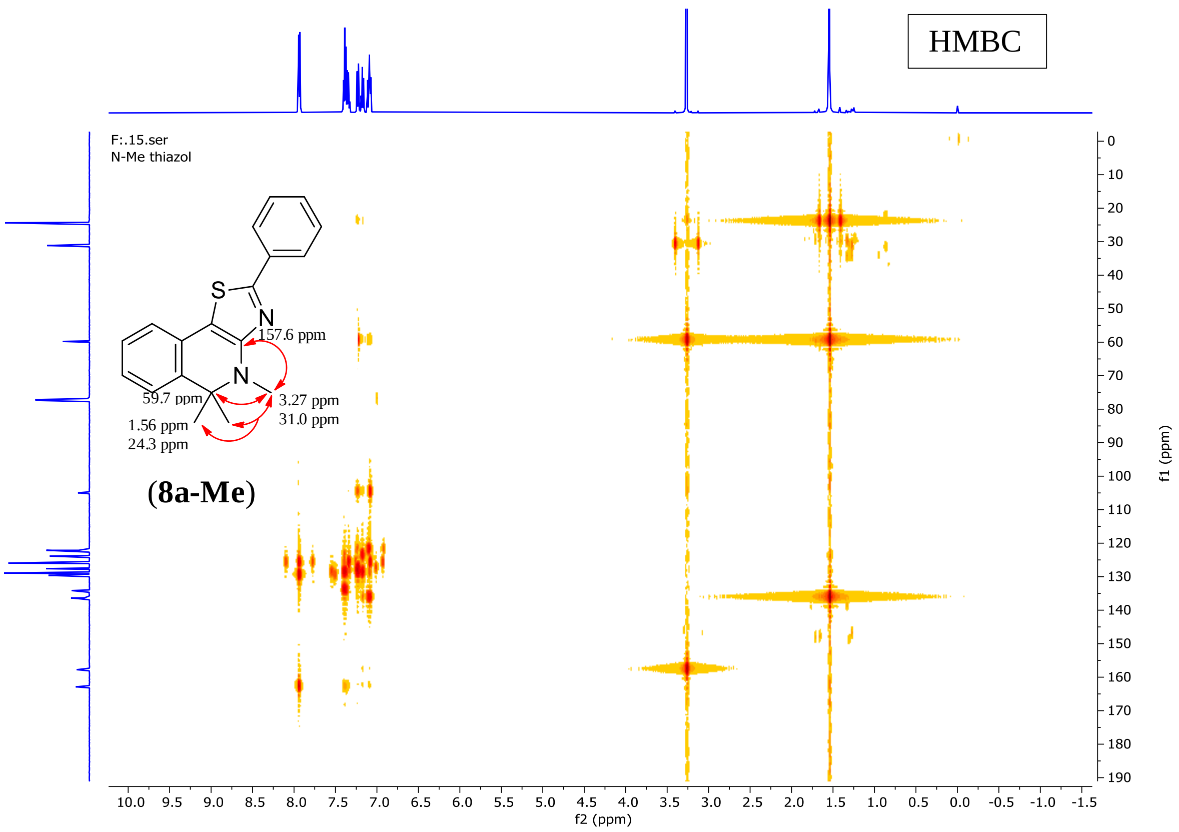
HMBC
F:.15.ser
N-Me thiazol
S
N
157.6 ppm
N
59.7 ppm
3.27 ppm
31.0 ppm
1.56 ppm
24.3 ppm
(8a-Me)
f2 (ppm)
f1 (ppm)

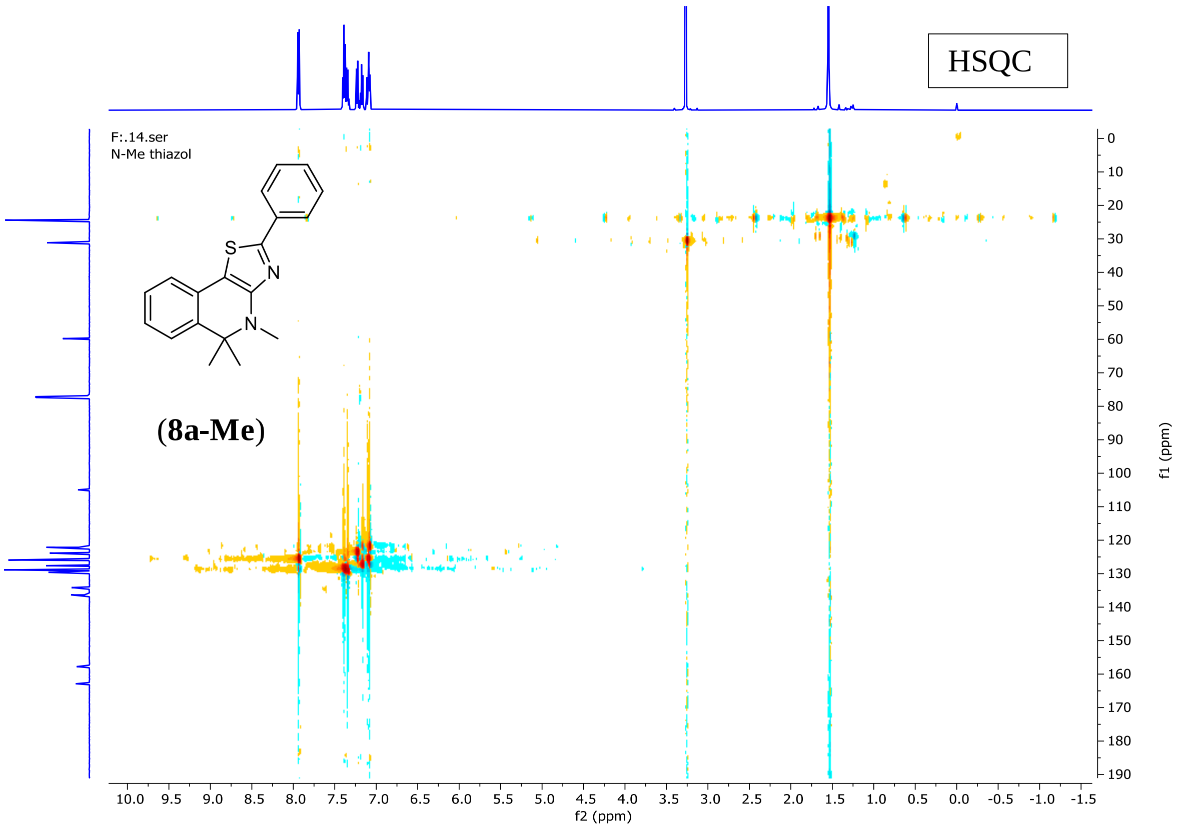
HSQC
F:.14.ser
N-Me thiazol
S
N
N
(8a-Me)
f2 (ppm)
f1 (ppm)

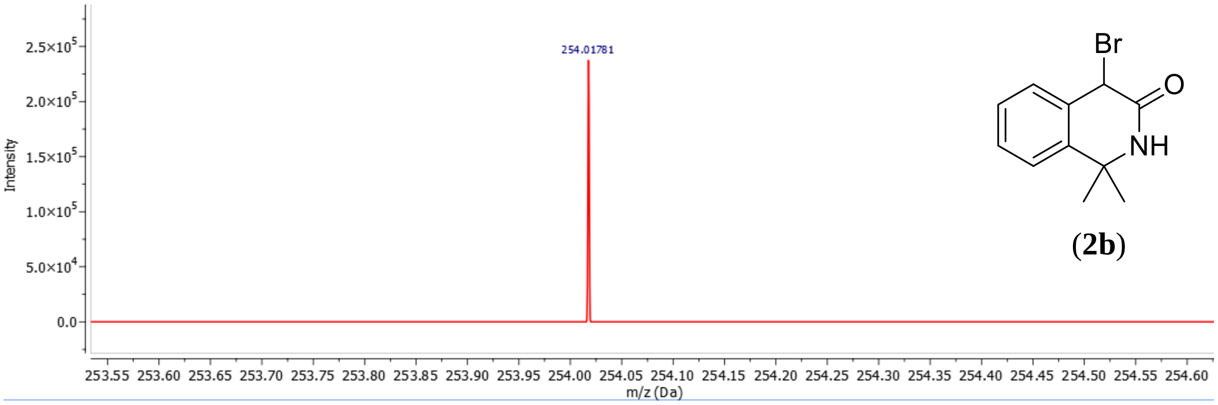
254.01781
Intensity
m/z (Da)
(2b)

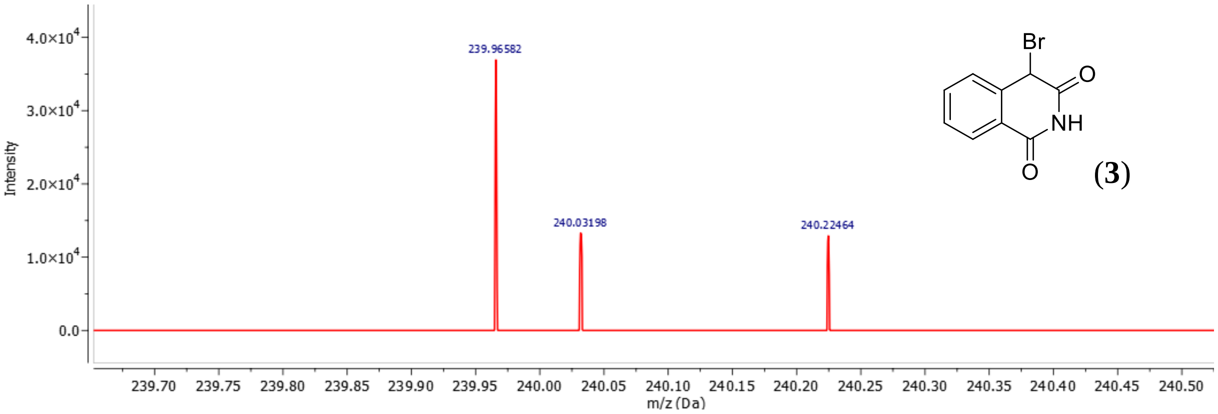
239.96582
240.03198
240.22464
Intensity
m/z (Da)
(3)

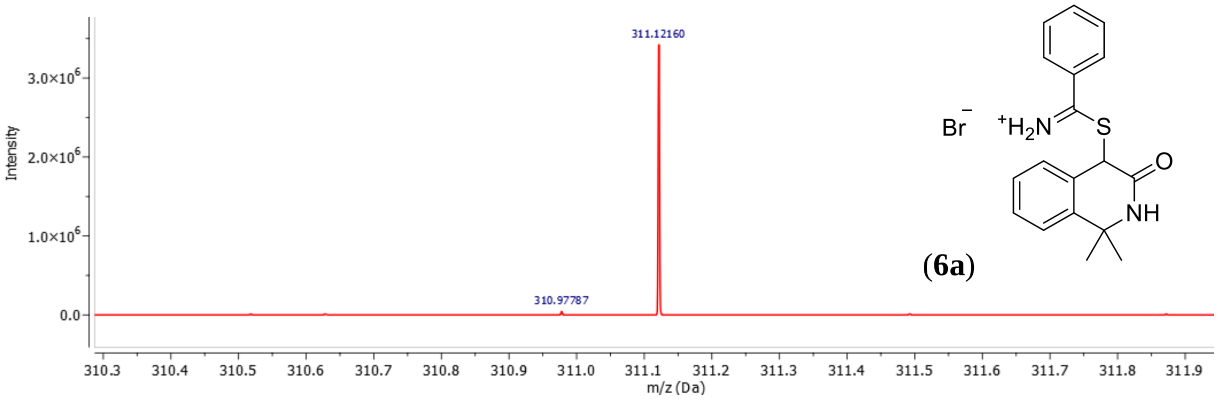
311.12160
310.97787
Intensity
m/z (Da)
(6a)

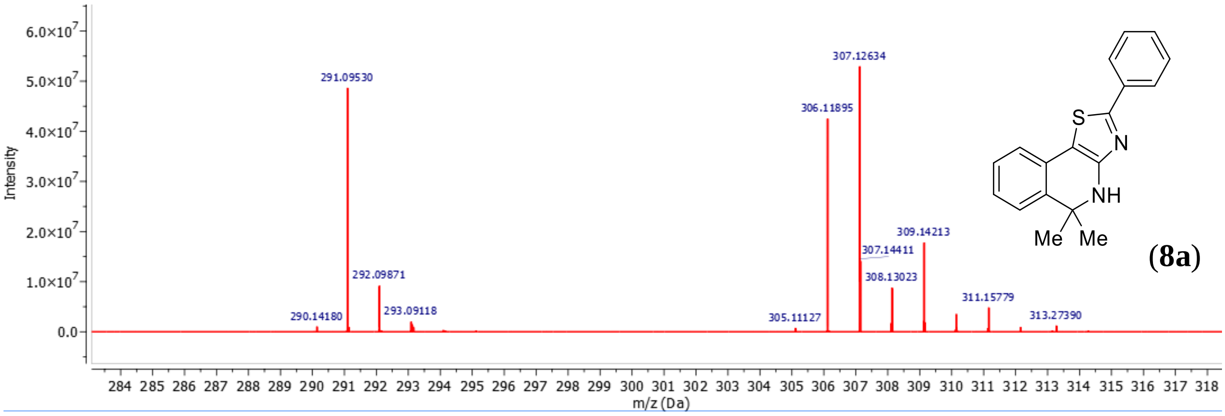
291.09530
292.09871
293.09118
290.14180
307.12634
306.11895
307.14411
308.13023
309.14213
311.15779
313.27390
305.11127
Intensity
m/z (Da)
(8a)

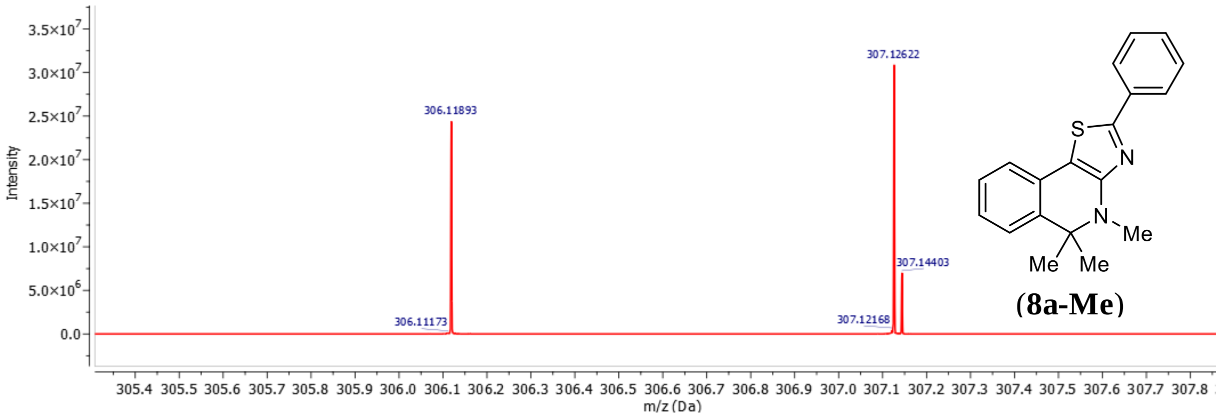
Intensity
m/z (Da)
306.11893
306.11173
307.12622
307.12168
307.14403
S
N
N
Me
Me
Me

(8a-Me)

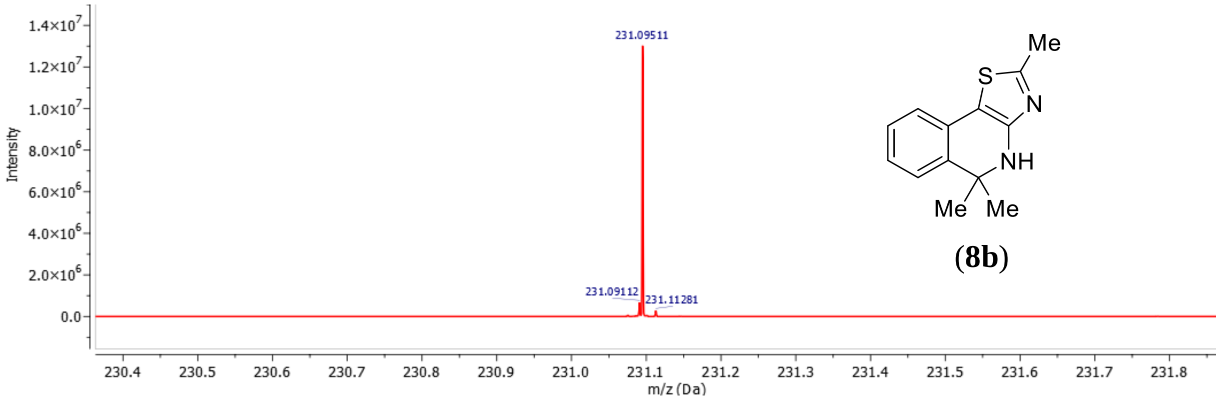
Intensity
m/z (Da)
231.09511
231.09112
231.11281
Me
S
N
NH
Me
Me

(8b)

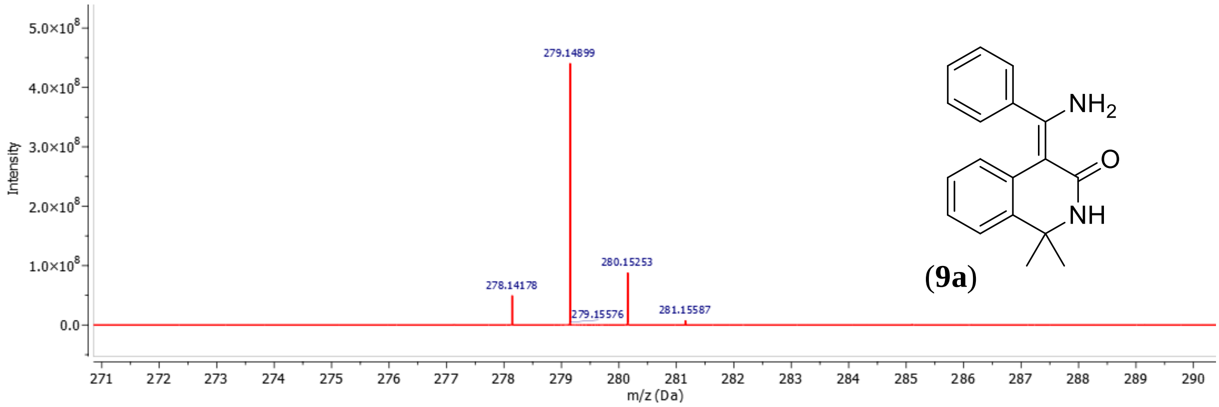
Intensity
m/z (Da)
279.14899
278.14178
279.15576
280.15253
281.15587
NH2
O
NH

(9a)

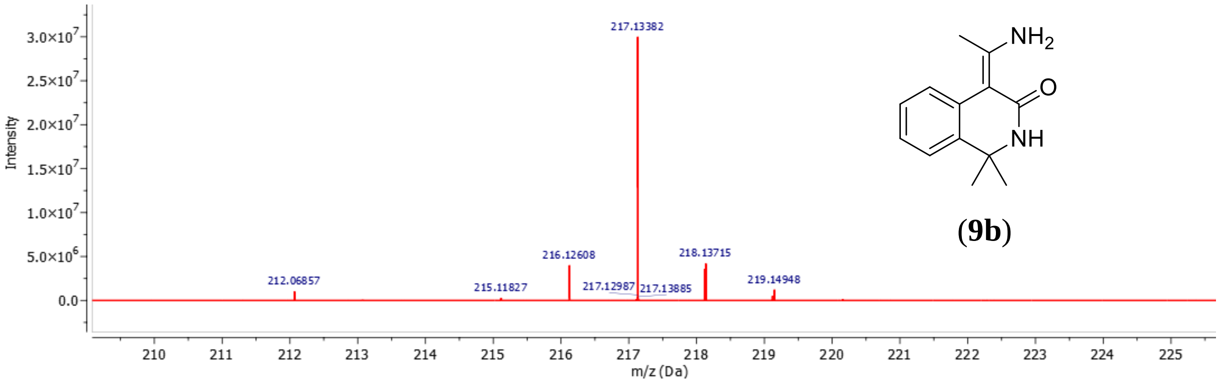
Intensity
m/z (Da)
217.13382
212.06857
215.11827
216.12608
217.12987
217.13885
218.13715
219.14948
NH2
O
NH

(9b)

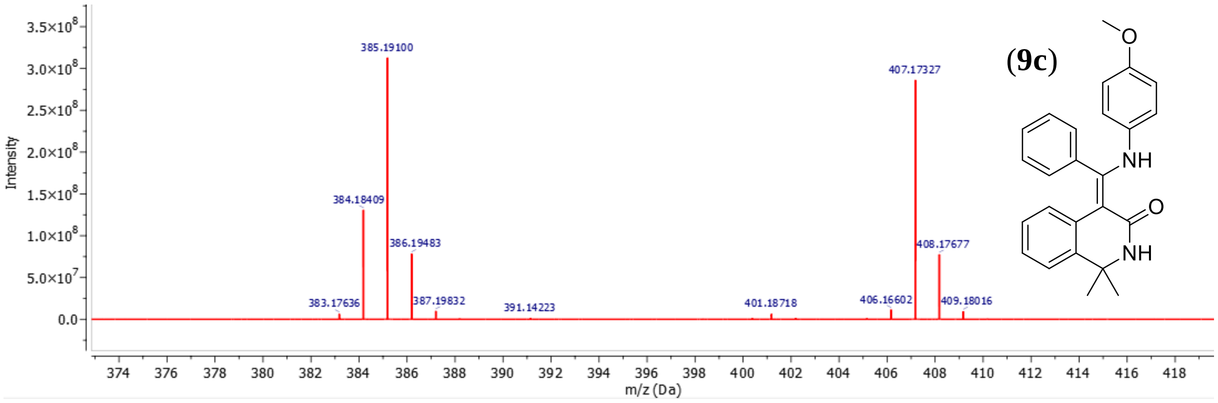
(9c)
Intensity
m/z (Da)
385.19100
407.17327
384.18409
386.19483
408.17677
383.17636
387.19832
391.14223
401.18718
406.16602
409.18016
O
NH
O
NH

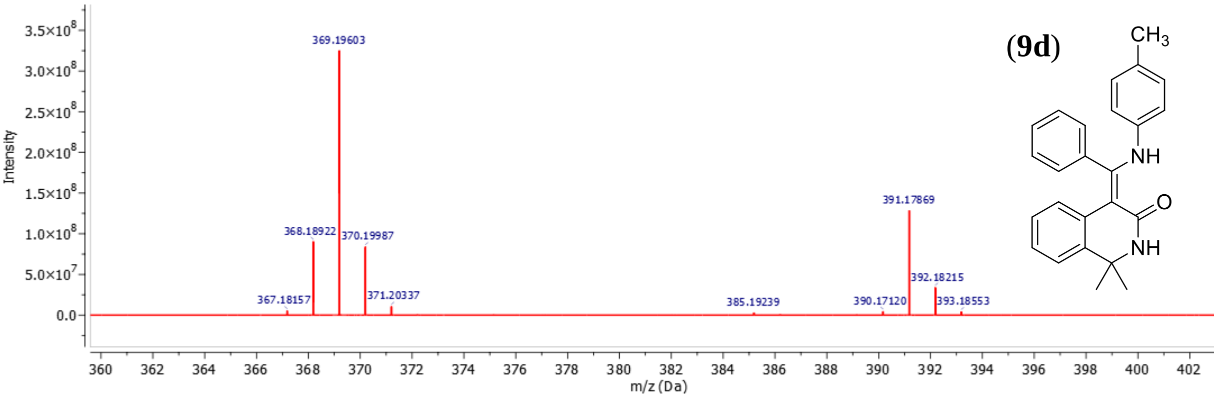
(9d)
Intensity
m/z (Da)
369.19603
391.17869
368.18922
370.19987
392.18215
367.18157
371.20337
385.19239
390.17120
393.18553
CH3
NH
O
NH

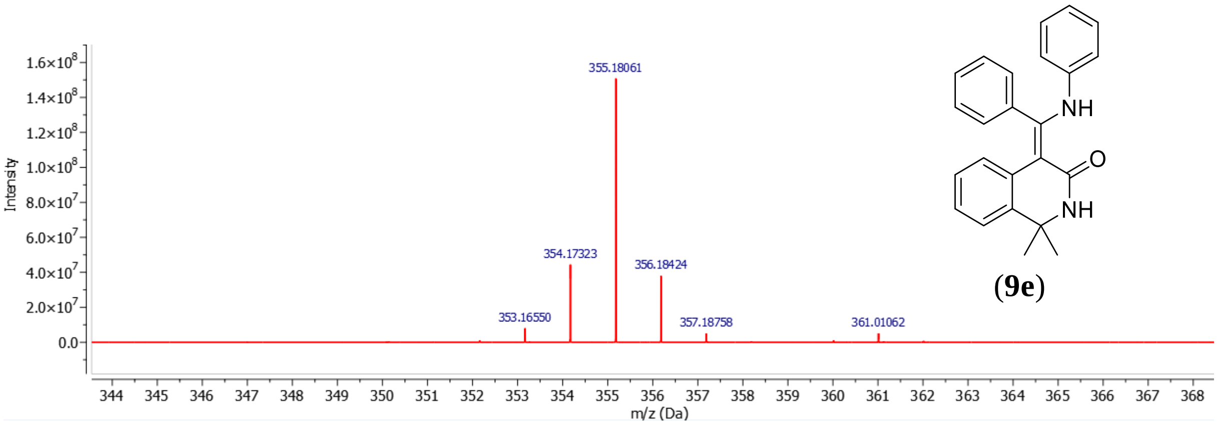
(9e)
Intensity
m/z (Da)
355.18061
354.17323
356.18424
353.16550
357.18758
361.01062
NH
O
NH

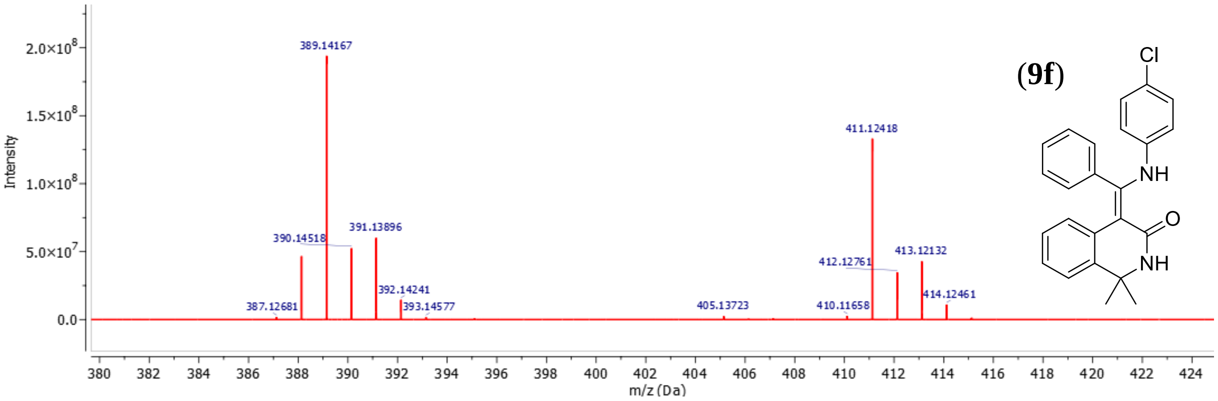
(9f)
Intensity
m/z (Da)
389.14167
411.12418
390.14518
391.13896
412.12761
413.12132
392.14241
393.14577
387.12681
405.13723
410.11658
414.12461
Cl
NH
O
NH

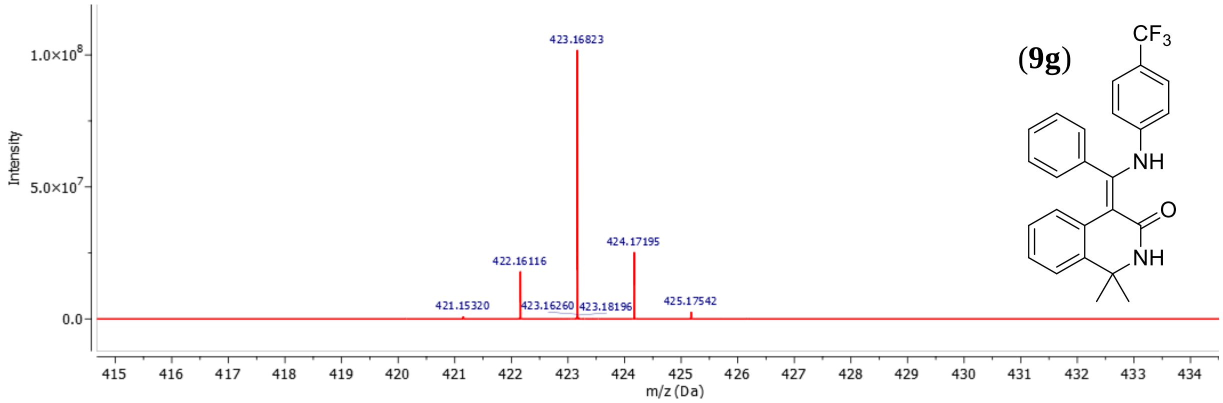
423.16823
422.16116
424.17195
421.15320
423.16260
423.18196
425.17542
Intensity
m/z (Da)
CF3
NH
O
NH

(9g)

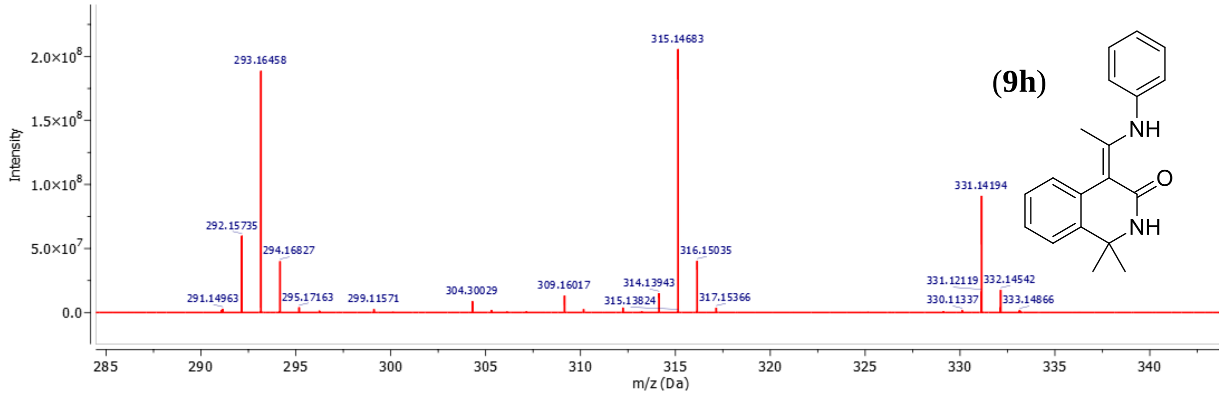
315.14683
293.16458
331.14194
292.15735
294.16827
316.15035
291.14963
295.17163
299.11571
304.30029
309.16017
314.13943
315.13824
317.15366
331.12119
330.11337
332.14542
333.14866
Intensity
m/z (Da)
NH
O
NH

(9h)

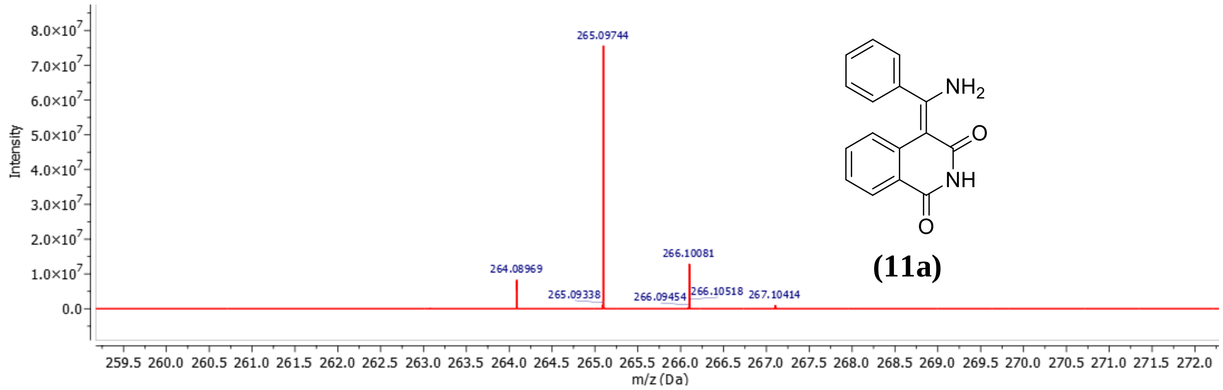
265.09744
264.08969
265.09338
266.10081
266.09454
266.10518
267.10414
Intensity
m/z (Da)
NH2
O
NH
O

(11a)

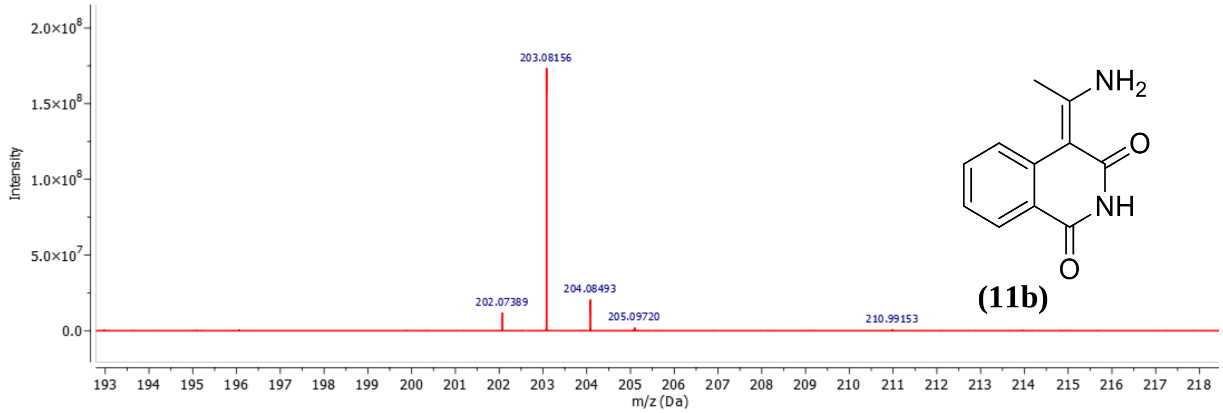
203.08156
202.07389
204.08493
205.09720
210.99153
Intensity
m/z (Da)
NH2
O
NH
O

(11b)

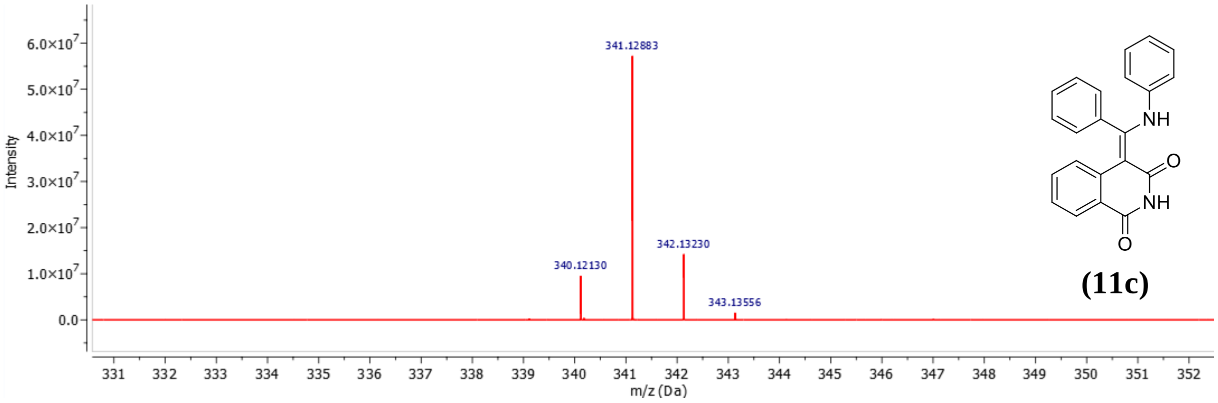

341.12883
340.12130
342.13230
343.13556
Intensity
m/z (Da)
NH
O
NH
O

(11c)

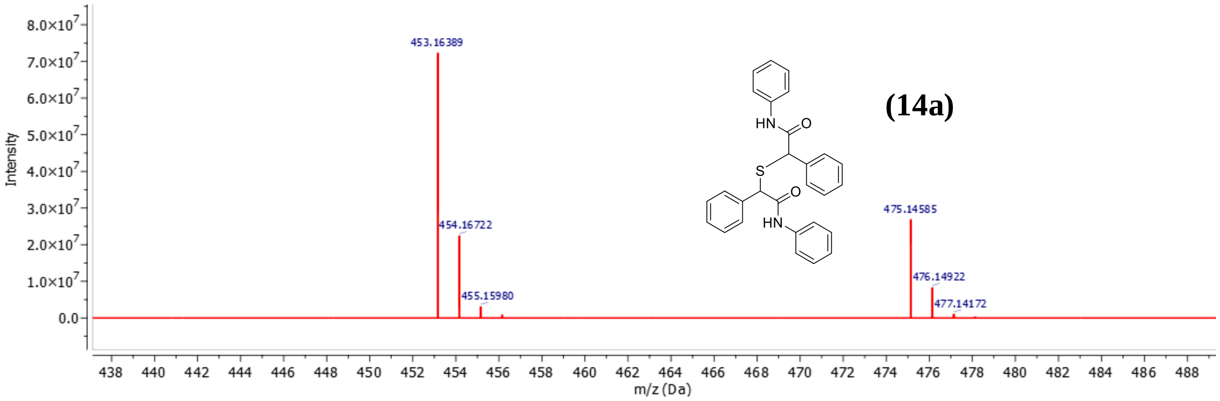

453.16389
454.16722
455.15980
475.14585
476.14922
477.14172
Intensity
m/z (Da)
HN
O
S
O
HN

(14a)

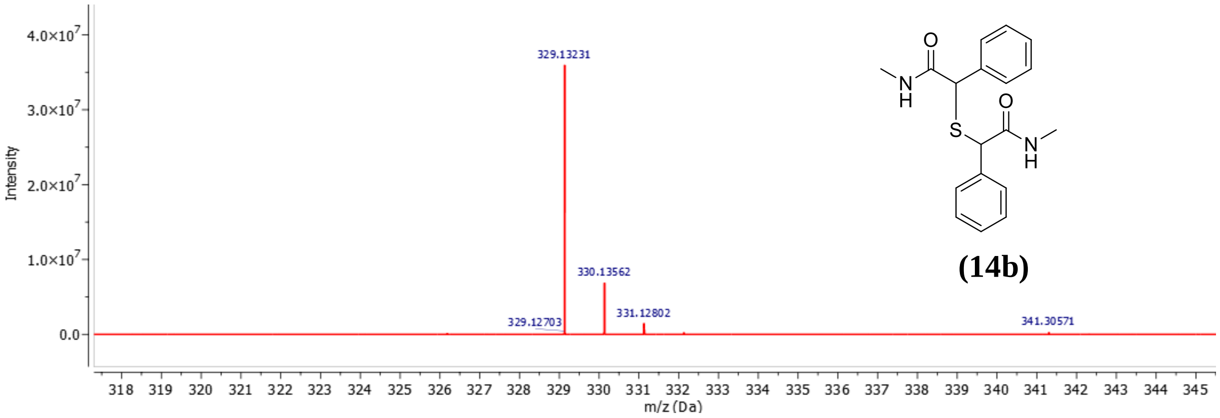

329.13231
329.12703
330.13562
331.12802
341.30571
Intensity
m/z (Da)
O
N
H
S
O
N
H

(14b)

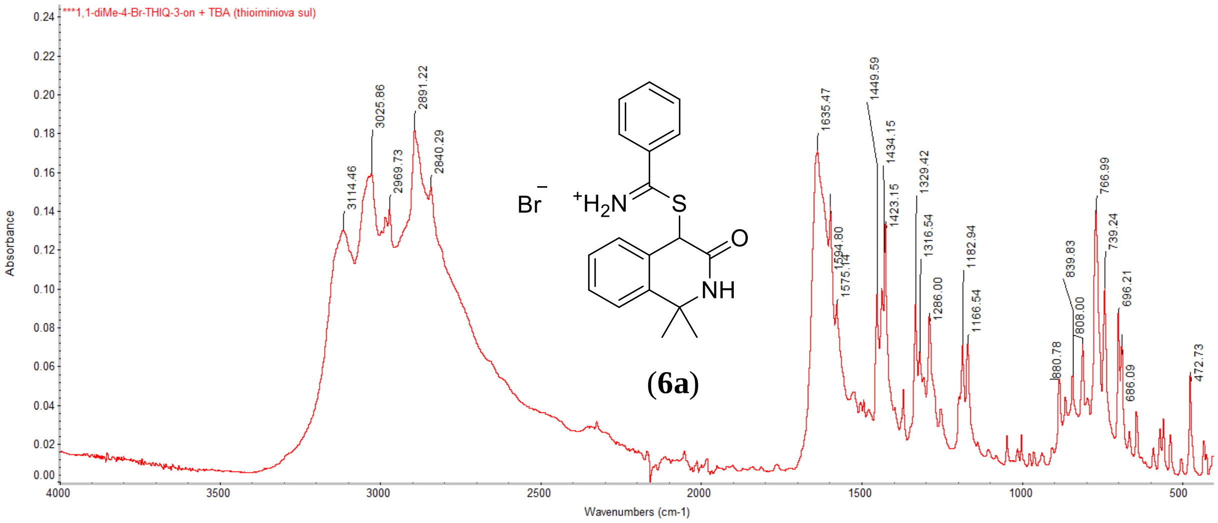
***1,1-diMe-4-Br-THIQ-3-on + TBA (thioiminiova sul)
Absorbance
Wavenumbers (cm-1)
3114.46
3025.86
2969.73
2891.22
2840.29
1635.47
1594.80
1575.14
1449.59
1434.15
1423.15
1329.42
1316.54
1286.00
1182.94
1166.54
880.78
839.83
808.00
766.99
739.24
696.21
686.09
472.73
Br
^+H_2N
S
O
NH
(6a)

Coordinates

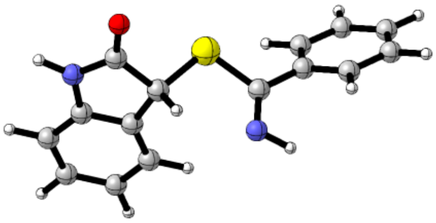

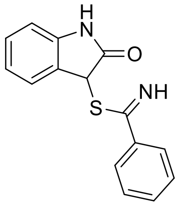

1-1 0 1 scf done: -1162.079869 Sum of electronic and thermal Free Energies -1161.887674
imag. freq.: 0

C	-2.8949580	-2.6569340	0.2264660
C	-1.8799140	-1.7277090	0.4884430
C	-2.1279900	-0.3864080	0.2493740
C	-3.3726010	0.0206730	-0.2512610
C	-4.3905220	-0.8833980	-0.5157300
C	-4.1307580	-2.2358810	-0.2675660
H	-2.7187510	-3.7104510	0.4088830
H	-0.9185980	-2.0506170	0.8693100
H	-5.3481780	-0.5542230	-0.9007750
H	-4.9066120	-2.9668250	-0.4648970
N	-3.3733740	1.4132450	-0.4149940
H	-4.1616090	1.9504110	-0.7535860
C	-2.1851000	1.9887150	-0.0504070
O	-1.9156710	3.1745070	-0.0868940
C	-1.2761490	0.8374260	0.4337080
H	-1.0051550	1.0087950	1.4783180
S	0.3049600	0.8505980	-0.4960730
C	1.3992250	0.1029650	0.7091180
C	2.7797270	-0.0955760	0.1887520
C	3.8694220	0.2040780	1.0174840
C	3.0104300	-0.5887340	-1.1014470
C	5.1691790	0.0118690	0.5606090
H	3.6966420	0.6032450	2.0103250
C	4.3137760	-0.7890890	-1.5501090
H	2.1762440	-0.8385380	-1.7455560
C	5.3942950	-0.4867230	-0.7231340
H	6.0063850	0.2564950	1.2040730
H	4.4830710	-1.1840760	-2.5450920
H	6.4078480	-0.6371760	-1.0767960
N	0.9882870	-0.1710370	1.8810850
H	1.7345910	-0.5888090	2.4394830

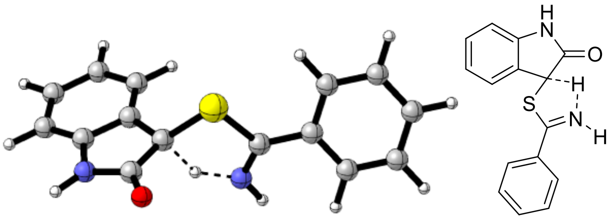

31

1-TS1 0 1 scf done: -1162.054171 Sum of electronic and thermal Free Energies -1161.865353 imag. freq.: 1 (-1593.5)

C	3.5154870	2.3991040	0.3947370
C	2.2824270	1.7785390	0.1553840
C	2.2445440	0.4016370	-0.0267140
C	3.4448490	-0.3423560	0.0318270
C	4.6709050	0.2593660	0.2781150
C	4.6919120	1.6481950	0.4587680
H	3.5561820	3.4737030	0.5327310
H	1.3709460	2.3645550	0.1073980
H	5.5820520	-0.3263510	0.3224710
H	5.6374130	2.1440070	0.6476360
N	3.1425320	-1.6841680	-0.1936750
H	3.8224380	-2.4309690	-0.2371760
C	1.7884800	-1.8818570	-0.4432600
O	1.2834360	-2.9632820	-0.7342360
C	1.1489770	-0.5618980	-0.2198410
H	0.4081040	-0.6893790	0.9960600
S	-0.3342950	-0.1491900	-1.1009040
C	-1.4052230	-0.2031950	0.3445750
C	-2.8321500	0.0935720	0.1953220
C	-3.7600160	-0.4708530	1.0842360
C	-3.2811130	0.9353580	-0.8321200
C	-5.1141780	-0.1931110	0.9448370
H	-3.4247150	-1.1409430	1.8668560
C	-4.6369440	1.2170910	-0.9596430
H	-2.5733370	1.3888770	-1.5152520
C	-5.5544550	0.6528280	-0.0743050
H	-5.8270300	-0.6391690	1.6281590
H	-4.9759960	1.8778890	-1.7484800
H	-6.6112730	0.8700620	-0.1784390
N	-0.7878120	-0.5383050	1.4127930
H	-1.2616810	-0.5777580	2.3100380

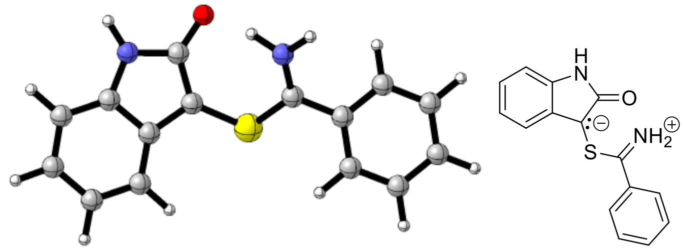

(15')

31

1-2 0 1 scf done: -1162.070177 Sum of electronic and thermal Free Energies -1161.876540 imag. freq.: 0

C	-3.9541310	-2.2703880	0.1861750
C	-2.6497770	-1.8283420	-0.0471420
C	-2.3989110	-0.4531950	-0.1243530
C	-3.4880760	0.4542240	0.0372670
C	-4.7843740	0.0161950	0.2708260
C	-5.0100540	-1.3630660	0.3449530
H	-4.1514170	-3.3350510	0.2484020
H	-1.8436460	-2.5447070	-0.1631760
H	-5.5960420	0.7250460	0.3927820
H	-6.0136200	-1.7300700	0.5275700
N	-2.9851410	1.7400980	-0.0803360
H	-3.5138530	2.5917480	0.0436900
C	-1.6112840	1.7255530	-0.3193850
O	-0.9139230	2.7742850	-0.4352860
C	-1.2290790	0.3565820	-0.3392780
H	0.4107760	2.2321290	0.3883020
S	0.3189980	-0.3112820	-0.8075120
C	1.5289170	0.5440540	0.0831490
C	2.8921510	-0.0048360	0.0700680
C	3.9914500	0.8682610	0.0317550
C	3.1079890	-1.3909370	0.1076600
C	5.2839080	0.3586140	0.0337310
H	3.8350460	1.9387800	-0.0286350
C	4.4043430	-1.8921470	0.1124870
H	2.2668860	-2.0705320	0.1624280
C	5.4925840	-1.0204720	0.0748860
H	6.1283710	1.0363000	-0.0061960
H	4.5652920	-2.9627250	0.1535130
H	6.5021460	-1.4147470	0.0771990
N	1.2543280	1.6782180	0.6801780
H	1.9863410	2.1403070	1.2087770

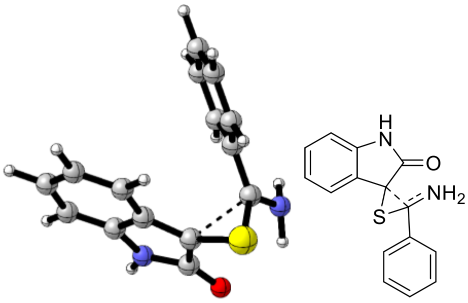

31
1-TS2 0 1 scf done:-1162.055802 Sum of electronic and thermal Free Energies -1161.861967
imag. freq.: 1 (-336.7)

C	-0.9981720	2.9218090	0.9530800
C	-0.6407100	1.6103180	1.2805300
C	-1.1898350	0.5606210	0.5480940
C	-2.0967540	0.8395470	-0.5069870
C	-2.4586410	2.1377540	-0.8344820
C	-1.8932000	3.1806300	-0.0901240
H	-0.5766000	3.7476850	1.5146610
H	0.0516770	1.4111280	2.0893400
H	-3.1532800	2.3370480	-1.6424510
H	-2.1577210	4.2045680	-0.3287660
N	-2.4938660	-0.3671200	-1.0769590
H	-3.1497490	-0.4631660	-1.8397700
C	-1.9063060	-1.4535700	-0.4369640
O	-2.0732070	-2.6376840	-0.7481260
C	-1.0266240	-0.8743740	0.5771240
H	-0.0346120	-3.3631070	-0.6265020
S	-0.0684660	-1.8458310	1.7100880
C	0.7800640	-1.6925280	0.1683620
C	1.6887450	-0.5596370	-0.1064850
C	1.8124600	-0.0724040	-1.4168730
C	2.4098850	0.0556420	0.9217700
C	2.6497520	1.0034400	-1.6883510
H	1.2317100	-0.5127410	-2.2195400
C	3.2545240	1.1276240	0.6437210
H	2.3137590	-0.3077320	1.9368710
C	3.3765630	1.6048280	-0.6593210
H	2.7273930	1.3796710	-2.7018560
H	3.8135940	1.5908840	1.4484340
H	4.0279460	2.4444740	-0.8728190
N	0.7796060	-2.7557870	-0.6721840
H	1.1195790	-2.5959280	-1.6137960

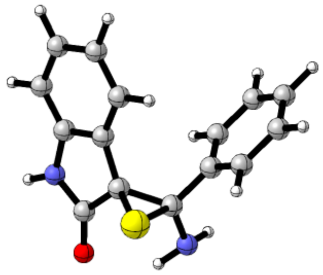

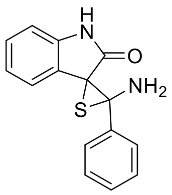

(**18**)

31

1-3 0 1 scf done: -1162.073785 Sum of electronic and thermal Free Energies -1161.879172

imag. freq.: 0

C	-1.1178380	2.8925580	0.8488430
C	-0.4981490	1.6407880	0.9292570
C	-1.1262440	0.5475930	0.3523810
C	-2.3700620	0.7054630	-0.2774510
C	-2.9997260	1.9376220	-0.3659860
C	-2.3493240	3.0359890	0.2061630
H	-0.6376260	3.7571820	1.2913590
H	0.4524230	1.5293400	1.4350880
H	-3.9596050	2.0426840	-0.8572040
H	-2.8158650	4.0129530	0.1522740
N	-2.8120060	-0.5379500	-0.7515420
H	-3.6914250	-0.6917170	-1.2283660
C	-1.9349280	-1.5466150	-0.4656030
O	-2.0668230	-2.7242130	-0.7601880
C	-0.7543580	-0.8885750	0.2478860
H	-0.0086910	-3.0763580	-1.0094020
S	0.0110550	-1.7680630	1.6823520
C	0.6425190	-1.4094130	-0.0364310
C	1.7395560	-0.3916530	-0.1838000
C	1.7136640	0.4493710	-1.3007580
C	2.7860250	-0.2888330	0.7304830
C	2.7205080	1.3916910	-1.4928370
H	0.8964980	0.3759980	-2.0097760
C	3.7952810	0.6536540	0.5375650
H	2.8052640	-0.9357800	1.5992100
C	3.7628500	1.4973960	-0.5716750
H	2.6869130	2.0469230	-2.3558190
H	4.6036850	0.7297330	1.2558650
H	4.5442280	2.2346840	-0.7172470
N	0.8488220	-2.5497680	-0.8624280
H	1.2537840	-2.2839050	-1.7547140

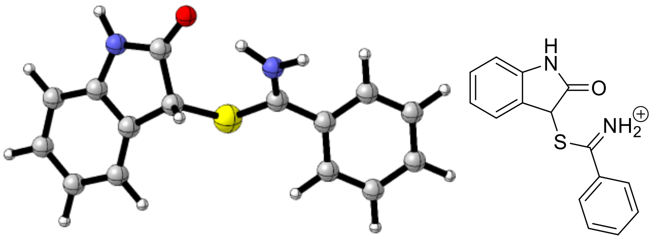 **(15)**

32

1-4 1 1 scf done: -1162.540262 Sum of electronic and thermal Free Energies -1162.333094

imag. freq.: 0

C	-2.2483930	-2.7206070	-0.1247690
C	-1.5790650	-1.4920670	-0.0762660
C	-2.3302960	-0.3389890	0.0791920
C	-3.7257090	-0.4093740	0.1824840
C	-4.4050160	-1.6148620	0.1399480
C	-3.6388800	-2.7752940	-0.0187440
H	-1.6812200	-3.6355170	-0.2452250
H	-0.4993890	-1.4458550	-0.1585830
H	-5.4836870	-1.6565980	0.2265880
H	-4.1404860	-3.7352140	-0.0578440
N	-4.2468560	0.8880980	0.3397020
H	-5.2314590	1.1019460	0.4494780
C	-3.2792700	1.8338840	0.3537330
O	-3.4275430	3.0429550	0.4947220
C	-1.9322900	1.1079450	0.1696290
H	-1.2558110	1.3388580	0.9948890
S	-1.0693780	1.5908780	-1.3986390
C	-0.4044360	3.1658250	-1.0655990
C	0.6931130	3.5986860	-1.9304090
C	0.7607360	4.9461200	-2.3261500
C	1.6607720	2.6857550	-2.3808590
C	1.7869840	5.3691490	-3.1586460
H	-0.0044660	5.6450950	-2.0102070
C	2.6885540	3.1229180	-3.2064980
H	1.6290150	1.6528420	-2.0591850
C	2.7515350	4.4602290	-3.5974120
H	1.8305070	6.4045310	-3.4738710
H	3.4433650	2.4214370	-3.5400820
H	3.5521040	4.7953160	-4.2465230
N	-0.8834690	3.9404280	-0.1336460
H	-0.4135690	4.8136920	0.0893410
H	-1.7905420	3.7421680	0.3173180

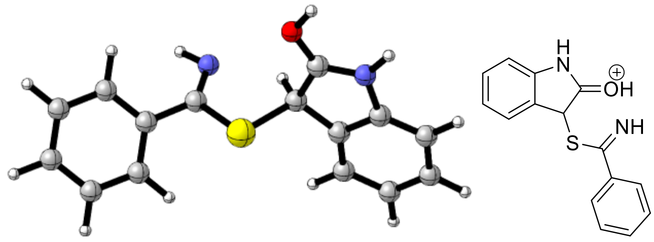

(15'')

32

1-5 1 1 scf done: -1162.496961 Sum of electronic and thermal Free Energies -1162.292359
imag. freq.: 0

C	3.8231940	-2.1553680	-0.6831850
C	2.5465680	-1.5854010	-0.7411700
C	2.3790370	-0.3114300	-0.2261790
C	3.4687260	0.3600080	0.3315380
C	4.7400930	-0.1754910	0.3996810
C	4.8993740	-1.4627690	-0.1232620
H	3.9785950	-3.1519750	-1.0783360
H	1.7134000	-2.1265380	-1.1729150
H	5.5662410	0.3719590	0.8352060
H	5.8768800	-1.9283470	-0.0901370
N	3.0215300	1.6371540	0.7841340
H	3.6268370	2.3245350	1.2314280
C	1.7447400	1.7972740	0.5438860
O	1.0175670	2.8338630	0.8270670
C	1.1803470	0.5950430	-0.1540080
H	0.8114090	0.9190290	-1.1418030
S	-0.3041660	-0.0929250	0.6655490
C	-1.5643850	0.4225770	-0.5230600
C	-2.9135800	-0.0975740	-0.1899780
C	-4.0264800	0.7327240	-0.3816620
C	-3.0967860	-1.3995740	0.2929210
C	-5.3038720	0.2633690	-0.0965400
H	-3.8871900	1.7471170	-0.7366710
C	-4.3793530	-1.8667880	0.5676400
H	-2.2465970	-2.0569500	0.4282590
C	-5.4830360	-1.0372810	0.3763280
H	-6.1590280	0.9137170	-0.2388280
H	-4.5150460	-2.8800490	0.9270660
H	-6.4800510	-1.4015530	0.5957340
N	-1.2453110	1.1733130	-1.4927870
H	-2.0379520	1.3592650	-2.1089550
H	1.5214280	3.5431630	1.2723530

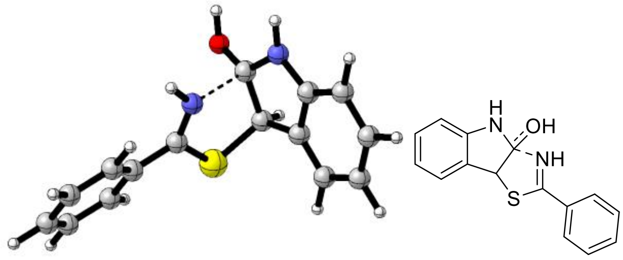

32

1-TS4 1 1 scf done: -1162.496053 Sum of electronic and thermal Free Energies -1162.289356
imag. freq.: 1 (-264.1)

C	3.3989110	-2.0628810	0.8898970
C	2.5898390	-1.7533530	-0.2093110
C	2.3616360	-0.4200910	-0.5011690
C	2.9334720	0.5816930	0.2899180
C	3.7374620	0.2990660	1.3807770
C	3.9629720	-1.0506590	1.6691630
H	3.5886770	-3.1000620	1.1385730
H	2.1488290	-2.5386860	-0.8117450
H	4.1699430	1.0887070	1.9826900
H	4.5852140	-1.3116420	2.5170690
N	2.5559370	1.8428310	-0.2234410
H	2.7552720	2.7216960	0.2432570
C	1.5939840	1.6946980	-1.1498370
O	1.2216800	2.6560240	-1.9704820
C	1.5933580	0.2437650	-1.6209020
S	-0.0767340	-0.4078440	-1.9727570
C	-0.8782490	0.5272490	-0.6877540
C	-2.2947970	0.2375380	-0.4040470
C	-2.8119940	0.5082610	0.8712110
C	-3.1307350	-0.3020700	-1.3909010
C	-4.1477150	0.2447360	1.1492850
H	-2.1684200	0.9022960	1.6489120
C	-4.4688380	-0.5544760	-1.1086060
H	-2.7521370	-0.5030370	-2.3863780
C	-4.9785050	-0.2837620	0.1605510
H	-4.5393320	0.4462510	2.1392300
H	-5.1120920	-0.9610870	-1.8796570
H	-6.0201850	-0.4869190	0.3802820
N	-0.1335980	1.3811450	-0.1051650
H	-0.5485610	1.9892290	0.5993670
H	2.1412220	0.1903810	-2.5668950
H	1.4398140	3.5367210	-1.6175320

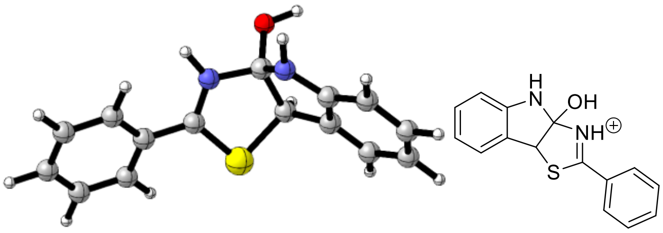

(**16·H**$^{+}$)

32

1-6 1 1 scf done: -1162.516500 Sum of electronic and thermal Free Energies -1162.308157
imag. freq.: 0

C	4.5941480	-1.2428760	-0.3802730
C	3.5497190	-0.8358810	-1.2160970
C	2.4604490	-0.1922920	-0.6499720
C	2.4032260	0.0532810	0.7287340
C	3.4374800	-0.3411460	1.5699170
C	4.5300450	-0.9948520	0.9935520
H	5.4560280	-1.7477300	-0.7992530
H	3.5892970	-1.0174760	-2.2842000
H	3.3976510	-0.1451660	2.6345550
H	5.3479160	-1.3111230	1.6311540
N	1.2109600	0.7035530	1.0648090
H	1.1491640	1.2648710	1.9059530
C	0.5562630	1.1837220	-0.1247410
O	0.6148580	2.5701080	-0.2949630
C	1.2205210	0.3562990	-1.2772670
S	-0.0346760	-0.9530560	-1.7274990
C	-1.2822310	-0.1721220	-0.8195530
C	-2.6492810	-0.6639600	-0.7992940
C	-3.4709310	-0.4230100	0.3160930
C	-3.1458090	-1.3962170	-1.8905840
C	-4.7707550	-0.9081220	0.3297890
H	-3.0888790	0.1087450	1.1789780
C	-4.4501990	-1.8690780	-1.8692380
H	-2.5269290	-1.5700710	-2.7623640
C	-5.2622800	-1.6273070	-0.7608940
H	-5.3992260	-0.7324850	1.1941680
H	-4.8341210	-2.4232610	-2.7168130
H	-6.2788570	-2.0024470	-0.7452010
N	-0.8752680	0.8663530	-0.1349040
H	-1.5129670	1.4325670	0.4192150
H	1.3765190	0.9607530	-2.1680360
H	1.5115440	2.8690100	-0.0795840

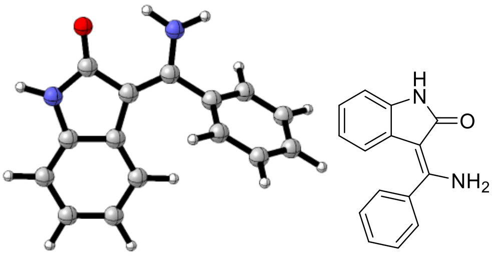

(**19**)

30

1-7 0 1 scf done: -763.880154 Sum of electronic and thermal Free Energies -763.685142
imag. freq.: 0

C	-1.3112920	3.1095820	0.4666960
C	-0.5364870	1.9724380	0.2177980
C	-1.1650310	0.7718470	-0.1215860
C	-2.5833740	0.7485180	-0.1759740
C	-3.3612420	1.8714730	0.0657820
C	-2.7058450	3.0644400	0.3862130
H	-0.8188370	4.0398020	0.7270070
H	0.5402700	2.0332470	0.2936870
H	-4.4427610	1.8192890	0.0136650
H	-3.2882910	3.9581670	0.5792160
N	-2.9954310	-0.5468550	-0.4786220
H	-3.9570640	-0.8511120	-0.5449390
C	-1.9176910	-1.4040040	-0.6025710
O	-2.0081690	-2.6204210	-0.8322740
C	-0.7142900	-0.5863000	-0.4145600
H	-0.0762640	-3.0502390	-0.7396860
C	0.5557020	-1.1307140	-0.5009340
C	1.7908830	-0.3088850	-0.4413940
C	2.0094840	0.6988240	-1.3861890
C	2.7573190	-0.5625240	0.5376640
C	3.1811190	1.4490020	-1.3476540
H	1.2631240	0.8898190	-2.1478980
C	3.9220180	0.2010640	0.5825600
H	2.5889450	-1.3429800	1.2707820
C	4.1360080	1.2062700	-0.3596420
H	3.3475100	2.2241290	-2.0868590
H	4.6605230	0.0100470	1.3527420
H	5.0444180	1.7969770	-0.3260400
N	0.7409000	-2.4541700	-0.6394570
H	1.6622440	-2.8219760	-0.8235920

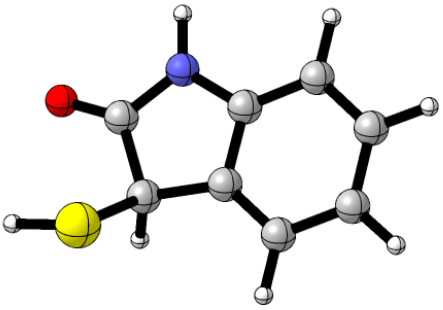
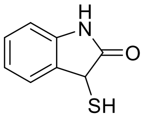

18

1-8 0 1 scf done: -837.455305 Sum of electronic and thermal Free Energies -837.357356
imag. freq.: 0

C	2.6750950	-1.6417410	-0.2290630
C	1.3465860	-2.0683900	-0.3537260
C	0.3438070	-1.1149140	-0.4091530
C	0.6627890	0.2492300	-0.3433660
C	1.9717630	0.6909910	-0.2219860
C	2.9770600	-0.2807650	-0.1634900
H	3.4727980	-2.3736230	-0.1847010
H	1.1091740	-3.1249410	-0.4051750
H	2.2060200	1.7476800	-0.1757110
H	4.0097750	0.0345980	-0.0675330
N	-0.5135270	1.0055390	-0.4358460
H	-0.5470370	2.0172160	-0.4205880
C	-1.6287750	0.2229320	-0.5788940
O	-2.7752930	0.6111530	-0.7081020
C	-1.1475460	-1.2410910	-0.5449480
H	-1.4575920	-1.7542270	-1.4558050
S	-1.8713540	-2.1303250	0.9062430
H	-3.1612690	-1.9567310	0.5418320

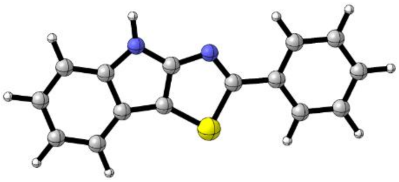 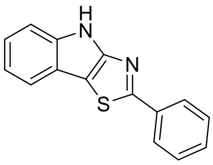

28

1-9 0 1 scf done: -1085.609928 Sum of electronic and thermal Free Energies -1085.440856

imag. freq.: 0

C	-4.0718390	-2.3093150	0.0761240
C	-2.6936620	-2.4598160	0.0008370
C	-1.8812580	-1.3218730	0.1084360
C	-2.4921230	-0.0366000	0.2930610
C	-3.8774260	0.1060730	0.3677340
C	-4.6560980	-1.0409530	0.2574180
H	-4.7108880	-3.1810500	-0.0057290
H	-2.2481340	-3.4380500	-0.1387490
H	-4.3279110	1.0819350	0.5073700
H	-5.7353060	-0.9555150	0.3123480
N	-1.5016960	0.9299530	0.3724990
H	-1.6520660	1.9209120	0.4976690
C	-0.2850850	0.3032220	0.2438150
C	-0.4766540	-1.0648490	0.0823050
S	1.0758380	-1.8187670	-0.0766970
C	1.8337670	-0.2082590	0.0906090
C	3.2861530	-0.0415090	0.0501380
C	3.8385460	1.2428220	0.1937340
C	4.1493890	-1.1334840	-0.1292330
C	5.2160230	1.4234510	0.1581240
H	3.1797480	2.0902510	0.3325680
C	5.5268790	-0.9467810	-0.1639400
H	3.7525410	-2.1359540	-0.2431640
C	6.0667990	0.3313800	-0.0206230
H	5.6279690	2.4198760	0.2703090
H	6.1790060	-1.8011080	-0.3033320
H	7.1407500	0.4752410	-0.0479590
N	0.9816320	0.7789300	0.2492280

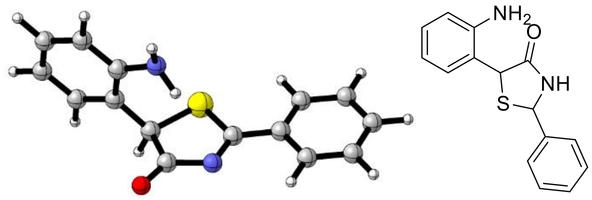

(**17**)

31

1-10 0 1 scf done:-1162.081041 Sum of electronic and thermal Free Energies -1161.889224

imag. freq.: 0

C	-4.3397410	-1.4747780	-0.6120430
C	-3.0652460	-1.9431320	-0.3136700
C	-2.0141260	-1.0759440	0.0051300
C	-2.2526630	0.3179760	0.0255660
C	-3.5451960	0.7812980	-0.2787720
C	-4.5711090	-0.0980850	-0.5941610
H	-5.1360380	-2.1687280	-0.8524810
H	-2.8669310	-3.0095370	-0.3243890
H	-3.7287260	1.8506050	-0.2621780
H	-5.5563260	0.2936010	-0.8219610
N	-1.2477060	1.2461970	0.2675150
H	-1.5800890	2.1745150	0.4955200
C	-0.0272020	-1.2844970	1.6554660
O	-0.6279760	-1.3963060	2.7027410
C	-0.6674630	-1.6747020	0.3015580
S	0.6281050	-1.3019050	-0.9563850
C	1.7239630	-0.7785880	0.3303610
C	3.0716120	-0.3109700	0.0033470
C	3.9443330	0.0527200	1.0426870
C	3.5080050	-0.2175950	-1.3268390
C	5.2256890	0.5008520	0.7512460
H	3.6068560	-0.0221430	2.0678520
C	4.7909390	0.2320090	-1.6127720
H	2.8501660	-0.4911570	-2.1434360
C	5.6515570	0.5916260	-0.5752390
H	5.8947620	0.7788880	1.5569580
H	5.1191840	0.3021900	-2.6427850
H	6.6525920	0.9414740	-0.7998990
N	1.2798060	-0.8213800	1.5536910
H	-0.4797420	0.9796930	0.8663760
H	-0.7633440	-2.7617330	0.3231490

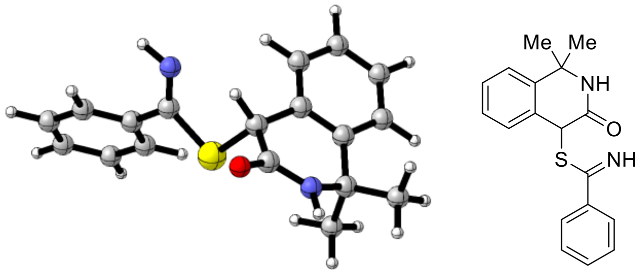

(6a''')

2b-1 0 1 scf done: -1280.076091 Sum of electronic and thermal Free Energies -1279.803591
imag. freq.: 0

C	2.4378870	2.7956400	0.7848470
C	1.7254670	1.6225960	1.0018120
C	1.9755190	0.4848980	0.2256320
C	2.9662250	0.5124090	-0.7627760
C	3.6710910	1.7014060	-0.9790100
C	3.4093340	2.8355900	-0.2161200
H	2.2389280	3.6724220	1.3905000
H	0.9612650	1.5776940	1.7690290
H	4.4329700	1.7475740	-1.7465410
H	3.9692950	3.7459870	-0.3976130
N	2.8106070	-1.9314860	-0.8803310
H	3.2459420	-2.8001970	-1.1690670
C	1.8481350	-2.0466980	0.0540700
O	1.5437910	-3.1183820	0.5766000
C	1.1548010	-0.7522200	0.4441420
H	0.8403620	-0.8401420	1.4829410
S	-0.4540880	-0.6760120	-0.4928610
C	-1.5939300	-0.1712240	0.7941480
C	-2.9643440	0.0872640	0.2721260
C	-4.0672860	-0.3754770	1.0024890
C	-3.1739620	0.7959650	-0.9173370
C	-5.3593480	-0.1310480	0.5486710
H	-3.9096840	-0.9391300	1.9146860
C	-4.4699480	1.0465940	-1.3620560
H	-2.3293140	1.1707310	-1.4821160
C	-5.5636530	0.5820810	-0.6332850
H	-6.2064050	-0.5012620	1.1145880
H	-4.6236030	1.6076430	-2.2765330
H	-6.5711360	0.7736710	-0.9843970
N	-1.2285670	-0.0923560	2.0097760
H	-2.0010880	0.2176170	2.6016850
C	3.2330740	-0.7216560	-1.6162210
C	4.7268280	-0.9054750	-1.9160770
H	4.8780720	-1.8495080	-2.4468500
H	5.0999890	-0.1046940	-2.5544380
H	5.3095120	-0.9245440	-0.9925030
C	2.4457200	-0.6228120	-2.9383330
H	2.6058790	-1.5243730	-3.5357350
H	1.3752840	-0.5153240	-2.7510800
H	2.7818660	0.2434480	-3.5126280

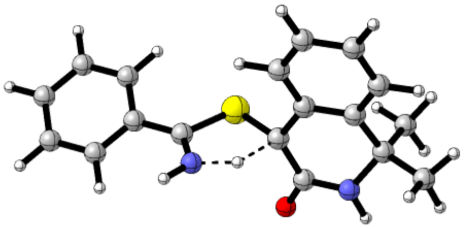

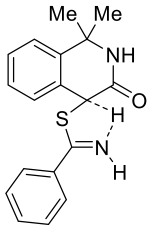

40

2b-TS1 0 1 scf done:-1280.042945 Sum of electronic and thermal Free Energies -1279.773908 imag. freq.: 1 (-1568.6)

C	2.5776420	2.8475330	0.3404490
C	1.6646540	1.8079600	0.2089760
C	2.0886270	0.4797820	0.0293020
C	3.4712900	0.2126860	-0.0035200
C	4.3794120	1.2659430	0.1331180
C	3.9457620	2.5786930	0.3036830
H	2.2222530	3.8633010	0.4754920
H	0.6028500	2.0236230	0.2429720
H	5.4430050	1.0655540	0.0923470
H	4.6683580	3.3804500	0.4050640
N	2.9331910	-2.1637730	0.1713940
H	3.2317230	-3.1324310	0.1416430
C	1.5884240	-2.0143310	-0.0616990
O	0.8476520	-3.0026460	-0.1438440
C	1.1039260	-0.6256620	-0.0516590
H	0.2985340	-0.5777860	1.1598540
S	-0.3753900	-0.3452500	-1.0161340
C	-1.4818950	-0.1935090	0.3871450
C	-2.9049720	0.0784700	0.1650970
C	-3.8569260	-0.3626670	1.0970970
C	-3.3261130	0.7741250	-0.9769380
C	-5.2068870	-0.1095180	0.8860630
H	-3.5430040	-0.9190850	1.9720930
C	-4.6778330	1.0332100	-1.1769510
H	-2.5990480	1.1321780	-1.6953230
C	-5.6193840	0.5906480	-0.2486550
H	-5.9379200	-0.4614320	1.6042990
H	-4.9950880	1.5816200	-2.0559020
H	-6.6728560	0.7891420	-0.4090540
N	-0.8946790	-0.3706610	1.5106680
H	-1.3895060	-0.2766790	2.3916760
C	3.9566140	-1.2036450	-0.2889280
C	5.2485670	-1.5545100	0.4567560
H	5.4840990	-2.6109090	0.2978940
H	6.0929940	-0.9707100	0.0889680
H	5.1386020	-1.3807590	1.5297170
C	4.1680520	-1.3637940	-1.8092530
H	4.9375400	-0.6742070	-2.1654400
H	4.4814470	-2.3865120	-2.0403780
H	3.2396050	-1.1546040	-2.3471890

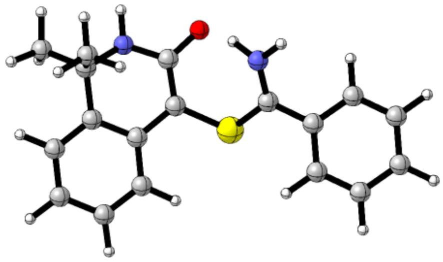

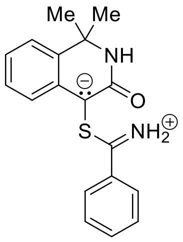

(6a'')

40

2b-2 0 1 scf done: -1280.055151 Sum of electronic and thermal Free Energies -1279.781381 imag. freq.: 0

C	-3.1232660	-2.7796130	-0.0936630
C	-2.0594680	-1.8972450	-0.2482360
C	-2.2177340	-0.5063950	-0.0641970
C	-3.5064930	-0.0351210	0.3067860
C	-4.5589260	-0.9345610	0.4545260
C	-4.3865110	-2.3055070	0.2530530
H	-2.9598690	-3.8411460	-0.2468190
H	-1.0897030	-2.2969880	-0.5178780
H	-5.5352710	-0.5671550	0.7463330
H	-5.2217530	-2.9854460	0.3746100
N	-2.7466670	2.2194560	-0.1780340
H	-2.8393170	3.2221980	-0.0624560
C	-1.4296150	1.8396520	-0.3524860
O	-0.5727500	2.7402930	-0.6127430
C	-1.1439900	0.4594610	-0.2439750
S	0.4221390	-0.1697710	-0.7786180
C	1.6599970	0.5924810	0.1520110
C	2.9989320	-0.0134700	0.1274990
C	4.1360740	0.8105820	0.1283910
C	3.1543470	-1.4081600	0.1131800
C	5.4051850	0.2451690	0.1165060
H	4.0264430	1.8885260	0.1098800
C	4.4273940	-1.9659930	0.1032900
H	2.2829040	-2.0499990	0.1369410
C	5.5531370	-1.1423520	0.1040330
H	6.2788240	0.8860640	0.1069600
H	4.5410230	-3.0434790	0.1028550
H	6.5444270	-1.5805230	0.0948160
N	1.4234970	1.7167720	0.7848510
H	2.1623010	2.1470920	1.3302240
C	-3.6745700	1.4386360	0.6606750
H	0.6213120	2.2946640	0.4444670
C	-5.0809050	1.9763290	0.3814640
H	-5.3765920	1.7811470	-0.6519460
H	-5.8193440	1.5320560	1.0502310
H	-5.0946350	3.0575610	0.5477650
C	-3.3313030	1.6377450	2.1534860
H	-3.4064560	2.6971560	2.4186130
H	-4.0219350	1.0709260	2.7830900
H	-2.3137730	1.2987840	2.3639910

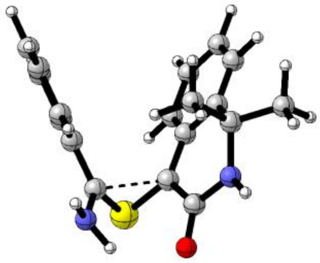 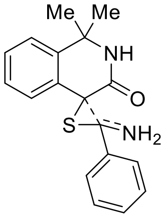

40

2b-TS2 0 1 scf done: -1280.047399 Sum of electronic and thermal Free Energies -1279.772556 imag. freq.: 1 (-227.2)

C	-0.6565730	2.9104850	-1.4164800
C	-0.1273580	1.6269130	-1.4896760
C	0.6787320	1.1098470	-0.4600650
C	0.9594130	1.9342310	0.6566020
C	0.4192140	3.2165970	0.7156500
C	-0.3901870	3.7105400	-0.3084270
H	-1.2754180	3.2825620	-2.2254850
H	-0.3309380	1.0134200	-2.3566100
H	0.6236580	3.8465910	1.5722460
H	-0.8011360	4.7112010	-0.2389170
N	2.7265320	0.3759880	1.2617420
H	3.4359210	0.0603260	1.9130280
C	2.3858620	-0.5694430	0.3306910
O	3.0380260	-1.6247120	0.2326400
C	1.2374660	-0.2363820	-0.5022500
S	0.8106310	-1.3880970	-1.8062900
C	0.0080310	-1.8930800	-0.3151190
C	-1.3363860	-1.4079190	0.0495350
C	-2.1753420	-0.8150640	-0.9048000
C	-1.8019370	-1.5255000	1.3717850
C	-3.4364450	-0.3453760	-0.5487730
H	-1.8437860	-0.7378620	-1.9304440
C	-3.0609310	-1.0539950	1.7227990
H	-1.1810410	-1.9653630	2.1424810
C	-3.8838430	-0.4586760	0.7651330
H	-4.0691910	0.1073830	-1.3034340
H	-3.3977180	-1.1451740	2.7490600
H	-4.8646060	-0.0899750	1.0427840
N	0.5433770	-2.9566210	0.3426890
H	0.2264290	-3.1230170	1.2904700
C	1.7859020	1.3721950	1.8062440
H	1.5484930	-3.0661530	0.2245610
C	2.6338700	2.4350930	2.5121090
H	3.2534260	2.9809510	1.7970940
H	2.0091360	3.1453040	3.0546290
H	3.2890300	1.9492820	3.2408140
C	0.8440440	0.6940900	2.8236590
H	0.1449040	1.4224380	3.2419630
H	0.2699420	-0.0974370	2.3404970
H	1.4268850	0.2571250	3.6401650

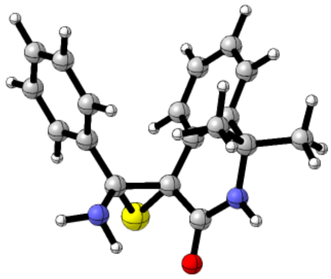

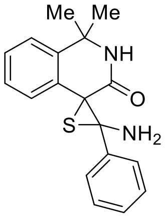

40

2b-3 0 1 scf done:-1280.069908 Sum of electronic and thermal Free Energies -1279.794157
imag. freq.: 0

C	0.6960690	2.6389950	1.3881670
C	0.2484900	1.3251580	1.3483770
C	-0.6478490	0.8938240	0.3626200
C	-1.1338730	1.8216130	-0.5707610
C	-0.6674650	3.1403560	-0.5286960
C	0.2460520	3.5506480	0.4349980
H	1.3931120	2.9475520	2.1588690
H	0.5949940	0.6155370	2.0861600
H	-1.0244480	3.8582860	-1.2557120
H	0.5921080	4.5779000	0.4491070
N	-2.8468400	0.2049890	-1.1935830
H	-3.6971120	-0.0106390	-1.7024330
C	-2.3790740	-0.8080750	-0.4421760
O	-2.9945860	-1.8681000	-0.3275220
C	-1.0406510	-0.5560130	0.2451950
S	-0.7067550	-1.6647190	1.6898890
C	0.0341790	-1.5733670	-0.0749500
C	1.4701880	-1.1222730	-0.1595450
C	2.3869950	-1.3317330	0.8689630
C	1.8934220	-0.4867460	-1.3322070
C	3.7054960	-0.8962970	0.7392210
H	2.0655120	-1.8239450	1.7779530
C	3.2059580	-0.0411540	-1.4581870
H	1.1917630	-0.3314100	-2.1411000
C	4.1167140	-0.2427780	-0.4203820
H	4.4075040	-1.0649850	1.5479840
H	3.5167160	0.4642650	-2.3656240
H	5.1390030	0.1049800	-0.5171920
N	-0.2611020	-2.5847950	-1.0096390
H	0.4050350	-3.3482440	-0.9625010
C	-2.1297570	1.4087620	-1.6428770
H	-1.2101060	-2.9357930	-0.9219980
C	-1.3983860	1.0959070	-2.9624520
H	-0.8401550	1.9697190	-3.3056510
H	-0.7041320	0.2661400	-2.8244990
H	-2.1233860	0.8176720	-3.7324800
C	-3.2024340	2.4804810	-1.8828620
H	-3.6855400	2.7656070	-0.9458350
H	-2.7752380	3.3691900	-2.3469380
H	-3.9619530	2.0855760	-2.5633840

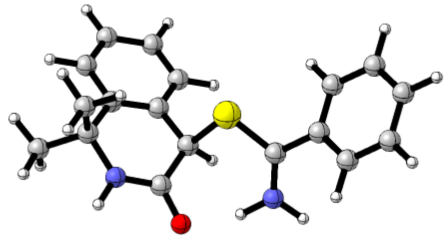

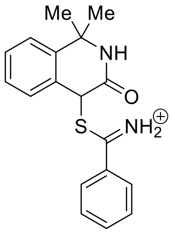

(6a)

41

2b-4 1 1 scf done: -1280.541116 Sum of electronic and thermal Free Energies -1280.253915

imag. freq.: 0

C	2.3761160	2.7609300	1.4648070
C	1.6317290	1.6349250	1.7847760
C	1.8006610	0.4446280	1.0660380
C	2.7349470	0.3699190	0.0282310
C	3.4710020	1.5167860	-0.2926200
C	3.2931600	2.7004540	0.4138370
H	2.2440090	3.6782510	2.0261570
H	0.9099280	1.6681260	2.5933790
H	4.1906060	1.4862910	-1.1003590
H	3.8764090	3.5749540	0.1497080
N	2.3947490	-2.0655830	-0.0178330
H	2.7031130	-2.9783510	-0.3378980
C	1.4836480	-2.0778290	0.9494050
O	1.0653370	-3.1254610	1.4729080
C	0.9469550	-0.7388830	1.4124690
S	-0.7439810	-0.4388760	0.6426950
C	-1.8377180	-1.4315760	1.5633270
C	-3.2598040	-1.0961250	1.4598420
C	-4.2063710	-2.1345950	1.4357170
C	-3.6822160	0.2396190	1.3693760
C	-5.5570040	-1.8339750	1.3260730
H	-3.8829210	-3.1680610	1.4683950
C	-5.0372950	0.5289050	1.2690950
H	-2.9599360	1.0445860	1.4113620
C	-5.9737510	-0.5042200	1.2450890
H	-6.2842460	-2.6361500	1.2941050
H	-5.3623670	1.5607190	1.2151070
H	-7.0294070	-0.2740070	1.1609010
N	-1.4277420	-2.4418550	2.2748100
H	-2.0871820	-2.9446970	2.8614830
H	0.7646370	-0.7882410	2.4860730
C	2.9429020	-0.9122860	-0.7633570
H	-0.4570510	-2.8039530	2.1829270
C	2.2229820	-0.8199120	-2.1222000
H	2.6410170	-0.0008830	-2.7106330
H	2.3541980	-1.7524740	-2.6770070
H	1.1539560	-0.6408890	-1.9874070
C	4.4333330	-1.2163530	-0.9731860
H	4.8981740	-0.4675740	-1.6139150
H	4.9602800	-1.2434650	-0.0172000
H	4.5406950	-2.1879080	-1.4628410

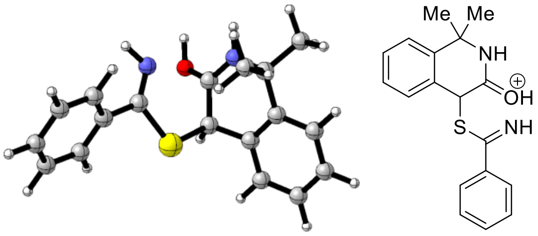

(**6a'**)

41

2b-5 1 1 scf done: -1280.499128 Sum of electronic and thermal Free Energies -1280.212620

imag. freq.: 0

C	3.8692850	-2.3501780	-0.2839330
C	2.9111700	-1.5869560	-0.9408080
C	2.3654690	-0.4604190	-0.3207220
C	2.7986110	-0.0678130	0.9499260
C	3.7591320	-0.8442160	1.6023320
C	4.2852750	-1.9806440	0.9944440
H	4.2891930	-3.2250660	-0.7658570
H	2.5775670	-1.8623880	-1.9351260
H	4.1039890	-0.5646830	2.5889110
H	5.0296760	-2.5702040	1.5167320
N	1.7068400	2.0882230	0.5517590
H	1.6772460	3.0729200	0.8126170
C	1.2517820	1.7306050	-0.6032450
O	0.8676510	2.5759890	-1.5271870
C	1.2751790	0.3028760	-1.0208960
S	-0.3479780	-0.6257840	-0.7495860
C	-1.5957050	0.5306840	-0.1853780
C	-2.9318240	-0.1203590	-0.1059650
C	-3.7121950	0.0894100	1.0391640
C	-3.4265600	-0.9175200	-1.1454910
C	-4.9725250	-0.4900260	1.1392030
H	-3.3231520	0.6914760	1.8519710
C	-4.6932990	-1.4873600	-1.0429490
H	-2.8373160	-1.0724570	-2.0408540
C	-5.4656120	-1.2782190	0.0983910
H	-5.5675610	-0.3317270	2.0310890
H	-5.0764390	-2.0918740	-1.8566660
H	-6.4485090	-1.7282290	0.1780980
N	-1.3067500	1.7148700	0.1648530
H	-2.1406110	2.2252430	0.4613510
H	1.4117550	0.2807560	-2.1005090
C	2.2133890	1.1682630	1.6178640
H	0.7886900	3.4871450	-1.1882650
C	1.0412690	0.7941040	2.5399680
H	0.5794060	1.6951370	2.9494420
H	0.2844920	0.2168980	2.0094630
H	1.4258630	0.1894720	3.3631180
C	3.2625450	1.9809200	2.3823250
H	3.6232790	1.4174010	3.2415320
H	4.1084690	2.2319330	1.7395430
H	2.8112560	2.9034560	2.7567500

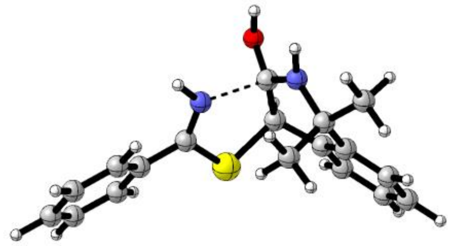

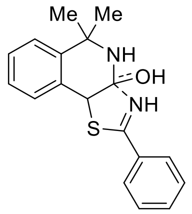

41

2b-TS4 1 1 scf done: -1280.494112 Sum of electronic and thermal Free Energies -1280.207648 imag. freq.: 1 (-244.7)

C	-4.0602250	-2.0699280	0.2786080
C	-3.0433570	-1.3949620	0.9444040
C	-2.3476710	-0.3627430	0.3092180
C	-2.6911810	0.0268070	-0.9902540
C	-3.7094530	-0.6629340	-1.6525920
C	-4.3848340	-1.7080800	-1.0279300
H	-4.5949620	-2.8706000	0.7757940
H	-2.7804990	-1.6708600	1.9600240
H	-3.9856670	-0.3828830	-2.6607610
H	-5.1734520	-2.2294480	-1.5580010
N	-1.4883130	2.0906690	-0.5749710
H	-1.4167110	3.0709400	-0.8287110
C	-0.9125640	1.6979120	0.5515190
O	-0.7872270	2.5667000	1.5630850
C	-1.1856960	0.2751770	1.0296520
S	0.2776530	-0.8817830	0.9103560
C	1.4902040	0.2810990	0.3674300
C	2.8611950	-0.2215350	0.1523810
C	3.6381650	0.3319630	-0.8747970
C	3.3915780	-1.2402930	0.9543750
C	4.9316650	-0.1281570	-1.0912130
H	3.2232000	1.1024020	-1.5136190
C	4.6904630	-1.6894390	0.7377050
H	2.8056840	-1.6648450	1.7608200
C	5.4606320	-1.1364670	-0.2844020
H	5.5254730	0.2959940	-1.8921800
H	5.1003180	-2.4690290	1.3686700
H	6.4705460	-1.4914260	-0.4540920
N	1.0627560	1.4580950	0.1470840
H	1.7219570	2.1699380	-0.1649580
H	-1.3973150	0.3484980	2.0950090
C	-1.9516850	1.1814730	-1.6546450
H	-0.7813060	3.4843350	1.2414270
C	-2.8689620	2.0361040	-2.5348900
H	-3.2034660	1.4693200	-3.4026160
H	-3.7415590	2.3784540	-1.9748330
H	-2.3172910	2.9068600	-2.8997810
C	-0.7625870	0.6627880	-2.4830030
H	-0.1788380	1.5011050	-2.8704290
H	-0.1081930	0.0180110	-1.8969790
H	-1.1438910	0.0808110	-3.3243800

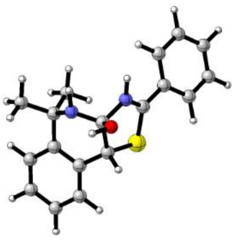

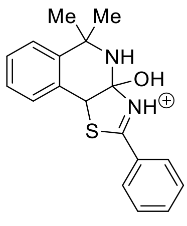

(**7a·H**$^+$)

41

2b-6 1 1 scf done: -1280.512077 Sum of electronic and thermal Free Energies -1280.221708
imag. freq.: 0

C	-3.8533770	-2.4462310	-0.1681520
C	-2.7549860	-2.1172200	0.6176230
C	-2.0870670	-0.9031820	0.4287380
C	-2.5384060	0.0127740	-0.5308470
C	-3.6391600	-0.3327970	-1.3184820
C	-4.2878070	-1.5531640	-1.1466470
H	-4.3661420	-3.3889590	-0.0179180
H	-2.4047190	-2.8053150	1.3798100
H	-4.0026490	0.3561180	-2.0697380
H	-5.1409700	-1.7991100	-1.7684510
N	-1.3023690	1.7221240	0.6534020
H	-0.9533610	2.6769850	0.6586360
C	-0.4423740	0.8614690	1.3783830
O	-0.4145240	1.2610160	2.7279960
C	-0.8478960	-0.6355360	1.2318090
S	0.6131590	-1.5259640	0.4669610
C	1.5957760	-0.1070840	0.4987340
C	2.9564450	-0.0949240	-0.0177980
C	3.4948990	1.0867310	-0.5543000
C	3.7251610	-1.2694810	0.0057460
C	4.7899090	1.0869150	-1.0537820
H	2.8977720	1.9883320	-0.6118260
C	5.0230040	-1.2547660	-0.4869130
H	3.3239100	-2.1808940	0.4327740
C	5.5555670	-0.0794420	-1.0171710
H	5.2006030	1.9949030	-1.4781530
H	5.6190690	-2.1584500	-0.4555510
H	6.5672740	-0.0727250	-1.4054230
N	0.9861800	0.9467150	0.9644440
H	1.4585730	1.8425320	1.0606380
H	-0.9388990	-1.0497480	2.2332510
C	-1.8270280	1.3501970	-0.6831200
H	-1.2944340	1.6155990	2.9272870
C	-2.7933080	2.4813980	-1.0543940
H	-3.1929400	2.3430110	-2.0588580
H	-3.6211430	2.5359570	-0.3445730
H	-2.2569100	3.4344030	-1.0402930
C	-0.7287030	1.2535850	-1.7625480
H	-0.1441560	2.1771100	-1.7869770
H	-0.0524800	0.4144710	-1.5969640
H	-1.1909460	1.1085090	-2.7414840

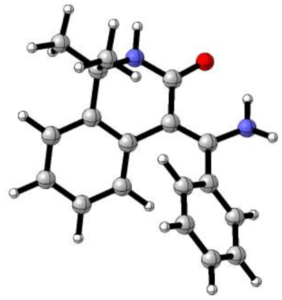

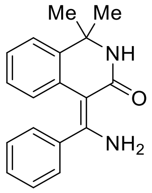

(**9a**)

39

2b-7 0 1 scf done:-881.867664 Sum of electronic and thermal Free Energies -881.591334

imag. freq.: 0

C	0.1372490	2.6597410	1.6071870
C	0.3601060	1.3430140	1.2230210
C	-0.5771070	0.6407680	0.4444830
C	-1.7941530	1.2796100	0.1299310
C	-2.0096870	2.6014090	0.5211930
C	-1.0461570	3.3020660	1.2440750
H	0.8817920	3.1787100	2.2008880
H	1.2707460	0.8494280	1.5350740
H	-2.9384010	3.0949850	0.2635010
H	-1.2285420	4.3293010	1.5386980
N	-2.7382820	-0.9113690	-0.2313530
H	-3.5365420	-1.4950860	-0.4514470
C	-1.5607730	-1.5904670	-0.1201450
O	-1.5542200	-2.8396320	-0.1390010
C	-0.3630520	-0.7559590	0.0219390
C	0.8808590	-1.2957730	-0.2672860
C	2.0870620	-0.4606780	-0.5131650
C	3.3050230	-0.7759280	0.0998220
C	2.0263110	0.6194380	-1.4018170
C	4.4381960	-0.0045610	-0.1506300
H	3.3582140	-1.6091500	0.7908250
C	3.1615410	1.3801690	-1.6602710
H	1.0885770	0.8586290	-1.8878280
C	4.3690100	1.0753160	-1.0295090
H	5.3729570	-0.2462130	0.3423880
H	3.1054060	2.2113720	-2.3539390
H	5.2512250	1.6740540	-1.2258420
N	1.0907590	-2.6288810	-0.3877180
H	1.9012990	-2.9501430	-0.8971030
C	-2.8283300	0.4961400	-0.6678690
H	0.2672140	-3.2241630	-0.3322570
C	-2.5183880	0.6072350	-2.1749860
H	-2.5928640	1.6479580	-2.5000530
H	-1.5090930	0.2492910	-2.3909560
H	-3.2311600	0.0090110	-2.7501690
C	-4.2657890	0.9477930	-0.4006360
H	-4.4899270	0.9354750	0.6684600
H	-4.4413000	1.9523170	-0.7869010
H	-4.9592290	0.2724830	-0.9099810

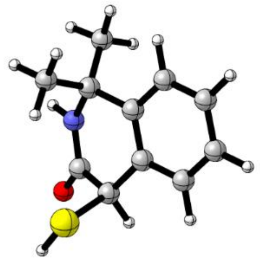

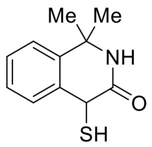

27

2b-8 0 1 scf done: -955.451843Sum of electronic and thermal Free Energies -955.273226
imag. freq.:0

C	-1.9253800	3.1124090	-0.8737930
C	-1.2231770	1.9495930	-1.1635100
C	-1.6301540	0.7189650	-0.6349370
C	-2.7672430	0.6451280	0.1772840
C	-3.4610110	1.8247730	0.4721120
C	-3.0453380	3.0477440	-0.0437270
H	-1.6039110	4.0606500	-1.2888230
H	-0.3461740	1.9853750	-1.8008600
H	-4.3352340	1.7922090	1.1096410
H	-3.5996950	3.9480090	0.1959650
N	-2.6762190	-1.7967600	-0.0396790
H	-3.1366930	-2.6918870	0.0800990
C	-1.5694870	-1.8166880	-0.8038220
O	-1.1721430	-2.8289990	-1.3837720
C	-0.8150610	-0.5078730	-0.9281210
H	-0.3953690	-0.4599870	-1.9309180
S	0.6396510	-0.5493110	0.2494010
C	-3.2242440	-0.6851200	0.7619460
C	-4.7513680	-0.8391820	0.7117720
H	-5.0264630	-1.8415180	1.0516370
H	-5.2421600	-0.1212200	1.3687390
H	-5.1219640	-0.6999540	-0.3062050
C	-2.7336990	-0.8143910	2.2180300
H	-3.0432650	-1.7789560	2.6298620
H	-1.6455320	-0.7461440	2.2665080
H	-3.1623300	-0.0192420	2.8321740
H	1.2000360	-1.6674860	-0.2618540

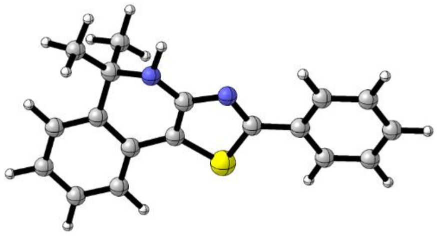

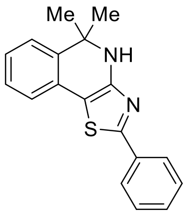

(8a)

37

2b-9 0 1 scf done:-1203.605921 Sum of electronic and thermal Free Energies -1203.355998 imag. freq.: 0

C	-3.8617720	-2.1858190	-0.3180040
C	-2.4874670	-1.9746730	-0.3266770
C	-1.9638000	-0.6732010	-0.3016730
C	-2.8456610	0.4379290	-0.2678920
C	-4.2179860	0.2015190	-0.2469350
C	-4.7308580	-1.0972940	-0.2713670
H	-4.2516540	-3.1971710	-0.3394720
H	-1.8128460	-2.8239140	-0.3426890
H	-4.9073700	1.0360700	-0.2243240
H	-5.8034380	-1.2530830	-0.2588230
N	-0.9699160	1.9090770	0.3110840
H	-0.5448870	2.8292780	0.3120010
C	-0.0926450	0.8802930	0.0689830
C	-0.5506840	-0.3801800	-0.2580200
S	0.8120400	-1.4591840	-0.3997670
C	1.8855400	-0.1133200	-0.0174010
C	3.3393510	-0.2679970	0.0363930
C	4.1349520	0.8203880	0.4326030
C	3.9658090	-1.4787830	-0.2974600
C	5.5179220	0.6952110	0.4924290
H	3.6588310	1.7569040	0.6927750
C	5.3495680	-1.5987320	-0.2348660
H	3.3773780	-2.3332310	-0.6125550
C	6.1320850	-0.5132270	0.1599420
H	6.1190280	1.5430000	0.8009870
H	5.8172870	-2.5407120	-0.4968440
H	7.2107640	-0.6082180	0.2076200
N	1.2589000	1.0204290	0.1979340
C	-2.2795150	1.8613250	-0.3850070
C	-3.1658720	2.9156040	0.2800540
H	-4.1131120	3.0288260	-0.2483130
H	-3.3685410	2.6575350	1.3217910
H	-2.6562830	3.8829870	0.2543780
C	-2.1035400	2.1964570	-1.8809080
H	-1.6807850	3.1996140	-1.9921060
H	-1.4361730	1.4805880	-2.3667640
H	-3.0693830	2.1658560	-2.3912820

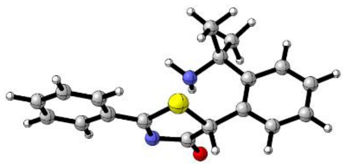

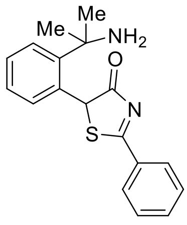

(7a')

40

2b-10 0 1 scf done:-1280.057740 Sum of electronic and thermal Free Energies -1279.783924 imag. freq.: 0

C	4.3057810	1.9984210	0.2969440
C	2.9807770	1.9026760	0.7007210
C	2.1473820	0.8381820	0.3174140
C	2.6776200	-0.1826310	-0.5091470
C	4.0154890	-0.0551280	-0.9131730
C	4.8255070	1.0068510	-0.5263830
H	4.9150020	2.8352550	0.6183720
H	2.5683680	2.6774120	1.3375990
H	4.4439300	-0.8085370	-1.5591700
H	5.8534730	1.0525510	-0.8681970
N	0.7447660	-1.6811310	-0.1882210
H	0.0287160	-2.1820230	-0.7065970
C	0.2272680	-0.1714920	1.7946260
O	0.9125600	-0.6734920	2.6618040
C	0.7415280	0.9386510	0.8679930
S	-0.6151200	1.2110780	-0.3401320
C	-1.6800480	0.2077030	0.6562260
C	-3.0942480	0.0566010	0.2897280
C	-3.8760320	-0.9053180	0.9491020
C	-3.6782480	0.8456320	-0.7113300
C	-5.2134880	-1.0699100	0.6115390
H	-3.4232780	-1.5182010	1.7173470
C	-5.0175040	0.6780450	-1.0454170
H	-3.0955970	1.6010530	-1.2255650
C	-5.7877590	-0.2791620	-0.3853530
H	-5.8093540	-1.8162110	1.1239110
H	-5.4598710	1.2958040	-1.8178510
H	-6.8318440	-0.4086280	-0.6463890
N	-1.1526250	-0.3968680	1.6713090
C	1.8844590	-1.3808510	-1.0685090
C	2.7630690	-2.6429520	-1.2043410
H	3.5491480	-2.5440800	-1.9546640
H	3.2262830	-2.8962620	-0.2462780
H	2.1220460	-3.4752570	-1.5066310
C	1.3466860	-1.0032050	-2.4599020
H	0.7848010	-1.8437130	-2.8803090
H	0.6857760	-0.1376930	-2.4063060
H	2.1727180	-0.7694900	-3.1344100
H	1.0431950	-2.2920740	0.5670070
H	0.7117400	1.8336950	1.4974520

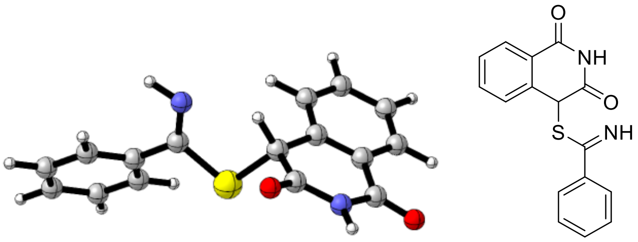

33
3-1 0 1 scf done: -1275.472293 Sum of electronic and thermal Free Energies -1275.272260
imag. freq.: 0

C	2.5333080	2.9038870	0.5322890
C	1.7282450	1.7922590	0.7495400
C	2.0711390	0.5552280	0.1981140
C	3.2355050	0.4490710	-0.5717110
C	4.0405470	1.5734510	-0.7952070
C	3.6903160	2.7977910	-0.2462770
H	2.2611470	3.8572120	0.9703930
H	0.8274610	1.8791140	1.3460410
H	4.9374820	1.4670470	-1.3919030
H	4.3141930	3.6672970	-0.4152130
N	2.8972380	-1.9597900	-0.7437470
H	3.2561800	-2.8642870	-1.0371360
C	1.7849850	-1.9808700	0.0728190
O	1.3098180	-3.0305960	0.4606740
C	1.1804060	-0.6330240	0.4110730
H	0.8217150	-0.6789330	1.4426510
S	-0.3897640	-0.4629460	-0.5677310
C	-1.5920730	-0.2525290	0.7522040
C	-2.9592050	0.0220240	0.2342500
C	-4.0545040	-0.6085160	0.8401470
C	-3.1741770	0.9073580	-0.8295340
C	-5.3450140	-0.3545380	0.3878040
H	-3.8917100	-1.3083020	1.6516470
C	-4.4695400	1.1655050	-1.2713800
H	-2.3373000	1.4126900	-1.2957860
C	-5.5554910	0.5342370	-0.6671870
H	-6.1857830	-0.8537690	0.8553240
H	-4.6288240	1.8627040	-2.0857000
H	-6.5621360	0.7323590	-1.0170480
N	-1.2521830	-0.3667520	1.9700280
H	-2.0438730	-0.2013230	2.5932260
C	3.6570670	-0.8556040	-1.1269580
O	4.6230720	-1.0117760	-1.8542930

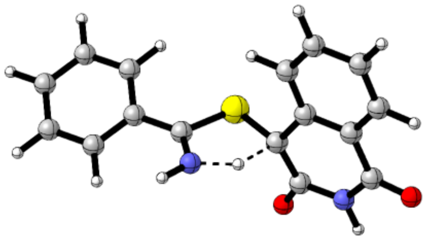
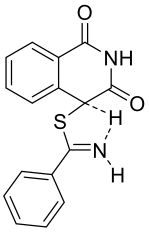

33

3-TS1 0 1 scf done: -1275.451948 Sum of electronic and thermal Free Energies -1275.254321 imag. freq.: 1 (-1617.6)

C	2.5935590	2.8622340	0.0740070
C	1.6630960	1.8507130	-0.1159980
C	2.0539160	0.5006960	-0.1231190
C	3.4183190	0.2089010	0.0822260
C	4.3504010	1.2377420	0.2750750
C	3.9468810	2.5626240	0.2696330
H	2.2634750	3.8951230	0.0719750
H	0.6214520	2.1044600	-0.2663670
H	5.3892530	0.9722780	0.4263600
H	4.6688670	3.3568610	0.4169070
N	2.9238950	-2.1513710	-0.1199800
H	3.2515640	-3.1118170	-0.1473510
C	1.5680210	-1.9713680	-0.3962900
O	0.8806610	-2.9501160	-0.6660060
C	1.0756320	-0.5970260	-0.2475570
H	0.3800020	-0.6398320	0.9532550
S	-0.4489170	-0.2677290	-1.1367240
C	-1.4774090	-0.2150730	0.3328520
C	-2.9092860	0.0676600	0.1894540
C	-3.8179830	-0.4409770	1.1299170
C	-3.3799840	0.8417660	-0.8803980
C	-5.1754510	-0.1750260	0.9993600
H	-3.4650520	-1.0588340	1.9468180
C	-4.7389680	1.1124900	-0.9993170
H	-2.6867720	1.2529200	-1.6041470
C	-5.6377020	0.6038540	-0.0625510
H	-5.8737530	-0.5782690	1.7231200
H	-5.0949180	1.7216700	-1.8215380
H	-6.6969180	0.8123520	-0.1597640
N	-0.8367090	-0.4622090	1.4109230
H	-1.3023640	-0.4282170	2.3131940
C	3.8920890	-1.1868830	0.0979720
O	5.0574300	-1.5191510	0.2855270

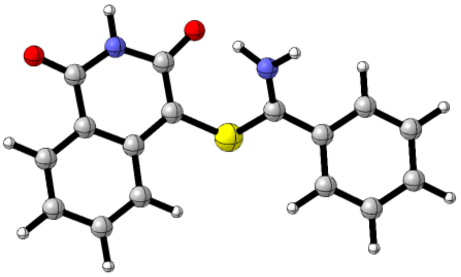
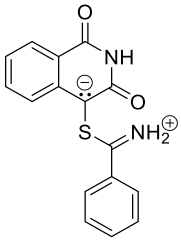

33

3-2 0 1 scf done: -1275.471947 Sum of electronic and thermal Free Energies -1275.269299

imag. freq.: 0

C	-2.9719270	-2.7226180	0.1545880
C	-1.9442480	-1.8254180	-0.0683550
C	-2.1753930	-0.4264970	-0.0708750
C	-3.5076870	0.0081510	0.1801500
C	-4.5351900	-0.9198060	0.4082610
C	-4.2828050	-2.2791170	0.3938200
H	-2.7555430	-3.7854710	0.1497670
H	-0.9438580	-2.2019740	-0.2371700
H	-5.5319580	-0.5401020	0.5970330
H	-5.0802310	-2.9911660	0.5691610
N	-2.7522840	2.2792680	0.0151690
H	-2.9401080	3.2748720	0.0668260
C	-1.4268400	1.9303280	-0.2529640
O	-0.5936940	2.8669710	-0.3891760
C	-1.1470560	0.5515730	-0.3041600
S	0.4247770	-0.0124820	-0.9003250
C	1.6476380	0.6101040	0.1609710
C	2.9721470	-0.0179690	0.1055410
C	4.1227290	0.7805780	0.2152800
C	3.0998410	-1.4061340	-0.0566020
C	5.3803470	0.1933870	0.1658460
H	4.0319800	1.8563300	0.3076620
C	4.3624250	-1.9852720	-0.0993990
H	2.2164970	-2.0288500	-0.1176630
C	5.5018280	-1.1883020	0.0099360
H	6.2659060	0.8132460	0.2387010
H	4.4571390	-3.0585860	-0.2117820
H	6.4849830	-1.6429200	-0.0276130
N	1.4131540	1.6516070	0.9153000
H	2.1319300	1.9864960	1.5483940
C	-3.8278810	1.4376160	0.2188340
H	0.6199020	2.2724480	0.6618360
O	-4.9507160	1.9034230	0.4257390

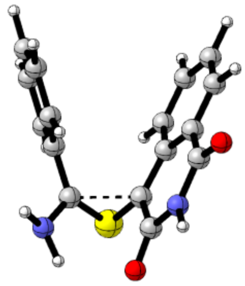

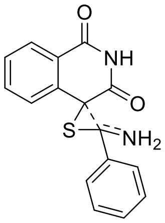

33
3-TS2 0 1 scf done: -1275.454283 Sum of electronic and thermal Free Energies -1275.251284 imag. freq.: 1 (-389.7)

C	-0.8380510	2.7943720	-1.1101950
C	-0.2524870	1.5518590	-1.3001030
C	0.7010780	1.0552460	-0.3938690
C	1.0602760	1.8754610	0.7010220
C	0.4616350	3.1277780	0.8820890
C	-0.4905460	3.5893800	-0.0125790
H	-1.5728510	3.1491410	-1.8241390
H	-0.5335610	0.9521480	-2.1539200
H	0.7631090	3.7259360	1.7328340
H	-0.9542150	4.5578570	0.1316440
N	2.6863540	0.2244060	1.3765510
H	3.4244110	-0.0706400	2.0078940
C	2.4083180	-0.6491480	0.3272250
O	3.0548290	-1.6941290	0.2342730
C	1.2891050	-0.2719140	-0.5156140
S	0.8980160	-1.3424580	-1.9087520
C	0.0798330	-1.7978200	-0.4073810
C	-1.2632620	-1.2680920	-0.0581200
C	-2.2454570	-1.0898240	-1.0340840
C	-1.5383350	-0.9247100	1.2717770
C	-3.4954730	-0.5839770	-0.6833210
H	-2.0333020	-1.3442340	-2.0652160
C	-2.7828990	-0.4092480	1.6164270
H	-0.7684420	-1.0347410	2.0273280
C	-3.7652040	-0.2391490	0.6392610
H	-4.2538190	-0.4525410	-1.4464240
H	-2.9829530	-0.1302710	2.6444930
H	-4.7337710	0.1661770	0.9086520
N	0.5064450	-2.9097960	0.2465160
H	0.1380280	-3.0619130	1.1790500
C	2.0898940	1.4394850	1.6593240
H	1.4860680	-3.1525740	0.1339440
O	2.4384630	2.0819190	2.6446010

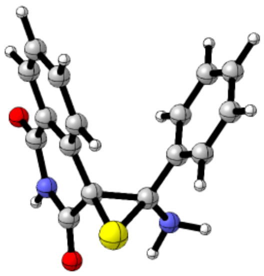 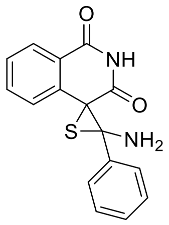

33

3-3 0 1 scf done: -1275.466100 Sum of electronic and thermal Free Energies -1275.262935
imag. freq.: 0

C	0.6621680	2.6381010	1.4686010
C	0.2262870	1.3177610	1.4450780
C	-0.7271700	0.8988210	0.5154530
C	-1.2566400	1.8472380	-0.3744890
C	-0.8109090	3.1733500	-0.3518380
C	0.1529860	3.5700230	0.5630010
H	1.4040150	2.9399110	2.1989070
H	0.6290060	0.6037530	2.1491260
H	-1.2396560	3.8795940	-1.0512200
H	0.4972130	4.5970820	0.5816730
N	-2.8756200	0.2163160	-1.1578190
H	-3.6987130	0.0106250	-1.7177300
C	-2.4697650	-0.7774650	-0.2937750
O	-3.1193420	-1.7994250	-0.1810080
C	-1.1332990	-0.5408770	0.3820370
S	-0.7037670	-1.6991060	1.7443570
C	-0.0663880	-1.5410650	-0.0477250
C	1.3404900	-1.0385410	-0.2279230
C	2.3602790	-1.3263610	0.6756280
C	1.6189460	-0.2708430	-1.3640690
C	3.6494310	-0.8418720	0.4546960
H	2.1445610	-1.9165030	1.5577140
C	2.9030080	0.2197600	-1.5793890
H	0.8252830	-0.0473550	-2.0671180
C	3.9218700	-0.0636470	-0.6683660
H	4.4368250	-1.0687620	1.1643780
H	3.1074780	0.8259220	-2.4545890
H	4.9218940	0.3205660	-0.8346030
N	-0.4027990	-2.5237270	-0.9963520
H	0.3137560	-3.2381040	-1.0663080
C	-2.3286220	1.4845570	-1.3229340
H	-1.3097170	-2.9507710	-0.8405410
O	-2.7746110	2.2288810	-2.1797070

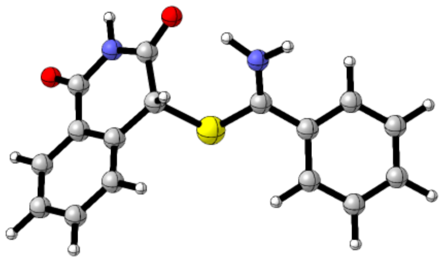

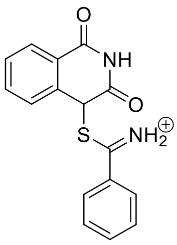

(10a)

34

3-4 1 1 scf done: -1275.930411 Sum of electronic and thermal Free Energies -1275.714494

imag. freq.: 0

C	-2.1546900	1.2765130	-4.0507350
C	-1.4798690	0.5525690	-3.0732940
C	-1.9645900	0.5252480	-1.7656990
C	-3.1329100	1.2280930	-1.4445030
C	-3.8052400	1.9576640	-2.4303390
C	-3.3148560	1.9840420	-3.7287840
H	-1.7764540	1.2874730	-5.0659910
H	-0.5774840	0.0071100	-3.3238200
H	-4.7118940	2.4875320	-2.1683320
H	-3.8372770	2.5467910	-4.4927740
N	-3.0867830	0.2768450	0.8072540
H	-3.5457220	0.1568430	1.7072910
C	-1.9889380	-0.4942180	0.5752660
O	-1.6105210	-1.3362600	1.3806200
C	-1.2188780	-0.2290740	-0.7037840
S	0.2988700	0.7991840	-0.2871050
C	1.4584170	-0.3411530	0.3383260
C	2.8554420	0.0908910	0.3232890
C	3.6962890	-0.2775440	1.3887290
C	3.3580930	0.8733490	-0.7295770
C	5.0214670	0.1326990	1.3935870
H	3.3031810	-0.8508550	2.2193280
C	4.6888730	1.2706240	-0.7172560
H	2.7247660	1.1367400	-1.5667230
C	5.5190260	0.9044160	0.3418220
H	5.6645870	-0.1402400	2.2212670
H	5.0793940	1.8608580	-1.5370910
H	6.5552440	1.2216730	0.3494750
N	1.1097150	-1.4949780	0.8305480
H	1.8210850	-2.1695170	1.0973510
H	-0.8414610	-1.1760280	-1.0894260
C	-3.7086180	1.1638890	-0.0840540
H	0.1257910	-1.7269060	1.0202600
O	-4.6815030	1.7908620	0.2844820

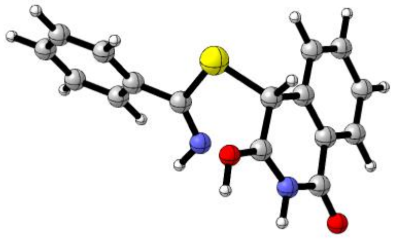

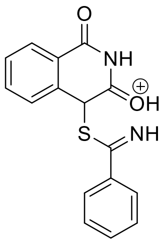

(10a')

34

3-5 1 1 scf done: -1275.884410 Sum of electronic and thermal Free Energies -1275.670400

imag. freq.: 0

C	2.5369150	2.6107440	-0.9540900
C	1.7607410	2.1430060	0.1007830
C	1.7893920	0.7948370	0.4522740
C	2.6304150	-0.0738400	-0.2592140
C	3.4069380	0.4009540	-1.3255800
C	3.3571120	1.7398380	-1.6752730
H	2.5004210	3.6617030	-1.2153140
H	1.1259490	2.8299690	0.6451770
H	4.0415630	-0.2915520	-1.8630640
H	3.9550740	2.1095020	-2.4990850
N	1.9062770	-1.9241930	1.1789310
H	1.9620070	-2.9252990	1.3740550
C	0.9935100	-1.1867230	1.7781340
O	0.2211780	-1.6800440	2.6991540
C	0.9711840	0.3000270	1.6226500
S	-0.7473770	0.9589020	1.6646930
C	-1.4292560	-0.1011740	0.3879090
C	-2.8151840	0.2195010	-0.0217500
C	-3.1972630	0.0175630	-1.3550200
C	-3.7491530	0.7103390	0.8994280
C	-4.4976480	0.3013100	-1.7570430
H	-2.4735360	-0.3417060	-2.0772930
C	-5.0518600	0.9838550	0.4927740
H	-3.4743980	0.8549530	1.9379290
C	-5.4274990	0.7821600	-0.8344270
H	-4.7840870	0.1520030	-2.7914690
H	-5.7725310	1.3510120	1.2137430
H	-6.4410950	1.0007180	-1.1500880
N	-0.6737850	-1.0183240	-0.0586580
H	-1.1180850	-1.6116840	-0.7601360
H	1.4109860	0.6888820	2.5520410
C	2.7362030	-1.4894340	0.0946510
H	0.3129310	-2.6481230	2.7991900
O	3.4457280	-2.3154960	-0.4285750

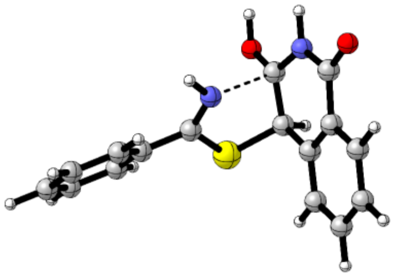 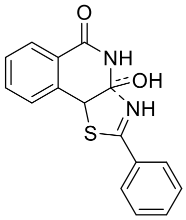

34
3-TS4 1 1 scf done: -1275.884150 Sum of electronic and thermal Free Energies -1275.668418
imag. freq.: 1 (-94.8))

C	2.4315170	-2.8665770	-1.0060540
C	1.7174370	-1.7336800	-1.3808480
C	1.9272390	-0.5195410	-0.7287100
C	2.8870650	-0.4557370	0.2930070
C	3.5988900	-1.6019690	0.6721660
C	3.3687830	-2.8060990	0.0276630
H	2.2540120	-3.8021220	-1.5230620
H	0.9896780	-1.8002730	-2.1792150
H	4.3285240	-1.5270360	1.4679790
H	3.9175050	-3.6929920	0.3197850
N	2.4419660	1.9348720	0.5534350
H	2.6439580	2.7933480	1.0645100
C	1.3702200	1.8792500	-0.2345490
O	0.7268640	2.9734860	-0.5469250
C	1.1710890	0.7167590	-1.1625830
S	-0.6236880	0.4124470	-1.4421510
C	-1.0805710	0.5091650	0.2840970
C	-2.4557630	0.0976800	0.6295250
C	-2.7083820	-0.4702890	1.8861270
C	-3.5083840	0.2678740	-0.2791330
C	-3.9992240	-0.8581660	2.2262500
H	-1.8948040	-0.6281150	2.5845340
C	-4.7996440	-0.1126770	0.0718720
H	-3.3327340	0.7187870	-1.2491170
C	-5.0467220	-0.6774520	1.3223990
H	-4.1868960	-1.3064180	3.1947570
H	-5.6112500	0.0334460	-0.6308580
H	-6.0524370	-0.9790380	1.5912460
N	-0.1663890	0.9186240	1.0663310
H	-0.4187860	1.0209950	2.0491370
H	1.5441650	1.0470310	-2.1403410
C	3.1980390	0.8072990	0.9720670
H	0.9539660	3.7238410	0.0343390
O	4.0353660	0.9700550	1.8311430

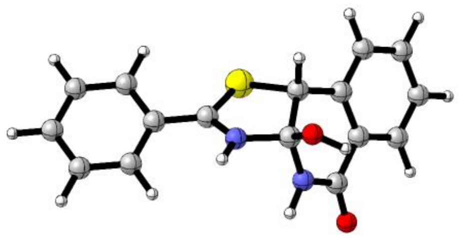 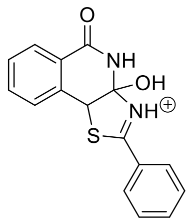

34

3-6 1 1 scf done: -1275.912198 Sum of electronic and thermal Free Energies -1275.694568
imag. freq.: 0

C	4.1292240	2.2539550	-0.2383550
C	2.8779640	2.1089130	0.3550280
C	2.1879790	0.9032580	0.2384070
C	2.7587100	-0.1616420	-0.4708460
C	4.0151440	-0.0115390	-1.0608680
C	4.6970480	1.1947060	-0.9476020
H	4.6627230	3.1922970	-0.1437200
H	2.4334490	2.9289630	0.9074090
H	4.4474380	-0.8460770	-1.5976900
H	5.6721380	1.3087430	-1.4058340
N	0.9373920	-1.6219600	0.2083950
H	0.5068760	-2.5413650	0.1898640
C	0.4245790	-0.6902250	1.1742560
O	0.7392890	-1.0371800	2.4920570
C	0.8269810	0.7554810	0.8348470
S	-0.5184630	1.3392070	-0.3336550
C	-1.6225010	0.1735170	0.3130510
C	-3.0302690	0.1579520	-0.0307670
C	-3.7527440	-1.0493080	-0.0087050
C	-3.6693360	1.3521090	-0.4071920
C	-5.0960820	-1.0511970	-0.3535790
H	-3.2625800	-1.9825480	0.2404080
C	-5.0152520	1.3383190	-0.7428380
H	-3.1250200	2.2884900	-0.4068850
C	-5.7285410	0.1393720	-0.7166480
H	-5.6494600	-1.9821470	-0.3482490
H	-5.5090440	2.2611500	-1.0208170
H	-6.7789160	0.1314340	-0.9831920
N	-1.0463850	-0.7010730	1.1029840
H	-1.5637620	-1.4375170	1.5784400
H	0.7167670	1.3606760	1.7329280
C	2.0593330	-1.4682000	-0.5764560
H	1.6149590	-1.4519140	2.4994740
O	2.4399000	-2.3783480	-1.2972380

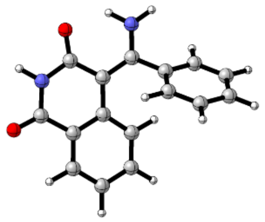

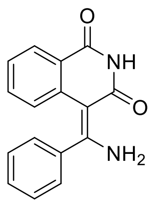

(**11a**)

32

3-7 0 1 scf done:-877.272124 Sum of electronic and thermal Free Energies -877.067220 imag. freq.: 0

C	0.1441310	2.7701430	1.3180030
C	0.2690300	1.4395500	0.9546720
C	-0.7700900	0.7583160	0.2838020
C	-1.9752260	1.4741210	0.0854930
C	-2.0883380	2.8237360	0.4489430
C	-1.0299320	3.4840540	1.0464560
H	0.9660780	3.2562250	1.8318670
H	1.1785740	0.9174310	1.2096230
H	-3.0293900	3.3276190	0.2670860
H	-1.1188680	4.5260760	1.3294480
N	-3.0510960	-0.5741370	-0.5694460
H	-3.8895010	-1.0780450	-0.8404560
C	-1.9077320	-1.3519390	-0.4174320
O	-2.0230430	-2.5780340	-0.5783090
C	-0.6743260	-0.6402670	-0.1380770
C	0.5378020	-1.3133530	-0.3649780
C	1.8373730	-0.6151900	-0.5485290
C	2.9648470	-1.0203530	0.1731690
C	1.9487640	0.4157470	-1.4882790
C	4.1864400	-0.3800720	-0.0239770
H	2.8790890	-1.8150410	0.9051030
C	3.1735680	1.0410490	-1.6936140
H	1.0774670	0.7220480	-2.0539910
C	4.2926150	0.6510790	-0.9558740
H	5.0527080	-0.6860830	0.5511710
H	3.2555520	1.8340770	-2.4279280
H	5.2441020	1.1468620	-1.1108270
N	0.6030980	-2.6409110	-0.5059740
H	1.4501160	-3.0670670	-0.8545430
C	-3.1717020	0.7954850	-0.4347510
H	-0.2660970	-3.1695160	-0.4874870
O	-4.2357190	1.3509620	-0.6921290

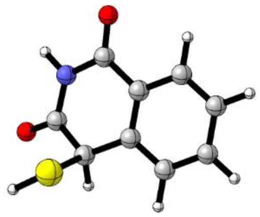

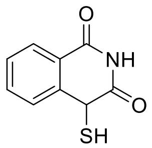

20

3-8 0 1 scf done: -950.848211 Sum of electronic and thermal Free Energies -950.741470
imag. freq.: 0

C	2.6914130	-1.7614030	-0.3458330
C	1.3231910	-1.9333080	-0.5295650
C	0.4527250	-0.8536770	-0.3740420
C	0.9700990	0.4029180	-0.0348900
C	2.3463400	0.5706790	0.1542450
C	3.2046480	-0.5093500	0.0019610
H	3.3601470	-2.6044040	-0.4750070
H	0.9255450	-2.9064330	-0.7939410
H	2.7264200	1.5514900	0.4099100
H	4.2705990	-0.3790620	0.1454440
N	-1.2422220	1.3885740	-0.3122070
H	-1.8193590	2.2241870	-0.3516900
C	-1.8249430	0.2174350	-0.7501450
O	-2.9400070	0.2067800	-1.2370310
C	-1.0278020	-1.0492190	-0.5237950
H	-1.2521470	-1.7461840	-1.3286460
S	-1.6628290	-1.8138570	1.0620770
C	0.0818030	1.5825410	0.0750270
H	-2.9335030	-1.9822610	0.6330330
O	0.4513750	2.6854450	0.4392420

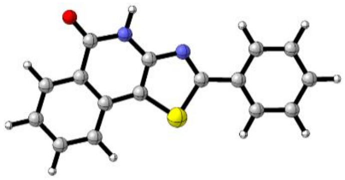 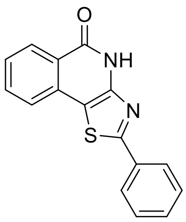

30

3-9 0 1 scf done:-1199.019284 Sum of electronic and thermal Free Energies -1198.840643
imag. freq.: 0

C	-3.7757800	-2.5661330	0.0347520
C	-2.4261440	-2.2622300	0.0288860
C	-1.9988000	-0.9218840	0.0841360
C	-2.9726410	0.1111780	0.1454990
C	-4.3342900	-0.2221740	0.1501250
C	-4.7370990	-1.5457370	0.0954130
H	-4.0910310	-3.6023020	-0.0080300
H	-1.6924020	-3.0588890	-0.0183770
H	-5.0616400	0.5783760	0.1972530
H	-5.7916370	-1.7941780	0.0993550
N	-1.2327030	1.7921190	0.1966590
H	-0.9368680	2.7618710	0.2364150
C	-0.2725240	0.8120330	0.1379970
C	-0.6257710	-0.5196640	0.0824600
S	0.8293410	-1.4778680	0.0176770
C	1.7791750	0.0126730	0.0685930
C	3.2414160	-0.0013220	0.0448180
C	3.9423750	1.2129170	0.1324740
C	3.9641670	-1.1989390	-0.0647510
C	5.3317540	1.2226780	0.1112870
H	3.3897640	2.1395700	0.2175510
C	5.3541300	-1.1828740	-0.0848020
H	3.4490790	-2.1503940	-0.1367930
C	6.0432630	0.0268190	0.0029010
H	5.8616620	2.1657170	0.1798320
H	5.8990750	-2.1156180	-0.1701110
H	7.1269530	0.0374270	-0.0132410
N	1.0553670	1.1048900	0.1303390
C	-2.5924450	1.5392380	0.2054010
O	-3.3992890	2.4661760	0.2598930

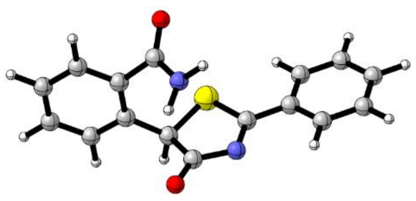 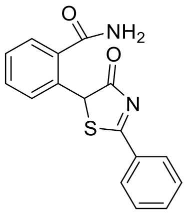

33

3-10 0 1 scf done:-1275.470761 Sum of electronic and thermal Free Energies -1275.270406 imag. freq.: 0

C	-4.4616070	-1.6339420	-0.4595490
C	-3.1402240	-1.9915500	-0.2081320
C	-2.1312620	-1.0279140	-0.1032360
C	-2.4731070	0.3257140	-0.2808360
C	-3.7967220	0.6752710	-0.5593160
C	-4.7925120	-0.2935910	-0.6374660
H	-5.2238010	-2.4014270	-0.5266520
H	-2.8797490	-3.0375980	-0.0931530
H	-4.0439930	1.7200590	-0.7063540
H	-5.8162060	-0.0021670	-0.8413160
N	-0.8325180	1.6068820	0.9513220
H	-0.1951200	2.3841600	1.0641770
C	-0.1154600	-1.1872040	1.5414460
O	-0.7485710	-1.2844100	2.5711800
C	-0.7301840	-1.5265850	0.1635920
S	0.5368520	-1.0139800	-1.0585980
C	1.6639390	-0.6739900	0.2667780
C	3.0275050	-0.2330880	-0.0296610
C	3.8803540	0.1181390	1.0301620
C	3.4973120	-0.1483490	-1.3489500
C	5.1757070	0.5446060	0.7698780
H	3.5151930	0.0545150	2.0465930
C	4.7948210	0.2783750	-1.6035190
H	2.8589170	-0.4193990	-2.1818690
C	5.6356530	0.6255290	-0.5458440
H	5.8284710	0.8149680	1.5914440
H	5.1501270	0.3392820	-2.6251140
H	6.6474610	0.9584400	-0.7464810
N	1.2222320	-0.8151860	1.4798870
C	-1.4773450	1.4573970	-0.2295760
H	-1.1803560	1.1646870	1.7897790
H	-0.7490910	-2.6189590	0.1323860
O	-1.2880620	2.1881470	-1.1968020

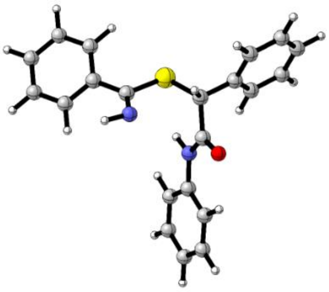 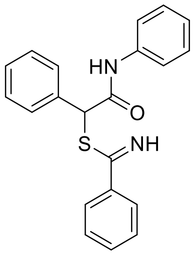

43
4a-1 0 1 scf done: -1394.426737 Sum of electronic and thermal Free Energies -1394.141577
imag. freq.: 0

C	0.3120110	1.2033870	-5.3010780
C	0.6620130	0.1417600	-4.4692300
C	-0.0927840	-0.1415060	-3.3277500
C	-1.2034570	0.6542380	-3.0268210
C	-1.5526390	1.7160090	-3.8587130
C	-0.7962000	1.9934020	-4.9972220
H	0.9029560	1.4109400	-6.1859840
H	1.5240120	-0.4731190	-4.7037580
H	-2.4167340	2.3248610	-3.6181260
H	-1.0700820	2.8187850	-5.6444840
N	-1.4849950	-2.0350420	-0.9329880
C	-0.8735990	-2.2186140	-2.1302940
O	-1.2301960	-3.0125410	-2.9907090
C	0.3175780	-1.2913480	-2.4314170
H	1.0528430	-1.9109900	-2.9404710
S	1.1708620	-0.5703520	-0.9612590
C	1.8837430	-2.0457030	-0.2277140
C	2.6902300	-1.7760410	0.9924620
C	2.6018320	-2.6612150	2.0756420
C	3.5409280	-0.6668460	1.0777260
C	3.3561050	-2.4401070	3.2233550
H	1.9305720	-3.5107530	2.0246130
C	4.3016790	-0.4556410	2.2249590
H	3.6282720	0.0179550	0.2428160
C	4.2091300	-1.3385470	3.2998160
H	3.2739620	-3.1243010	4.0599450
H	4.9680940	0.3974460	2.2767420
H	4.7976560	-1.1688670	4.1941500
N	1.6428620	-3.1832790	-0.7432160
H	2.1258620	-3.9226470	-0.2287390
H	-1.0429120	-1.3643340	-0.3144060
H	-1.8014730	0.4453950	-2.1468510
C	-2.6047440	-2.6944160	-0.3930940
C	-2.9573520	-2.3628240	0.9234880
C	-3.3674330	-3.6342860	-1.0974530
C	-4.0564440	-2.9606620	1.5273990
H	-2.3628510	-1.6337170	1.4644260
C	-4.4672160	-4.2264970	-0.4768870
H	-3.1002400	-3.8932090	-2.1097930
C	-4.8200500	-3.8986610	0.8302710
H	-4.3158190	-2.6927330	2.5453650
H	-5.0522970	-4.9531250	-1.0296900
H	-5.6772990	-4.3656420	1.3009280

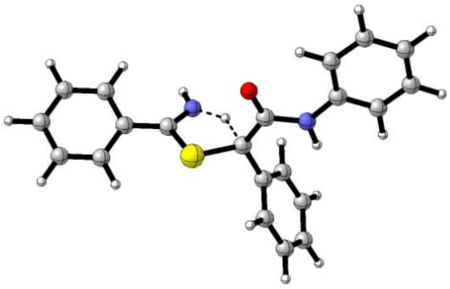

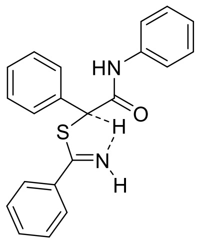

43

4a-TS1 0 1 scf done:-1394.388776 Sum of electronic and thermal Free Energies -1394.108051 imag. freq.: 1 (-1324.8)

C	-3.7877850	-1.2553440	1.4361410
C	-2.7376430	-0.3494350	1.2891870
C	-2.1514730	-0.1152030	0.0355220
C	-2.6477200	-0.8286760	-1.0670410
C	-3.6863660	-1.7447580	-0.9211650
C	-4.2636680	-1.9595160	0.3314860
H	-4.2256390	-1.4171250	2.4150720
H	-2.3650840	0.1901900	2.1533610
H	-4.0522820	-2.2850810	-1.7874890
H	-5.0754100	-2.6694290	0.4438760
N	-2.6860640	2.6528450	-0.0482670
C	-1.3561500	2.3241010	-0.2571580
O	-0.5026720	3.1811140	-0.5051920
C	-1.0483150	0.8916730	-0.0863720
H	-0.2307920	0.8570700	1.1668220
S	0.3911400	0.3516280	-1.0029630
C	1.4597880	0.1760940	0.4229540
C	2.8341250	-0.3009810	0.2477580
C	3.8368090	0.1050560	1.1420090
C	3.1591520	-1.1583850	-0.8129620
C	5.1415530	-0.3428410	0.9744240
H	3.6007670	0.7851440	1.9517080
C	4.4649050	-1.6113050	-0.9684650
H	2.3900940	-1.4887800	-1.5002110
C	5.4573410	-1.2034810	-0.0779480
H	5.9131220	-0.0182360	1.6624930
H	4.7063570	-2.2834790	-1.7832230
H	6.4753330	-1.5538850	-0.2037180
N	0.9021620	0.5285920	1.5219170
H	1.3786920	0.4506570	2.4141050
H	-3.3168640	1.8758380	0.0952680
H	-2.2178210	-0.6599710	-2.0483510
C	-3.2877160	3.9130330	0.0494630
C	-4.6703910	3.9369250	0.3048900
C	-2.6016900	5.1296640	-0.0887420
C	-5.3493950	5.1432400	0.4200520
H	-5.2033780	2.9977580	0.4130440
C	-3.2977930	6.3323430	0.0313880
H	-1.5421800	5.1204740	-0.2891640
C	-4.6677500	6.3546140	0.2848780
H	-6.4156520	5.1359920	0.6178190
H	-2.7532170	7.2641350	-0.0779170
H	-5.1966330	7.2962720	0.3746860

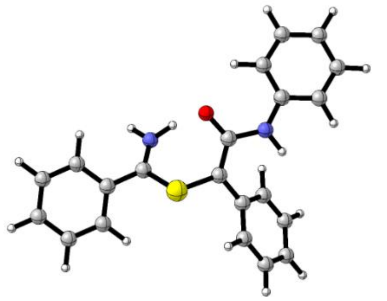

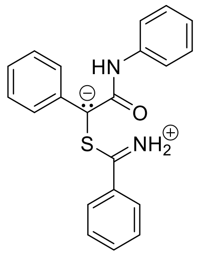

43

4a-2 0 1 scf done:-1394.398170 Sum of electronic and thermal Free Energies -1394.112684 imag. freq.: 0

C	-3.3047010	-2.2261160	-0.7126570
C	-2.2577600	-1.3745490	-1.0544710
C	-2.1318220	-0.0930480	-0.4800620
C	-3.1172260	0.2861040	0.4577350
C	-4.1764000	-0.5571490	0.7799900
C	-4.2787700	-1.8231280	0.2012240
H	-3.3668260	-3.2049950	-1.1762620
H	-1.5310910	-1.7074630	-1.7869110
H	-4.9144080	-0.2296770	1.5046740
H	-5.0985780	-2.4833350	0.4601450
N	-2.4054730	2.7138350	-1.2106900
C	-1.1236160	2.2085070	-0.9947700
O	-0.1363400	3.0033750	-0.9917520
C	-1.0179260	0.8112580	-0.8094920
S	0.5434420	0.0255100	-1.1167740
C	1.6588890	0.5774990	0.1008790
C	2.8713960	-0.2140510	0.3432800
C	4.0826380	0.4349570	0.6342060
C	2.8304380	-1.6156290	0.2888260
C	5.2307380	-0.3102640	0.8707500
H	4.1303190	1.5174310	0.6464670
C	3.9827910	-2.3540580	0.5319220
H	1.8956650	-2.1217090	0.0846450
C	5.1823320	-1.7042520	0.8214960
H	6.1650220	0.1949200	1.0847970
H	3.9432240	-3.4362700	0.5010440
H	6.0797960	-2.2828930	1.0073870
N	1.4412540	1.7127500	0.7098960
H	2.0775110	2.0349980	1.4310530
H	0.7784640	2.3790710	0.2483020
H	-3.1210700	2.0198850	-1.3763710
H	-3.0392110	1.2488340	0.9488260
C	-2.9001940	3.9971730	-0.9712570
C	-2.1098870	5.0931560	-0.5858350
C	-4.2871320	4.1845140	-1.1213570
C	-2.7071160	6.3313100	-0.3546810
H	-1.0439980	4.9655690	-0.4808680
C	-4.8661350	5.4264490	-0.8921320
H	-4.9034450	3.3412660	-1.4161730
C	-4.0811820	6.5143130	-0.5043720
H	-2.0806760	7.1652910	-0.0561060
H	-5.9375430	5.5425630	-1.0143550
H	-4.5319530	7.4835240	-0.3257300

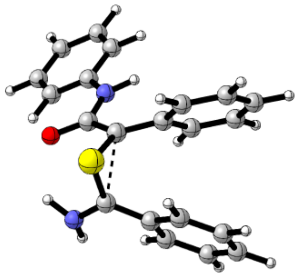

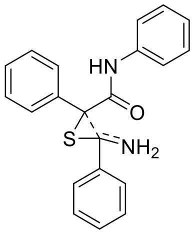

43

4a-TS2 0 1 scf done:-1394.390863 Sum of electronic and thermal Free Energies -1394.102754 imag. freq.: 1 (-57.7)

C	2.0168640	2.5516200	0.7093190
C	0.7840390	2.0258080	1.0813940
C	-0.0402780	1.3732810	0.1482880
C	0.4339070	1.2529160	-1.1729730
C	1.6668290	1.7794810	-1.5444850
C	2.4665170	2.4323790	-0.6063420
H	2.6312500	3.0517040	1.4500290
H	0.4563760	2.1090840	2.1111970
H	2.0127040	1.6604600	-2.5653840
H	3.4316360	2.8337900	-0.8938010
N	-2.5701120	1.7750060	-1.2091640
C	-2.5680650	0.8658840	-0.1604720
O	-3.5876890	0.2124430	0.1279600
C	-1.3103300	0.7542190	0.5529120
S	-1.3600400	-0.1480470	2.1016220
C	-1.1098610	-1.3940560	0.8801700
C	0.2277180	-1.6888220	0.3444680
C	1.3816470	-1.3505700	1.0662450
C	0.3729060	-2.2550610	-0.9338980
C	2.6440390	-1.5752060	0.5281240
H	1.2845690	-0.9055700	2.0469100
C	1.6364510	-2.4804160	-1.4663090
H	-0.4960710	-2.4901300	-1.5365300
C	2.7775650	-2.1396030	-0.7390700
H	3.5241310	-1.3041560	1.0995480
H	1.7299560	-2.9067640	-2.4584150
H	3.7619250	-2.3074590	-1.1606600
N	-2.1732890	-2.1365670	0.5241590
H	-2.1083290	-2.7123790	-0.3069510
H	-3.0792150	-1.6971790	0.6709030
H	-0.1567250	0.7139400	-1.9036610
H	-1.7436610	2.3490900	-1.2984810
C	-3.5701040	2.0533110	-2.1447840
C	-4.7675870	1.3296560	-2.2642880
C	-3.3306390	3.1213300	-3.0293090
C	-5.6907050	1.6792590	-3.2491140
H	-4.9640020	0.5127410	-1.5885140
C	-4.2605030	3.4558420	-4.0052910
H	-2.4065100	3.6837250	-2.9402880
C	-5.4518360	2.7368840	-4.1246920
H	-6.6107890	1.1099780	-3.3277720
H	-4.0528520	4.2831010	-4.6750740
H	-6.1784360	2.9983680	-4.8850280

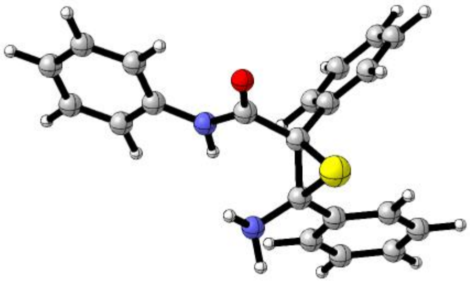

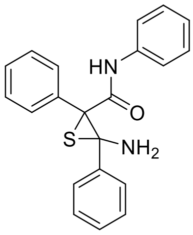

43

4a-3 0 1 scf done:-1394.417909 Sum of electronic and thermal Free Energies -1394.129325 imag. freq.: 0

C	-0.8232820	3.2746560	0.8774150
C	-1.0895370	1.9276800	1.1194390
C	-0.8697170	0.9768470	0.1240460
C	-0.3873210	1.3879230	-1.1234510
C	-0.1144980	2.7319000	-1.3630240
C	-0.3321570	3.6798650	-0.3625820
H	-0.9947830	4.0049030	1.6602660
H	-1.4533680	1.6105840	2.0891290
H	0.2712600	3.0375880	-2.3289970
H	-0.1172290	4.7261610	-0.5481220
N	-2.4823790	-1.1741540	-1.5824420
C	-2.5666980	-0.7856160	-0.2799160
O	-3.6055980	-0.6041480	0.3345870
C	-1.1940900	-0.4835280	0.3452040
S	-0.9169280	-1.1842860	2.0164620
C	-0.0884340	-1.4920360	0.2752100
C	1.3278800	-1.0115930	0.1023180
C	1.9835060	-0.2275180	1.0553150
C	1.9877790	-1.3071710	-1.0980280
C	3.2666250	0.2557120	0.8137300
H	1.4863720	0.0007920	1.9882840
C	3.2723950	-0.8237650	-1.3377250
H	1.4888720	-1.9020880	-1.8515340
C	3.9169160	-0.0386550	-0.3840020
H	3.7595950	0.8603240	1.5666020
H	3.7656700	-1.0595490	-2.2740500
H	4.9166060	0.3372360	-0.5700640
N	-0.3352330	-2.7255710	-0.3526460
H	0.3509370	-3.4286480	-0.0993500
H	-1.2680050	-3.0900640	-0.1971220
H	-1.5494090	-1.3470800	-1.9369360
H	-0.2116220	0.6567510	-1.9031820
C	-3.5160860	-1.4123190	-2.5088920
C	-3.1288230	-1.8421630	-3.7866090
C	-4.8751260	-1.2353500	-2.2207560
C	-4.0855230	-2.0927360	-4.7623680
H	-2.0745730	-1.9740490	-4.0057820
C	-5.8231430	-1.4904200	-3.2113620
H	-5.1792680	-0.9061860	-1.2398590
C	-5.4416590	-1.9185670	-4.4809000
H	-3.7695440	-2.4243550	-5.7449210
H	-6.8731780	-1.3504380	-2.9797330
H	-6.1883710	-2.1138020	-5.2416280

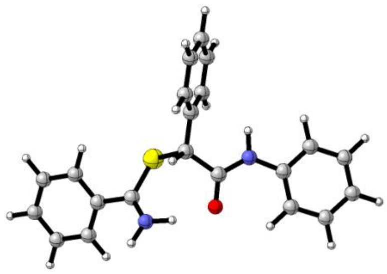

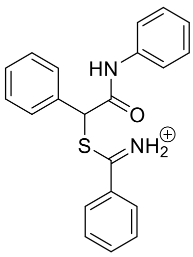

(12a)

44
4a-4 1 1 scf done:-1394.885825 Sum of electronic and thermal Free Energies -1394.584281 imag. freq.: 0

C	-0.1317340	3.6524540	-0.3336650
C	-0.1876480	2.3977490	-0.9351190
C	-0.3340480	1.2481730	-0.1533370
C	-0.4133380	1.3689000	1.2405420
C	-0.3526090	2.6247160	1.8391820
C	-0.2133380	3.7679650	1.0534480
H	-0.0209290	4.5382120	-0.9483120
H	-0.1207340	2.3062150	-2.0132960
H	-0.4133700	2.7076950	2.9179060
H	-0.1667450	4.7448870	1.5205950
N	-2.7481220	-0.2468130	-0.1039800
C	-1.7664090	-0.8295580	-0.8088890
O	-1.8919510	-1.8793290	-1.4504120
C	-0.4172650	-0.0935120	-0.8365640
H	-0.1554720	0.0201050	-1.8895280
S	0.9435800	-1.1527100	-0.1006080
C	1.3877910	-2.2841190	-1.3472110
C	2.7237580	-2.8737070	-1.2370660
C	2.9064190	-4.2234300	-1.5847710
C	3.8134150	-2.1150850	-0.7798250
C	4.1643270	-4.7996300	-1.4788470
H	2.0607730	-4.8210120	-1.9032360
C	5.0700890	-2.6999040	-0.6864090
H	3.6850470	-1.0690420	-0.5340340
C	5.2466220	-4.0392930	-1.0324010
H	4.3002010	-5.8432530	-1.7351110
H	5.9125670	-2.1086440	-0.3489390
H	6.2277610	-4.4926290	-0.9529460
N	0.5669670	-2.6235620	-2.2985440
H	0.8825500	-3.2224830	-3.0561560
H	-2.5114040	0.6250790	0.3553050
H	-0.5281780	0.4874150	1.8627740
C	-4.0873540	-0.6606440	0.0839510
C	-4.8810240	0.1640700	0.8924550
C	-4.6324240	-1.8178410	-0.4823910
C	-6.2088000	-0.1652910	1.1335760
H	-4.4495170	1.0585130	1.3292040
C	-5.9670360	-2.1340780	-0.2305700
H	-4.0264770	-2.4554380	-1.1057460
C	-6.7601850	-1.3181180	0.5725650
H	-6.8122420	0.4800860	1.7613180
H	-6.3850640	-3.0318240	-0.6716240
H	-7.7958420	-1.5755480	0.7607520
H	-0.4349160	-2.3685410	-2.2290950

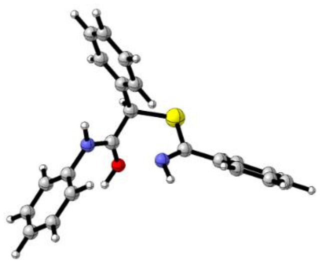

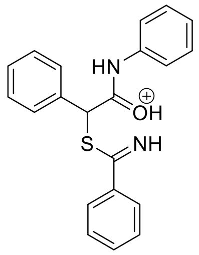

(12a')

44

4a-5 1 1 scf done:-1394.848244 Sum of electronic and thermal Free Energies -1394.549276 imag. freq.: 0

C	-1.9126830	4.3656160	0.2166270
C	-1.4818910	3.3509230	-0.6357710
C	-0.6387820	2.3411430	-0.1632300
C	-0.2282510	2.3569680	1.1742630
C	-0.6602280	3.3718970	2.0235430
C	-1.5025290	4.3773340	1.5480870
H	-2.5633270	5.1454890	-0.1617000
H	-1.7999570	3.3414560	-1.6724940
H	-0.3364030	3.3779070	3.0578880
H	-1.8344170	5.1670620	2.2123400
N	-2.0352640	-0.0986750	-0.1800370
C	-1.0053700	-0.0290740	-0.9746630
O	-0.6864960	-0.9993280	-1.7854420
C	-0.2233070	1.2486850	-1.1246430
S	1.5991550	0.9554580	-1.2312210
C	1.8716000	-0.2906790	0.0238000
C	3.2867110	-0.7287420	0.1264970
C	3.8177820	-0.9991920	1.3949010
C	4.0907950	-0.8922350	-1.0085520
C	5.1339860	-1.4289880	1.5236690
H	3.2047510	-0.8551740	2.2769060
C	5.4051990	-1.3318570	-0.8740210
H	3.6879330	-0.7042120	-1.9964150
C	5.9294750	-1.5983690	0.3897380
H	5.5405170	-1.6267750	2.5086380
H	6.0169530	-1.4697930	-1.7578100
H	6.9546010	-1.9353910	0.4917840
N	0.8933220	-0.6917370	0.7280180
H	1.1697440	-1.4239040	1.3839370
H	-0.4539350	1.5855080	-2.1418050
H	-1.2484120	-1.7879460	-1.6459450
H	-2.1890500	0.6902800	0.4449930
H	0.4197610	1.5763290	1.5501040
C	-2.8392660	-1.2780940	-0.0018540
C	-3.6814270	-1.7012400	-1.0279300
C	-2.7461370	-1.9679470	1.2051390
C	-4.4399980	-2.8556650	-0.8392410
H	-3.7556150	-1.1298700	-1.9457710
C	-3.5181160	-3.1117170	1.3828970
H	-2.0791150	-1.6117420	1.9805870
C	-4.3596810	-3.5566280	0.3621020
H	-5.0996180	-3.1960070	-1.6281630
H	-3.4580380	-3.6579940	2.3164500
H	-4.9553680	-4.4502960	0.5061610

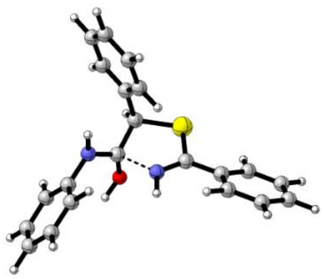 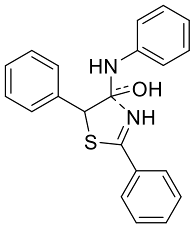

44

4a-TS4 1 1 scf done:-1394.843252 Sum of electronic and thermal Free Energies -1394.543639 imag. freq.: 1 (-294.2)

C	-1.8760280	4.4187140	0.1016840
C	-1.4893010	3.3884290	-0.7534980
C	-0.5953350	2.4062360	-0.3189310
C	-0.0887090	2.4670250	0.9840390
C	-0.4787740	3.4950730	1.8377730
C	-1.3724870	4.4727820	1.3995180
H	-2.5662500	5.1773920	-0.2485800
H	-1.8820710	3.3437360	-1.7633240
H	-0.0811860	3.5339640	2.8452990
H	-1.6708270	5.2738680	2.0659930
N	-2.0332810	-0.0112110	-0.1927620
C	-0.9300710	-0.0373060	-0.9591090
O	-0.8503070	-0.9213230	-1.9491800
C	-0.2429140	1.2889210	-1.2754530
S	1.5769530	1.0328230	-1.4600280
C	1.6982450	-0.2893150	-0.2844840
C	3.0419750	-0.7792430	0.0702640
C	3.2531960	-1.3630800	1.3276430
C	4.1082580	-0.6721120	-0.8327180
C	4.5141820	-1.8353900	1.6711830
H	2.4406250	-1.4274200	2.0416610
C	5.3650560	-1.1562360	-0.4862820
H	3.9588660	-0.2364680	-1.8136820
C	5.5704890	-1.7362120	0.7648580
H	4.6741920	-2.2768510	2.6476680
H	6.1819680	-1.0813340	-1.1939600
H	6.5522440	-2.1077100	1.0347060
N	0.5831130	-0.7056870	0.1732270
H	0.5716500	-1.5038170	0.8056990
H	-0.5872680	1.5581580	-2.2758490
H	-1.4052950	-1.7011850	-1.7649340
H	-2.0360350	0.7010900	0.5301720
H	0.6086400	1.7187630	1.3385970
C	-2.7967580	-1.1941220	0.0846060
C	-3.6719150	-1.6981270	-0.8776480
C	-2.6501570	-1.8238850	1.3214540
C	-4.3918970	-2.8607260	-0.6020260
H	-3.8055740	-1.1756660	-1.8178830
C	-3.3877900	-2.9721280	1.5929550
H	-1.9667330	-1.4128240	2.0552770
C	-4.2522350	-3.4954850	0.6299850
H	-5.0717700	-3.2571950	-1.3466650
H	-3.2821470	-3.4621690	2.5536640
H	-4.8197250	-4.3936220	0.8439360

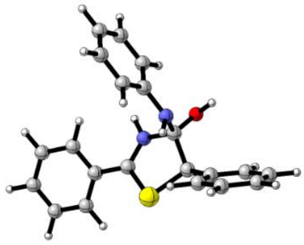

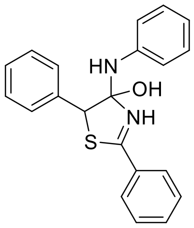

44
4a-6 1 1 scf done:-1394.860797 Sum of electronic and thermal Free Energies -1394.558431 imag. freq.: 0

C	4.9086990	-0.3488880	-0.5424850
C	3.6198970	-0.0776040	-0.9944250
C	2.5147190	-0.3377530	-0.1765080
C	2.7174490	-0.8969460	1.0896660
C	4.0071360	-1.1725300	1.5373330
C	5.1052540	-0.8947880	0.7252200
H	5.7570110	-0.1417790	-1.1844430
H	3.4688990	0.3334860	-1.9862570
H	4.1510030	-1.6087160	2.5188980
H	6.1080690	-1.1120830	1.0743930
N	1.0233490	1.8337950	1.0202120
C	0.6476120	1.4720620	-0.3078430
O	1.0498770	2.4275480	-1.2463680
C	1.1555440	0.0478470	-0.6918360
S	-0.2113950	-1.0984970	-0.1753920
C	-1.3697680	0.1845890	-0.2949740
C	-2.7970950	-0.0404990	-0.1604340
C	-3.6210140	0.9743940	0.3580580
C	-3.3522470	-1.2776000	-0.5278500
C	-4.9830460	0.7498690	0.4954380
H	-3.1946210	1.9142360	0.6862400
C	-4.7174750	-1.4875100	-0.3930210
H	-2.7270050	-2.0577950	-0.9448640
C	-5.5325300	-0.4765300	0.1172210
H	-5.6158910	1.5270450	0.9061230
H	-5.1463940	-2.4368770	-0.6889970
H	-6.5975720	-0.6455660	0.2247720
N	-0.8277990	1.3618840	-0.4732370
H	-1.3913530	2.2010520	-0.5784450
H	1.1558140	0.0247800	-1.7810180
H	1.9041340	2.7769710	-0.9503490
H	1.2751110	1.0431810	1.5998550
H	1.8746430	-1.1289220	1.7315710
C	0.3651800	2.8489130	1.7521350
C	0.2534960	2.6912250	3.1397910
C	-0.1453880	4.0062650	1.1515960
C	-0.3567620	3.6735930	3.9120660
H	0.6467630	1.7935860	3.6052280
C	-0.7709430	4.9756730	1.9338850
H	-0.0530070	4.1616570	0.0849740
C	-0.8794340	4.8210470	3.3142530
H	-0.4319010	3.5350730	4.9847400
H	-1.1665470	5.8633910	1.4532770
H	-1.3610200	5.5827160	3.9159220

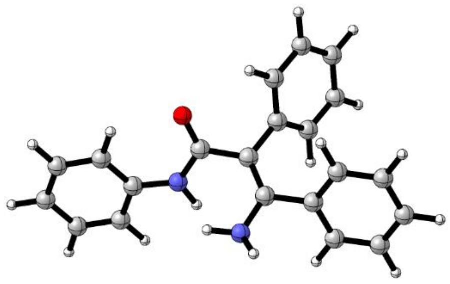

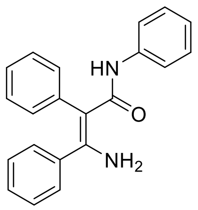

42

4a-7 0 1 scf done:-996.202979 Sum of electronic and thermal Free Energies -995.916555 imag. freq.: 0

C	1.8583330	3.5443710	-0.7219640
C	1.0960100	2.5367850	-0.1331450
C	0.9612060	1.2852320	-0.7516120
C	1.5982950	1.0837700	-1.9860120
C	2.3664640	2.0872450	-2.5705390
C	2.5018070	3.3240920	-1.9396680
H	1.9564240	4.5020540	-0.2221290
H	0.6084050	2.7117970	0.8161280
H	2.8529550	1.9052720	-3.5226650
H	3.0979840	4.1078690	-2.3934840
N	-2.1802420	-0.3766150	-0.3121720
C	-1.2626640	0.5211800	0.1823320
O	-1.5830030	1.5200200	0.8214880
C	0.1575880	0.1893770	-0.1452910
C	0.6862650	-1.0461790	0.0999650
C	2.1385870	-1.3306000	-0.0827160
C	3.1072580	-0.5361090	0.5408640
C	2.5473460	-2.4208730	-0.8596570
C	4.4605530	-0.8162130	0.3754050
H	2.7981380	0.3006990	1.1544120
C	3.9028010	-2.6939420	-1.0325180
H	1.8036290	-3.0422970	-1.3446580
C	4.8630040	-1.8916830	-0.4172370
H	5.2012520	-0.1950760	0.8663050
H	4.2078660	-3.5317110	-1.6494180
H	5.9175570	-2.1050250	-0.5506550
N	-0.0291610	-2.1526090	0.5210430
H	0.4987490	-2.8420760	1.0388590
H	-0.9653600	-2.0134520	0.8766750
H	-1.8172080	-1.0601990	-0.9641650
H	1.4913890	0.1290700	-2.4872250
C	-3.5770180	-0.4108990	-0.1442550
C	-4.2844480	-1.3538340	-0.9061000
C	-4.2753820	0.4170520	0.7451460
C	-5.6635900	-1.4686890	-0.7807770
H	-3.7443450	-1.9947010	-1.5952680
C	-5.6591820	0.2888910	0.8602880
H	-3.7401580	1.1481910	1.3297650
C	-6.3626810	-0.6467610	0.1046810
H	-6.1924570	-2.2028000	-1.3781940
H	-6.1888270	0.9343580	1.5523540
H	-7.4384350	-0.7349390	0.2018650

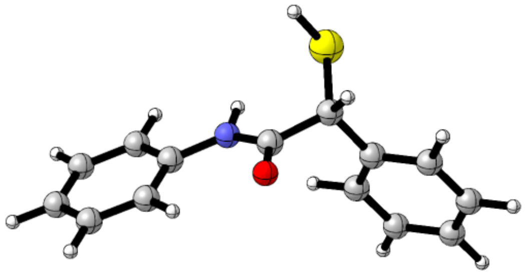

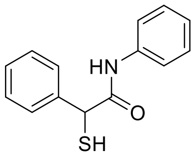

30

4a-8 0 1 scf done:-1069.799504 Sum of electronic and thermal Free Energies -1069.611334
imag. freq.: 0

C	4.4044450	-1.0579080	0.3773970
C	3.4998480	-0.0817730	0.7907180
C	2.2508540	0.0328910	0.1746030
C	1.9181860	-0.8431930	-0.8645340
C	2.8234660	-1.8180370	-1.2781890
C	4.0682790	-1.9282720	-0.6587150
H	5.3690150	-1.1387350	0.8656250
H	3.7609360	0.5961310	1.5958340
H	2.5546680	-2.4929550	-2.0828090
H	4.7705140	-2.6888260	-0.9805820
N	-1.0256670	0.5867560	-0.0073440
C	-0.0863370	0.5081130	0.9680510
O	-0.2387220	-0.0332520	2.0563730
C	1.2895250	1.1104640	0.6332460
H	1.6585080	1.5416940	1.5613070
S	1.3021710	2.4624530	-0.6421250
H	0.5699710	3.3543180	0.0636720
H	0.9524760	-0.7722570	-1.3518860
H	-0.7501090	1.1062920	-0.8346720
C	-2.3334080	0.0645010	-0.0316600
C	-3.0842800	0.2955060	-1.1936540
C	-2.8993100	-0.6598290	1.0249420
C	-4.3820000	-0.1898500	-1.2980040
H	-2.6422170	0.8552950	-2.0111320
C	-4.2028240	-1.1409340	0.9044030
H	-2.3267930	-0.8396780	1.9210720
C	-4.9510620	-0.9128010	-0.2481890
H	-4.9487950	-0.0027910	-2.2030170
H	-4.6328940	-1.7007280	1.7274970
H	-5.9631630	-1.2915750	-0.3294470

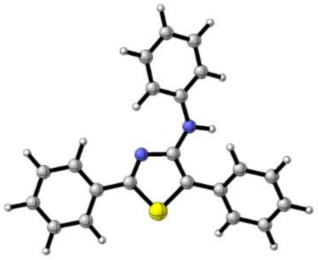

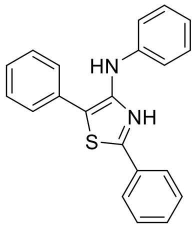

40

4a-9 0 1 scf done:-1317.954243 Sum of electronic and thermal Free Energies -1317.692920

imag. freq.: 0

C	4.3730570	-1.3456350	-1.0236100
C	3.0076520	-1.0826530	-0.9657610
C	2.5048870	-0.0734000	-0.1270260
C	3.4146510	0.6613880	0.6545940
C	4.7795840	0.4009350	0.5856590
C	5.2664250	-0.6026200	-0.2525320
H	4.7393980	-2.1278670	-1.6788190
H	2.3277660	-1.6547210	-1.5870760
H	5.4637800	0.9762830	1.1991200
H	6.3300560	-0.8053670	-0.3012830
C	0.3943050	1.3957900	0.0835930
C	1.0673210	0.1930680	-0.0498080
S	-0.1125150	-1.0985390	-0.1242110
C	-1.3998810	0.0776620	0.0739810
C	-2.8125690	-0.3036960	0.1407660
C	-3.7736700	0.6693390	0.4599050
C	-3.2330480	-1.6190960	-0.1047330
C	-5.1195130	0.3292790	0.5327350
H	-3.4547320	1.6855410	0.6524410
C	-4.5807630	-1.9541970	-0.0299300
H	-2.5125830	-2.3876290	-0.3616750
C	-5.5294660	-0.9823840	0.2887420
H	-5.8509540	1.0895080	0.7820970
H	-4.8900690	-2.9746520	-0.2238200
H	-6.5794860	-1.2449790	0.3463060
N	-0.9714780	1.3104120	0.1547880
H	3.0498910	1.4232500	1.3321920
N	1.0102160	2.6363760	0.0559390
H	1.9293800	2.6517200	-0.3640820
C	0.6718680	3.7815390	0.7792920
C	1.4491790	4.9347880	0.5702610
C	-0.3818930	3.8338940	1.7048890
C	1.1765720	6.1066420	1.2648140
H	2.2683400	4.8974950	-0.1405220
C	-0.6454310	5.0182390	2.3906880
H	-0.9903120	2.9597630	1.8839110
C	0.1229200	6.1621700	2.1800210
H	1.7911900	6.9824550	1.0875370
H	-1.4638930	5.0391580	3.1022340
H	-0.0917710	7.0778710	2.7181520

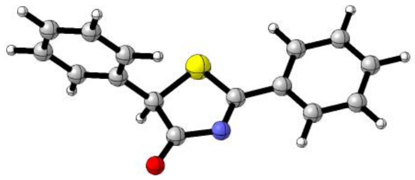

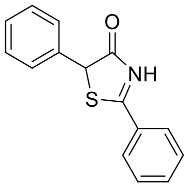

(13')

29

4a-10 0 1 scf done:-1106.696467 Sum of electronic and thermal Free Energies -1106.520003 imag. freq.: 0

C	-4.6414410	-0.2730100	-1.0532390
C	-3.4598270	-0.9958260	-1.2206570
C	-2.3005440	-0.6177870	-0.5417790
C	-2.3325540	0.4941650	0.3073040
C	-3.5109140	1.2141010	0.4748990
C	-4.6690270	0.8325520	-0.2062770
H	-5.5367200	-0.5754430	-1.5844580
H	-3.4370070	-1.8575670	-1.8785020
H	-3.5275850	2.0721500	1.1371920
H	-5.5864270	1.3949530	-0.0748510
C	-0.4039200	-1.8805300	0.6115340
O	-1.0180310	-2.5673180	1.3987710
C	-1.0319540	-1.4117480	-0.7232500
S	0.3028640	-0.4691740	-1.5660330
C	1.3719760	-0.7466510	-0.1772840
C	2.7278310	-0.1956450	-0.1768420
C	3.5989150	-0.5152930	0.8782300
C	3.1738570	0.6452870	-1.2074880
C	4.8882130	-0.0005650	0.8970290
H	3.2542120	-1.1666700	1.6703310
C	4.4646090	1.1587130	-1.1829110
H	2.5165260	0.9088750	-2.0276750
C	5.3234920	0.8369650	-0.1317420
H	5.5561550	-0.2520210	1.7123550
H	4.7998090	1.8095560	-1.9814490
H	6.3305010	1.2376780	-0.1143900
N	0.9021690	-1.4479240	0.8101450
H	-1.2303080	-2.3024290	-1.3206160
H	-1.4365970	0.7967390	0.8391930

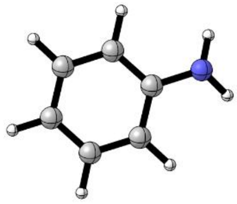

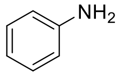

14

Aniline 0 1 scf done:-287.723325 Sum of electronic and thermal Free Energies -287.635892 imag. freq.: 0

C	-2.5121820	-0.1027190	0.0030460
C	-3.4025270	0.8683500	0.4910560
C	-3.0018700	-1.3934270	-0.2585640
C	-4.7404920	0.5526930	0.7094030
H	-3.0356550	1.8691040	0.6952900
C	-4.3417220	-1.6980190	-0.0366590
H	-2.3234420	-2.1512690	-0.6369830
C	-5.2237960	-0.7311170	0.4496900
H	-5.4108280	1.3187710	1.0843270
H	-4.6989020	-2.7002770	-0.2480860
H	-6.2664140	-0.9723470	0.6201830
N	-1.1594580	0.1914530	-0.1616600
H	-0.6675750	-0.3992040	-0.8198650
H	-0.9457280	1.1709640	-0.2993830

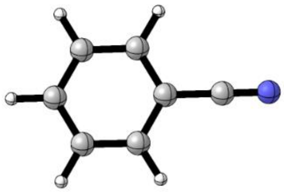

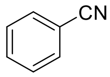

13

Benzonitrile 0 1 scf done:-324.613608 Sum of electronic and thermal Free Energies -324.544930 imag. freq.: 0

C	-2.1661780	0.1829640	0.6849520
C	-3.2569020	-0.2108120	-0.1478530
C	-4.5054630	-0.4889300	0.4281340
C	-3.0696420	-0.3158440	-1.5341850
C	-5.5623270	-0.8716680	-0.3892490
H	-4.6354320	-0.4036860	1.4996580
C	-4.1362930	-0.6997620	-2.3381930
H	-2.1004220	-0.0980980	-1.9649070
C	-5.3794980	-0.9771270	-1.7685410
H	-6.5288020	-1.0876980	0.0499860
H	-3.9975540	-0.7825620	-3.4094380
H	-6.2075280	-1.2760680	-2.4007750
N	-1.2828930	0.5018680	1.3593710

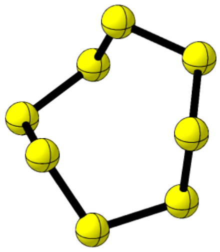

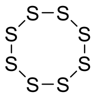

8

S_8 0 1 scf done:-3185.802156 Sum of electronic and thermal Free Energies -3185.830556 imag. freq.: 0

S	-0.1189120	1.3415240	-1.7606760
S	0.9091380	-0.4353310	-2.1578950
S	-0.5003860	-2.0236470	-1.7278510
S	-1.4877350	-1.4748750	0.0205220
S	-0.4633130	-2.0110380	1.7489100
S	0.9505660	-0.4142700	2.1420300
S	-0.0872460	1.3559210	1.7444010
S	0.7978890	2.1058900	-0.0194530

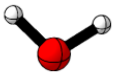

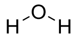

3

H_2O 0 1 scf done:-76.467526 Sum of electronic and thermal Free Energies -76.464204 imag. freq.: 0

O	-0.0143780	-0.0203440	0.0000000
H	0.9499460	0.0223580	-0.0000000
H	-0.2960230	0.9029220	0.0000000

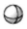

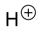

1

H^+ 1 1 scf done:-0.213677 Sum of electronic and thermal Free Energies -0.223677 imag. freq.: 0

H	-1.8870440	0.1986360	0.0000000

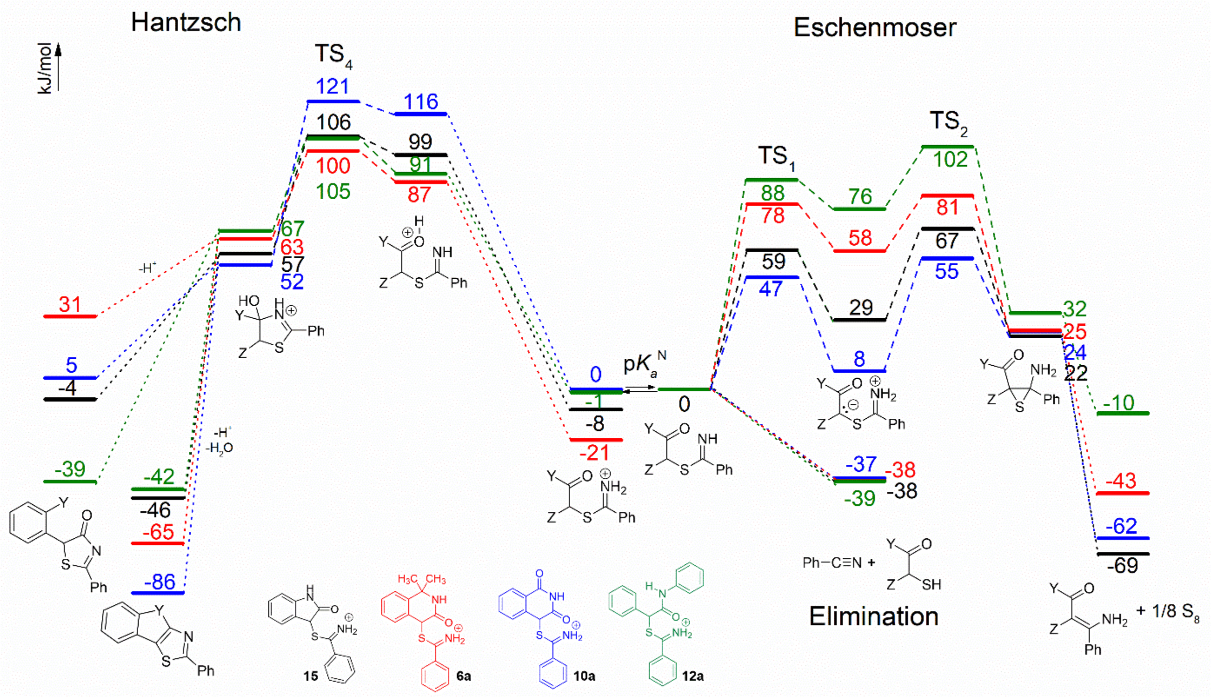

Extended version of Figure 2 in the main text - Comparison of energy profiles (relative Gibbs energies at 298 K in kJ·mol^{-1} for the ECR (right) and Hantzsch (left) pathways for salts **6a**, **10a**, **12a**, and **15** calculated at the B3LYP-D3/6-311++G(d,p) level of theory in *N*,*N*-dimethylformamide (SMD).

References

1. Barbry, D.; Sokolowski, G.; Champagne, P. *Synth. Commun.* **2002**, *32*, 1787–1790.
2. Bamba M., Furuyama H., Niiyama K., Sakamoto T., Sunami S., Takahashi K., Yamamoto F., Yoshizumi T. (Merck & Co. Inc.), Bicycloaniline derivative. EP2168966, 2010, A1.
3. Lee, C.-H.; Bayburt, E. K.; Di Domenico Jr., S.; Drizin, I.; Gomtsyan, A. R.; Koenig, J. R.; Perner R. J.; Schmidt Jr., R. G.; Turner, S. C.; Jinkerson, T. K.; Zheng, G. Z. U. S. Patent 2005/0113576 A1, May 26, 2005.
4. Hu, K.; Qi, L.; Yu, S.; Cheng, T.; Wang, X.; Li, Z.; Xia, Y.; Chen, J.; Wu, H. *Green Chem.* **2017**, *19*, 1740−1750.
5. Berry, J. P.; Isbell, A. F.; Hunt G. E. *J. Org. Chem.* **1972**, *37*, 4396–4399.
6. Gotthardt, H.; Pflaumbaum, W. *Chem. Ber.* **1987**, *120*, 1017–1022.
7. Hoffman, R. V.; Nayyar, N. K.; Chen, W. *J. Org. Chem.* **1992**, *57*, 5700–5707.
8. Kammel, R.; Tarabová, D.; Machalický, O.; Nepraš, M.; Frumarová, B.; Hanusek, J. *Dyes Pigments* **2016**, *128*, 101–110.
